# Safety and performance of the third-generation drug-eluting resorbable coronary magnesium scaffold system in the treatment of subjects with de novo coronary artery lesions: 6-month results of the prospective, multicenter BIOMAG-I first-in-human study

**DOI:** 10.1016/j.eclinm.2023.101940

**Published:** 2023-04-17

**Authors:** Michael Haude, Adrian Wlodarczak, René J. van der Schaaf, Jan Torzewski, Bert Ferdinande, Javier Escaned, Juan F. Iglesias, Johan Bennett, Gabor Toth, Michael Joner, Ralph Toelg, Marcus Wiemer, Göran Olivecrona, Paul Vermeersch, Hector M. Garcia-Garcia, Ron Waksman

**Affiliations:** a Medical Clinic I, Rheinland Klinikum Neuss GmbH, Lukaskrankenhaus, Neuss, Germany; b Department of Cardiology, Miedziowe Centrum Zdrowia SA, Lubin, Poland; c Department of Interventional Cardiology, OLVG, Amsterdam, the Netherlands; d Cardiovascular Center Oberallgäu-Kempten, Germany; e Department of Cardiology, Ziekenhuis Oost Limburg (ZOL), Genk, Belgium; f Division of Cardiology, Hospital Clinico San Carlos IDISSC, Complutense University of Madrid, Madrid, Spain; g Cardiology Division, University Hospital of Geneva, Geneva, Switzerland; h Department of Cardiovascular Medicine, University Hospitals Leuven, Leuven, Belgium; i Division Cardiology, Medical University Graz, Graz, Austria; j Klinik für Herz- und Kreislauferkrankungen, Deutsches Herzzentrum München, München, Germany; k Deutsches Zentrum für Herz- und Kreislauf-Forschung (DZHK) e.V. (German Center for Cardiovascular Research), Partner Site Munich Heart Alliance, Munich, Germany; l Cardiology Department, Heart Center Segeberger Kliniken, Bad Segeberg, Germany; m Department of Cardiology and Intensive Care, Johannes Wesling University Hospital Ruhr University Bochum, Minden, Germany; n Department of Cardiology, Skane University Hospital, Lund, Sweden; o Interventional Cardiology ZNA Middelheim, Antwerpen, Belgium; p Interventional Cardiology, MedStar Washington Hospital Center, Washington DC, USA

**Keywords:** Bioresobabe scaffolds, Coronary artery disease, Drug-eluting stents

## Abstract

**Background:**

A third-generation coronary drug-eluting resorbable magnesium scaffold (DREAMS 3G) was developed to enhance the performance of previous scaffold generations and achieve angiographic outcomes comparable to those of contemporary drug-eluting stents.

**Methods:**

This prospective, multicenter, non-randomized, first-in-human study was conducted at 14 centers in Europe. Eligible patients had stable or unstable angina, documented silent ischemia, or non-ST-elevation myocardial infarction, and a maximum of two single de novo lesions in two separate coronary arteries with a reference vessel diameter between 2.5 mm and 4.2 mm. Clinical follow-up was scheduled at one, six and 12 months and annually thereafter until five years. Invasive imaging assessments were scheduled six and 12 months postoperatively. The primary endpoint was angiographic in-scaffold late lumen loss at six months. This trial was registered at ClinicalTrials.gov (NCT04157153).

**Findings:**

Between April 2020 and February 2022, 116 patients with 117 coronary artery lesions were enrolled. At six months, in-scaffold late lumen loss was 0.21 mm (SD 0.31). Intravascular ultrasound assessment showed preservation of the scaffold area (mean 7.59 mm^2^ [SD 2.21] post-procedure vs 6.96 mm^2^ [SD 2.48]) at six months) with a low mean neointimal area (0.02 mm^2^ [SD 0.10]). Optical coherence tomography revealed that struts were embedded in the vessel wall and were already hardly discernible at six months. Target lesion failure occurred in one (0.9%) patient; a clinically driven target lesion revascularization was performed on post-procedure day 166. No definite or probable scaffold thrombosis or myocardial infarction was observed.

**Interpretation:**

These findings show that the implantation of DREAMS 3G in de novo coronary lesions is associated with favorable safety and performance outcomes, comparable to contemporary drug-eluting stents.

**Funding:**

This study was funded by BIOTRONIK AG.

**Research in context**

**Evidence before this study**

We performed a literature search on PubMed to identify meta-analyses of published reports on coronary scaffolds and previous publications on the precursor devices of DREAMS 3G. The search was conducted on April 28, 2022, using the search terms (coronary artery disease) AND scaffold, filter past 5 years, meta-analysis, and (coronary artery disease) AND (DREAMS OR Magmaris) AND scaffold, filter past 5 years. The search was updated on March 16, 2023. Indications other than those of BIOMAG-I (e.g., peripheral artery disease) and studies involving <50 patients were excluded. No language filter was used. The identified meta-analyses included the ABSORB-II, ABSORB-III, ABSORB Japan, ABSORB China, AIDA, TROFI-II, and EVERBIO-II trials, while studies using the DREAMS 2G scaffold included the BIOSOLVE-II, -III, and -IV trials, the Magnesium 2000 postmarket evaluation, the Magmaris Multicenter Italian Registry, a comparison to the Ultimaster stent (Ultimaster, Terumo, Japan), and a SICI-GISE position paper. Since the latter trials were not randomized, there is a risk of bias due to different patient populations.

**Added value of this study**

This is the first report on a third-generation drug-eluting resorbable Magnesium scaffold. Compared with its precursors, DREAMS 3G showed substantially improved angiographic LLL while maintaining a favorable safety profile with the absence of definite or probable scaffold thrombosis and low TLF and TLR rates.

**Implications of all the available evidence**

The improved angiographic parameters observed have now reached levels of contemporary DES. Based on the data obtained from BIOMAG-I, a prospective randomized controlled trial will be initiated, comparing DREAMS 3G with a contemporary DES. These data, along with long-term data, will show whether the promising outcomes of BIOMAG-I can be translated into broad clinical practice and which patient population will benefit most from treatment with DREAMS 3G.

## Introduction

Compared to bare-metal stents, drug-eluting stents (DES) have significantly lower restenosis rates after percutaneous coronary intervention (PCI) as they prevent excessive neointimal proliferation. However, permanent implants have several limitations, such as hypersensitivity reactions, delayed arterial healing, and neoatherosclerosis, which ultimately increase the risk of late stent thrombosis and restenosis.^1^

Bioresorbable scaffolds have been developed to provide mechanical support and control neointimal proliferation over the vascular healing period, disappearing thereafter and thus preventing long-term stent-related adverse events.^1^^,^^2^ In contrast to poly l-lactide acid (PLLA) scaffolds, metallic resorbable magnesium scaffolds have emerged as a promising technology because of characteristics such as metal-like behavior during implantation, shorter resorption time, and less thrombogenicity with electropolished rounded edges, facilitating scaffold embedding in the vessel wall.^1^^,^^3^^,^^4^

The second-generation Drug-Eluting Resorbable Magnesium Scaffold (DREAMS 2G, commercial name Magmaris) obtained a CE mark in 2016 and has shown low target lesion failure (TLF) and scaffold thrombosis rates in multiple trials.^5^, ^6^, ^7^, ^8^ Nevertheless, in-scaffold late lumen loss (LLL) with DREAMS 2G was not comparable to that observed with historical PLLA-based scaffolds or contemporary DES.^9^^,^^10^ This led to the development of the third-generation drug-eluting magnesium scaffold (DREAMS 3G), which has a new magnesium alloy allowing improved mechanical properties and thinner struts, a new marker concept, and an increased size portfolio, with the resorption time remaining unchanged.

The BIOTRONIK – Safety and Clinical Performance of the Sirolimus-Eluting Resorbable Coronary Magnesium Scaffold System (DREAMS 3G) in the Treatment of Subjects With de Novo Lesions in Native Coronary Arteries (BIOMAG-I) study aimed to assess the safety and performance of DREAMS 3G in patients with de novo coronary lesions. This is the first study conducted with this novel device, and the data will be used to support CE certification.

## Methods

### Study design and patients

This prospective, multicenter, non-randomized, first-in-human trial was conducted at 14 centers in Austria, Belgium, Germany, the Netherlands, Poland, Spain, Sweden, and Switzerland (Supplemental Table S1). Eligible patients had symptomatic coronary artery disease with stable or unstable angina, documented silent ischemia, or non-ST-elevation myocardial infarction (NSTEMI). A maximum of two de novo single lesions in two separate coronary arteries was permitted, with reference vessel diameters between 2.5 mm and 4.2 mm, a maximum lesion length ≤28 mm, and a diameter stenosis between 50% and 100%. The exclusion criteria included left main disease, ST-elevation myocardial infarction, unsuccessful predilatation, or ostial lesions. The full list of inclusion and exclusion criteria is provided in Supplemental Table S2.

The study was conducted in accordance with the current version of the Declaration of Helsinki, ISO14155, and local guidelines. This study was approved by the ethics committees of all the participating institutions. All patients provided written informed consent prior to any study procedure. The completeness and quality of the data were ensured through monitoring with 100% source document verification. A scientific steering committee was responsible for protocol development and supervision of the study conduct, and a data monitoring committee was responsible for review and adjudication of all endpoint-related events and for periodic safety reviews. A core laboratory (MedStar Cardiovascular Research Network, Washington Hospital Center, Washington, DC, USA) was used for independent angiographic, intravascular ultrasound (IVUS), and optical coherence tomography (OCT) assessments. This trial was registered at ClinicalTrials.gov (NCT04157153).

### Procedure

DREAMS 3G is a scaffold system consisting of a balloon-expandable resorbable scaffold on a rapid-exchange delivery system. The scaffold backbone is made from a resorbable magnesium alloy and contains two permanent X-ray markers made from tantalum on its distal and proximal ends. Magnesium degrades to amorphous calcium phosphate via magnesium hydroxide and magnesium phosphate, and resorption is completed within 12 months. The surface of the scaffold backbone is completely coated with bioresorbable PLLA which incorporates Sirolimus (1.4 ± 0.3 μg per mm^2^ scaffold surface) as the antiproliferative drug. The device is available in diameters of 2.5, 3.0, 3.5 and 4.0 mm and lengths of 13, 22, and 30 mm (the 30-mm length is only available for diameters 3.0 and 3.5 mm).

Compared to its precursor (DREAMS 2G), DREAMS 3G has the following design improvements: (a) an increased radial strength through a modification in the magnesium alloy, (b) increased scaffold marker X-ray visibility, (c) a larger size range, and (d) thinner struts: 99 μm for Ø 2.5 mm, 117 μm for Ø 3.0 and 3.5 mm, and 147 μm for Ø 4.0 mm. An in vivo investigation using a porcine model showed that DREAMS 3G provided vessel support for approximately 3–6 months and released approximately 70% of the sirolimus in the PLLA carrier during three months after implantation.

Implantation had to be performed in accordance with the “4P” strategy,^11^ including adequate patient selection, proper sizing, adequate pre-dilatation (non-compliant balloon, 1:1 balloon-to-artery-ratio, recommended residual stenosis before implantation of ≤20%), and post-dilatation with a non-compliant balloon maximally 0.5 mm larger than the implanted nominal scaffold diameter and expanded at high pressure (>16 atm). In the event of incomplete lesion coverage or dissection, a second DREAMS 3G was allowed to be placed end-to-end, while overlapping should be avoided. Dual antiplatelet therapy is recommended for at least six months, followed by single antiplatelet therapy.

Clinical follow-up visits were scheduled at one, six, and 12 months and annually thereafter, until 60 months after the procedure. Angiographic, IVUS (including virtual histology if available), and OCT assessments were scheduled pre- and post-procedure, as well as at six and 12 months. The instructions for image acquisition are provided in Supplemental Table S3.

### Outcomes

Our primary endpoint was in-scaffold LLL at six months calculated from quantitative coronary angiography (QCA). The secondary angiographic endpoints were in-scaffold LLL at 12 months and in-segment LLL, binary in-scaffold and in-segment restenosis, and in-scaffold and in-segment diameter stenosis at six and 12 months. The IVUS and OCT endpoints included a descriptive analysis of vessel morphology, lesion composition, and scaffold strut data. Procedure success was defined as achievement of a final diameter stenosis of <30% by QCA, using any percutaneous method, without the occurrence of death, Q-wave or non-Q-wave myocardial infarction, or target lesion revascularization (TLR) during hospital stay. Device success was defined as a final residual diameter stenosis of <30% by QCA or visual assessment using the assigned device only in cases of successful delivery of the scaffold to the target lesion, appropriate scaffold deployment, and successful removal of the delivery system.

Our secondary clinical endpoints, which were evaluated at each follow-up visit, included TLF, a composite of cardiac death, target-vessel myocardial infarction (TV-MI), periprocedural MI adjudicated according to SCAI, spontaneous MI according to extended historical definitions,^12^^,^^13^ and clinically driven TLR (CD-TLR); the individual components of TLF; clinically driven target-vessel revascularization (CD-TVR); and definite or probable scaffold thrombosis.^14^ Further definitions are provided in Supplemental Table S3 and S4.

### Statistical analysis

BIOMAG-I assesses the non-inferiority of the 6-month in-scaffold LLL with DREAMS 3G compared with the historical control of clinical trials of PLLA and metallic scaffolds (Supplemental Table S5 and S6). Presuming an in-scaffold loss of 0.29 mm (standard deviation, SD of 0.34 mm), a non-inferiority margin of 0.145 mm, a power of 0.95, an alpha of 0.025, and a drop-out rate of 15%, a sample size of 88 patients was calculated. For in-scaffold LLL at 12 months (a secondary powered endpoint), a sample size of 104 was calculated presuming a weighted mean of 0.33 mm (SD, 0.35) with the same non-inferiority margin, power, and alpha and considering a 25% drop out rate. The sample size was calculated using the PASS 15 software package (NCSS, LLC. Kaysville, Utah, USA; ncss.com/software/pass). To allow for potential further attrition (i.e., no follow-up angiography or poor image quality making quantitative angiographic LLL calculation difficult), an additional drop-out margin was defined, resulting in a total sample size of 115. In addition, a post-hoc non-inferiority analysis of in-scaffold LLL was performed against PLLA scaffolds, and a superiority analysis was performed against DREAMS 2G (both with power >90%). The t-test was used to assess statistical significance. In another post-hoc analysis, subgroups were compared using the Wilcoxon test (Supplemental Table S7).

Normal distribution was assessed with the Shapiro–Wilk test, Kolmogorov–Smirnov and QQ-Plot. The primary endpoint was evaluated using a one-sided, one-sample t-test as specified in the initial sample size calculation.

Means, standard deviations (SD), and 95% confidence intervals (CIs) and medians with interquartile ranges (IQRs) were calculated. For categorical data, we calculated absolute and relative frequencies with 95% CIs for proportions based on the data available. Kaplan–Meier analyses were used to assess clinical outcomes. Imaging parameters were compared using the t-test. We analyzed the intention-to-treat population, defined as patients in whom an investigational scaffold entered the guide catheter after the diagnostic angiogram. Statistical analyses were performed using SAS software version 9.4 (SAS Institute Inc. Cary, NC, USA).

### Role of the funding source

This study was funded by BIOTRONIK AG (Buelach, Switzerland). The funder was involved in the trial design, data collection, analysis, and interpretation, and reimbursement of the costs for a medical writer and open access. The authors, the steering committee, the sponsor and the medical writer had access to the dataset. The sponsor was not involved in the decision to submit for publication; the primary decision to submit for publication was the first author's decision; all authors agreed to submit for publication.

## Results

**Fig. 1:**
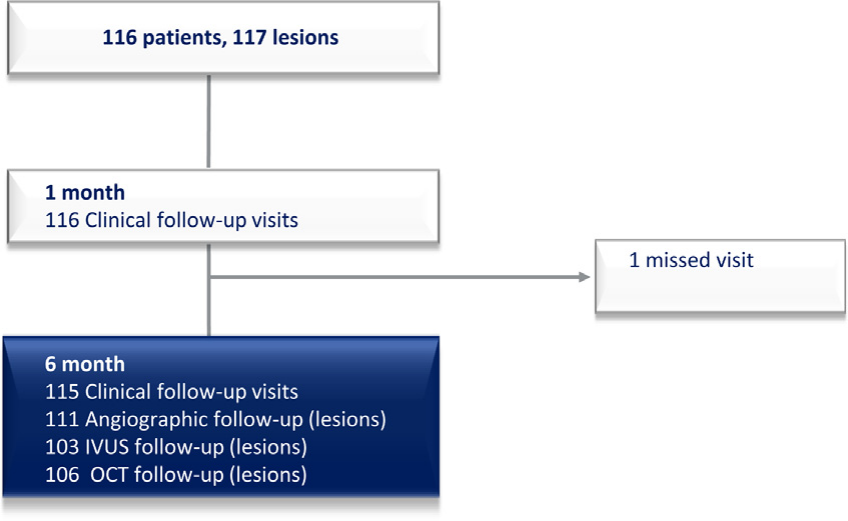
**Patient flow chart.** Paired data are available for 111 lesions with angiographic follow-up, for 87 lesions with IVUS follow-up and 101 lesions with OCT follow-up. IVUS: intravascular ultrasound, OCT: optical coherence tomography.

We enrolled 116 patients with 117 lesions between April 2020 and February 2022 (Fig. 1).

Pre-dilatation was performed in all lesions, and the residual diameter stenosis before scaffold placement was 10.8% (SD 6.4) (range 0–20%). More than one scaffold was implanted in 11 lesions. The reasons for multiple scaffold implants were dissection (n = 8), device not implanted at the intended site (n = 1), a distal residual stenosis of 20% (n = 1), and incomplete lesion coverage (n = 1). The scaffolds were placed end-to-end in most cases (n = 9), while in one case there was scaffold overlap and in another case a gap of 2 mm between scaffolds.). The mean scaffold length and diameter was 22.1 mm (SD 5.6) and 3.28 mm (SD 0.47), respectively. Postdilatation was performed in all cases with a postdilatation balloon diameter-to-scaffold diameter ratio of 1.09 (SD 0.06).

**Fig. 2:**
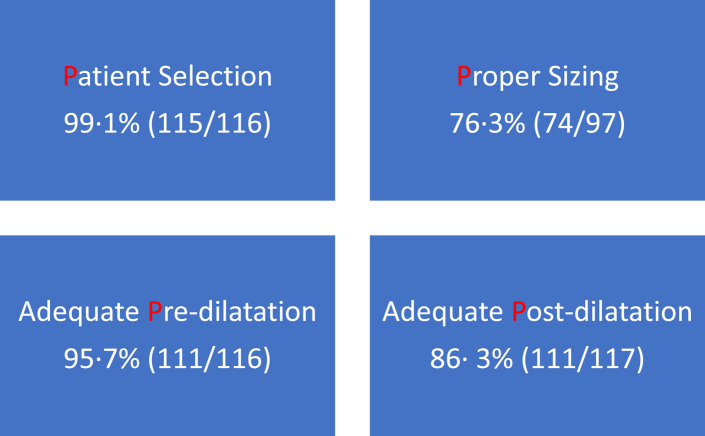
**Adherence to the 4P principles.** Patient selection (all in- and exclusion criteria fulfilled). Adequate pre-dilatation: all lesions were pre-dilated with a residual stenosis ≤20%; a 1:1 balloon-to-artery-ratio (max diameter of predilatation balloon should be lower than reference vessel diameter+0.25 mm) was achieved in 95.7%. Proper sizing: sizing within the range in the instructions for use. Adequate post-dilatation: all patients received a post-dilatation with a non-compliant balloon, but only 101/117 lesions were post-dilatated with ≥16atm.

The scaffold to reference vessel diameter ratio was 0.997 (SD 0.011, 95%CI: 0.990–1.004) for device diameter 2.5 mm, 0.995 (SD 0.020, 95%CI: 0.990–1.001) for device diameter 3.0 mm, 1.000 (SD 0.007, 95%CI: 1.000–1.003) for device diameter 3.5 mmm, and 0.999 (SD 0.011, 95%CI: 0.994–1.004) for device diameter 4.0 mm. Underexpansion (device expansion index <0.8) was reported for 15.5% (15/97), optimal expansion (device expansion index 0.8–1.0) for 76.3% (74/97), and overexpansion (device expansion index >1) was reported for 8.2% (8/97) (Supplemental Fig. S1). The 4P strategy was largely adhered to as displayed in Fig. 2.

Device success was achieved in 97.7% of devices (126/129); one lesion could not be crossed, residual stenosis was 30% in one case, and one device could not be implanted at the intended site and required a second scaffold, as described above. Procedure success was reported in 99.1% (115/116) of the patients; one patient had failed device success with a residual stenosis of 30%. The baseline characteristics of the patients are summarized in Table 1.

**Table 1:** Baseline characteristics.

All patients	N = 116
Age (years)	61.0 (9.0)
Sex	
Male	90 (77.6%)
Female	26 (22.4%)
Hypertension	86 (74.1%)
Hypercholesterolemia	72 (62.1%)
Diabetes	32 (27.6%)
Insulin-dependent diabetes	6 (5.2%)
History of smoking	75 (64.7%)
History of myocardial infarction	39 (33.6%)
Renal failure	1 (0.9%)
Congestive heart failure	10 (8.6%)
History of stroke or transient ischemic attack	6 (5.2%)
NSTEMI	24 (20.7%)
Ischemic status (n = 92, excluding NSTEMI patients)	
Stable angina	49 (53.3%)
Unstable angina	15 (16.3%)
Silent ischemia	28 (30.4%)
**All lesions (core laboratory data)**	**N** = **117**
Lesion length (mm)	12.3 (5.1)
Reference vessel diameter (mm^2^)	2.72 (0.46)
Target vessel	
Left anterior descending	53 (45.3%)
Left circumflex artery	22 (18.8%)
Right coronary artery	40 (34.2%)
Intermediate branch	2 (1.7%)
AHA/ACC classification	
Type A	3 (2.6%)
Type B1	24 (20.5%)
Type B2	57 (48.7%)
Type C	33 (28.2%)
Calcification	
Moderate to severe	3 (2.6%)
Thrombus present	0 (0.0%)
Side branch involvement	25 (21.4%)

Data are mean (SD), or n (%). AHA/ACC: American Heart Association/American College of Cardiology, NSTEMI: non-ST-elevation myocardial infarction, PCI: percutaneous coronary intervention.

At the 6-month follow-up, 82.6% (95/115) of patients had no pathological findings, 16.5% (19/115) had stable angina, and 0.9% (1/115) had documented silent ischemia. Nearly all patients were on dual antiplatelet therapy at 6 months (97.4%, Supplemental Table S8).

**Fig. 3:**
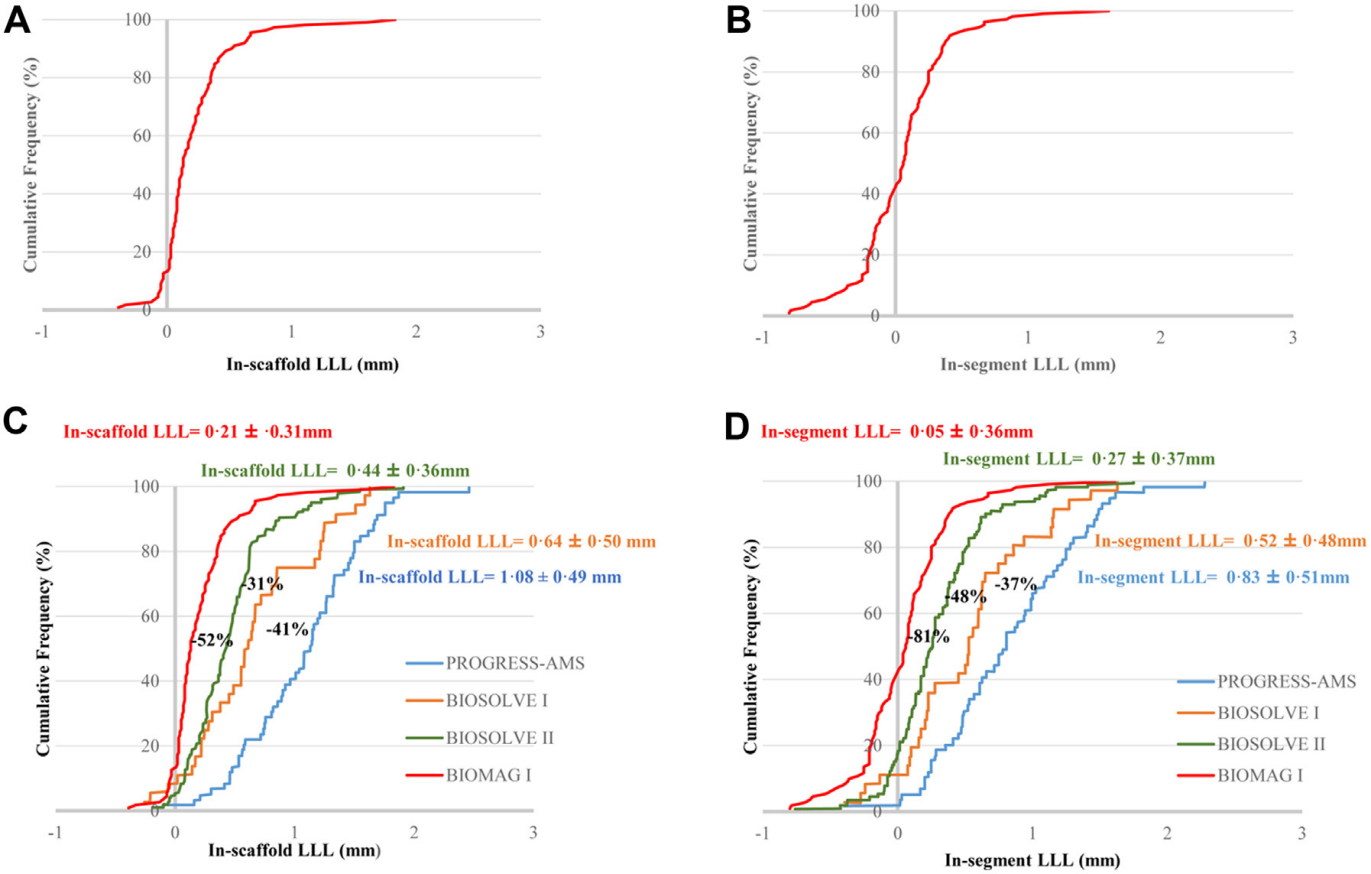
**Cumulative frequency curves for late lumen loss.** In-scaffold (A) and in-segment (B) late lumen loss, and in-scaffold (C) and in-segment (D) late lumen loss compared with PROGRESS-AMS,^15^ BIOSOLVE-I,^16^ and BIOSOLVE-II.^3^

Angiographic follow-up at six months was available for 110 patients (111 lesions). The primary endpoint, mean angiographic in-scaffold LLL at 6 months post-procedure, was 0.21 mm (SD 0.31, 95%CI 0.16–0.27); the median in-scaffold LLL was 0.13 mm (IQR: 0.05; 0.32). Thus, the null hypothesis was rejected, and non-inferiority to the historical control of resorbable scaffolds was demonstrated (weighted mean 0.29 mm, non-inferiority margin 0.145 mm, power 95.0%, p < 0.001). Moreover, in a post-hoc analysis, superiority to the precursor–DREAMS 2G (weighted mean 0.44 mm, 0.11 mm superiority margin, calculated power 93.6%, p < 0.001) and non-inferiority to PLLA-based scaffolds (weighted mean 0.22 mm, 0.11 mm non-inferiority margin, calculated power 93.6%, p < 0.001) were confirmed (Supplemental Table S5). The mean in-segment LLL was 0.05 mm (SD 0.36, 95%CI -0.01–0.12) (Fig. 3, Table 2). Considering the differences in baseline parameters between BIOMAG-I and the comparator studies, we assessed the impact on NSTEMI, type B2/C lesions, age, and minimum lumen diameter on the primary endpoint, in-device LLL, and found no statistically significant differences amongst the groups (Supplemental Table S7).

**Table 2:** Core laboratory assessed imaging analysis – paired data.

	Pre-procedure	Post-procedure	6 months	Δ post-procedure – 6 months paired data	p-value post-procedure vs 6 months
**Angiography**	**N = 111**	**N = 111**	**N = 111**	**N = 111**	
Reference vessel diameter (mm)					
In-scaffold	NA	2.85 (0.46)	2.84 (0.49)	−0.01 (0.26)	0.73
		2.83 (2.51; 3.17)	2.80 (2.49; 3.18)		
In-segment	2.73 (0.46)	2.75 (0.50)	2.79 (0.51)	0.05 (0.33)	0.14
	2.71 (2.39; 3.05)	2.72 (2.39; 3.07)	2.74 (2.45; 3.13)		
Minimum lumen diameter (mm)					
In-scaffold	NA	2.60 (0.43)	2.39 (0.54)	−0.21 (0.31)	<0.0001
		2.59 (2.26; 2.92)	2.39 (2.05; 2.73)		
In-segment	1.06 (0.39)	2.31 (0.44)	2.26 (0.49)	−0.05 (0.36)	0.12
	1.01 (0.81; 1.30)	2.28 (1.98; 2.55)	2.19 (1.94; 2.55)		
Acute gain (mm)					
In-scaffold	NA	1.54 (0.48)	NA	NA	NA
		1.55 (1.20; 1.83)			
In-segment	NA	1.24 (0.50)	NA	NA	NA
		1.20 (0.87; 1.66)			
Diameter stenosis (%)					
In-scaffold	NA	8.4 (5.4)	16.0 (11.6)	7.5 (11.0)	<0.0001
	60.8 (12.9)	8.0 (5; 11)	14.0 (8.0; 20.0)		
In-segment	61 (51; 70)	15.6 (8.2)	19.0 (11.6)	3.3 (13.2)	0.01
		14 (10; 21)	17 (11; 24)		
Binary restenosis (%)					
In-scaffold	NA	NA	3 (2.7)^a^	NA	NA
In-segment	NA	NA	3 (2.7)^a^	NA	NA
Late lumen loss (mm)					
In-scaffold	NA	NA	0.21 (0.31)	NA	NA
			0.13 (0.05; 0.32)		
In-segment	NA	NA	0.05 (0.36)	NA	NA
			0.06 (−0.16; 0.24)		
**Intravascular ultrasound**	**N** = **87**	**N** = **87**	**N** = **87**	**N** = **87**	
Mean vessel area (mm^2^)	12.59 (4.48)	11.31 (9.10; 15.63)	14.67 (4.82)	−0.23 (1.45)	0.15
	14.90 (4.62)	14.19 (11.27; 17.76)	13.98 (11.38; 16.64)		
Mean scaffold area (mm^2^)	NA	7.59 (2.21)	6.96 (2.48)	−0.62 (1.05)	<0.0001
		7.19 (6.11; 8.90)	6.56 (5.17; 8.14)		
Minimum scaffold area (mm^2^)	NA	6.45 (2.01)	5.00 (1.97)	−1.45 (1.18)	<0.0001
		6.04 (5.20; 7.89)	4.76 (3.58; 5.74)		
Mean lumen area (mm^2^)	5.93 (2.23)	7.61 (2.26)	6.97 (2.50)	−0.65 (1.05)	<0.0001
	5.48 (4.23; 7.40)	7.19 (6.11; 9.00)	6.48 (5.20; 8.09)		
Minimum lumen area (mm^2^)	3.19 (1.27)	6.46 (2.03)	4.97 (1.98)	−1.48 (1.19)	<0.0001
	2.79 (2.37; 3.68)	6.04 (5.20; 7.89)	4.74 (3.51; 5.74)		
Mean plaque area (mm^2^)	6.63 (3.19)	7.24 (2.86)	7.72 (2.75)	0.48 (1.14)	0.0002
	5.96 (4.61; 8.30)	6.69 (5.41; 9.00)	7.16 (6.01; 9.34)		
Total incomplete strut apposition area (mm^2^)	NA	0.03 (0.10)	0.02 (0.05)	0.00 (0.10)	0.68
		0.0 (0.0; 0.0)	0.0 (0.0; 0.02)		
Device eccentricity index	NA	0.89 (0.03)	NA	NA	NA
		0.89 (0.88; 0.91)			
**Virtual histology**	**N** = **19**	**N** = **19**	**N** = **19**	**N** = **19**	
Fibrous plaque area (mm^2^)	2.33 (1.97)	1.39 (0.87)	2.21 (1.27)	0.81 (1.05)	0.003
	1.77 (1.00; 3.05)	1.27 (0.68; 2.14)	2.02 (1.64; 2.82)		
Fibrous plaque (%)	53.84 (12.08)	35.36 (13.10)	41.97 (7.07)	6.61 (10.83)	0.02
	56.89 (49.63; 60.44)	33.99 (26.87; 43 86)	44.38 (36.25; 46.03)		
Fibrous fatty plaque area (mm^2^)	0.84 (0.87)	0.26 (0.22)	0.57 (0.52)	0.32 (0.51)	0.01
	0.69 (0.31; 1.12)	0.18 (0.08; 0.42)	0.47 (0.18; 0.70)		
Fibrous fatty plaque (%)	18.07 (8.37)	7.00 (6.31)	9.69 (4.70)	2.70 (6.73)	0.10
	18.37 (13.98; 25.79)	5.70 (2.36; 8.38)	8.94 (6.24; 12.33)		
Necrotic core area (mm^2^)	0.63 (0.57)	1.05 (0.67)	1.25 (0.65)	0.20 (0.34)	0.02
	0.51 (0.22; 0.91)	1.05 (0.60; 1.50)	1.17 (0.86; 1.52)		
Necrotic core (%)	12.73 (7.58)	23.36 (8.15)	23.94 (4.18)	0.58 (7.18)	0.73
	10.67 (7.08; 16.17)	25.63 (20.73; 29.39)	24.66 (21.34; 28.01)		
Dense calcium area (mm^2^)	0.30 (0.29)	1.16 (0.82)	1.07 (0.57)	−0.09 (0.56)	0.48
	0.21 (0.08; 0.45)	1.09 (0.39; 1.59)	0.95 (0.66; 1.48)		
Dense calcium (%)	7.22 (7.58)	28.17 (13.88)	23.28 (7.83)	−4.89 (14.16)	0.15
	5.03 (1.74; 10.2)	28.71 (18.07; 36.21)	22.38 (17.24; 29.47)		
Media area (mm^2^)	2.60 (0.60)	3.39 (1.88)	3.26 (0.62)	−0.13 (1.71)	0.74
	2.63 (2.27; 2.89)	3.08 (2.82; 3.38)	3.16 (2.88; 3.81)		
**Optical coherence tomography**	**N** = **101**	**N** = **101**	**N** = **101**	**N** = **101**	
Number of struts	NA	209.1 (72.9)	NA^b^	NA^b^	NA
		206 (166; 251)			
Mean scaffold area (mm^2^)	NA	8.72 (2.52)	NA^b^	NA^b^	NA
		8.20 (6.79; 10.60)			
Mean lumen area (mm^2^)	5.61 (2.09)	8.67 (2.53)	6.95 (2.66)	−1.71 (1.67)	<0.0001
	5.42 (3.90; 6.81)	8.16 (6.71; 10.50)	6.29 (5.20; 8.27)		
Minimum lumen area (mm^2^)	2.01 (0.87)	7.20 (2.23)	4.84 (2.31)	−2.36 (1.70)	<0.0001
	1.84 (1.35; 2.46)	6.99 (5.42; 8.87)	4.29 (3.31; 6.05)		
Malapposed struts (%)	NA	4.40 (4.54)	NA^b^	NA^b^	NA
		3.58 (0.76; 6.51)			
Total incomplete strut apposition area (mm^2^)	NA	0.08 (0.11)	0.00 (0.00)	0.0 (0.0; 0.0)	<0.0001
		0.04 (0.01; 0.12)	−0.08 (0.11)		
Total tissue protrusion (mm^2^)	NA	0.13 (0.11)	NA	NA	NA
		0.09 (0.04; 0.19)			

Data are mean (SD), median (IQR), or n (%).

ISA: incomplete scaffold apposition. ISR: Incomplete strut apposition, NA: not applicable, NSTEMI: non-ST-elevation myocardial infarction, PCI: percutaneous coronary intervention.

a Diameter stenosis of 51%, 51%, and 63%.

b Struts were hardly discernable anymore at six months.

Furthermore, comparing the different imaging modalities, we assessed the in-scaffold LLL in 69 patients who had analyzable angiographic (QCA), IVUS and OCT data available. In these patients, in-scaffold LLL was 0.22 ± 0.30 mm (QCA), 0.24 ± 0.20 mm (IVUS), and 0.53 ± 0.42 mm (OCT), respectively.

In addition, IVUS assessment showed preservation of the scaffold area with a low neointimal area (Table 2). As seen on virtual histology, the difference in white color (a surrogate for scaffold struts) at six months compared to immediately post-procedure was −4.89% [SD 14.16, 95%CI -11.72 1.93], p = 0.08.

**Fig. 4:**
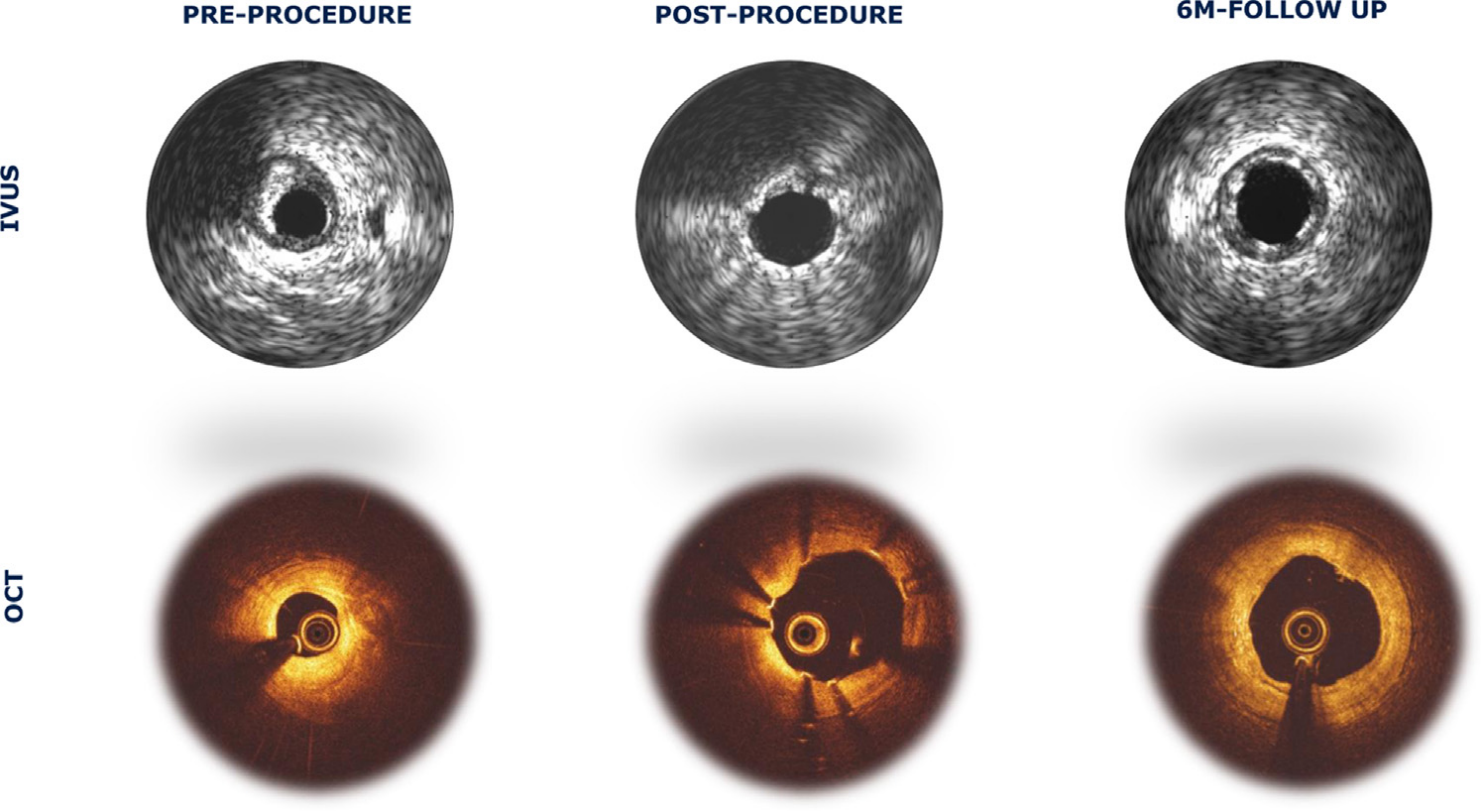
**Case examples of intravascular imaging assessments during procedure and at 6-month follow-up.** Immediately after implantation, struts are well-apposed to the vessel wall. At six months, scaffold struts are not discernible by optical coherence tomography (OCT) anymore, but by intravascular ultrasound (IVUS).

OCT revealed that struts were embedded into the vessel wall and hardly discernible anymore (Fig. 4, Table 2).

Clinical follow-up at six months was available for 99.1% (115/116) of the patients. All patients were alive, and one case of TLF was reported (Kaplan–Meier estimate 0.9% [95%CI: 0.1–6.2]), consisting of one CD-TLR on post-procedure day 166. The patient was asymptomatic, but at follow-up angiography, a 63% diameter stenosis (by core laboratory assessment) with an IFR of 0.51 was detected that was successfully treated with an Orsiro DES (BIOTRONIK AG, Buelach, Switzerland). Two patients experienced a CD-TVR (1.9% [95%CI: 0.5–7.2], in addition to the CD-TLR, one CD-TVR occurred on day 177). No myocardial infarction, non-clinically driven TLR or TVR, or definite or probable scaffold thrombosis were reported. Target-vessel myocardial infarction according to the different definitions is provided in Supplemental Table S9.

## Discussion

Our study shows that the third generation metallic DREAMS 3G improves angiographic performance parameters compared to its precursor versions while maintaining a favorable safety profile.

The first marketed scaffold had a polymeric backbone but was withdrawn from the market owing to disappointing outcomes in terms of device thrombosis and adverse events. However, the topic is still alive with DREAMS 2G as marketed metallic scaffold, with a large body of evidence,^7^^,^^17^^,^^18^ and several novel polymeric scaffolds being developed.^19^^,^^20^

DREAMS 3G was built based on continuous improvements on previous scaffold generations. The improved stability over time likely contributed to a reduction in in-scaffold LLL, which improved by 32.3% from DREAMS 1G to DREAMS 2G,^16^^,^^21^ and by 52% from DREAMS 2G to DREAMS 3G (Fig. 2), reaching statistically significant superiority compared to DREAMS 2G and non-inferiority compared to PLLA scaffolds with comparable lesions (Supplemental Table S6).^9^^,^^22^^,^^23^ Further, our mean and median in-scaffold LLL of 0.21 mm (SD 0.31) and 0.13 mm (IQR: 0.05; 0.32), respectively, are both comparable to new generation DES in terms of the objective performance criterion—in-scaffold LLL at nine months—postulated by the European Society of Cardiology–European Association of Percutaneous Cardiovascular Interventions task force (LLL of 0.18 [IQR: 0.13; 0.25 mm]).^10^

In contrast to the relatively higher in-scaffold LLL in BIOSOLVE-II using the precursor device (DREAMS 2G), the neointimal area, measured by IVUS, was already low (0.08 mm^2^), which was attributed to the BIOlute coating that has been successfully used for the Orsiro DES.^21^ The discordance of the low neointimal area and relatively high LLL was speculated to be caused by an extremely early loss of radial strength during the absorption process.^16^ This is likely the case, as in BIOMAG-I with the improved radial strength of DREAMS 3G but the same BIOlute coating, this discordance is no longer present as angiographic LLL and neointimal area are now both low. Importantly, this does not come at a price of uncovered or malapposed struts, that can be a consequence of very low or even negative in-scaffold LLL.^24^ Measured by OCT, only 1.55% (3.10) of struts were malapposed post-procedure, and at six months, struts were already hardly discernible anymore. Similarly, as seen on virtual histology, the white color coding (which is a surrogate of scaffold struts and not necessarily dense calcium) was reduced at six months post-procedure compared to immediately post-procedure, documenting device resorption. Notably, there was a difference in terms of in-device LLL between the imaging modalities, with OCT assessment showing a higher LLL compared to QCA and IVUS. This does not come to a surprise though as previous investigations revealed that there is a poor agreement between QCA, IVUS and OCT.^25^

Related to neointimal coverage, thinner scaffold struts are certainly advantageous for strut embedding and intimal coverage, as thinner struts lead to better vascular healing, as observed with DES.^26^ Furthermore, thinner struts improve scaffold deliverability, along with the increased visibility of radiopaque markers that facilitate scaffold positioning and post-dilatation. Indeed, device success was achieved in all but three devices.

DES with thinner struts are also associated with lower 1-year TLR, periprocedural myocardial infarction, and early stent thrombosis rates, as published in a recent systematic review and meta-regression analysis.^27^ In BIOMAG-I, the safety profile of precursor scaffold versions was maintained with DREAMS 3G^5^^,^^7^^,^^10^^,^^16^; TV-MI and scaffold thrombosis were absent, and only one (0.9%) CD-TLR occurred. Of note, the treated coronary segment had a relatively small reference vessel diameter (2.72 mm [SD 0.46]), and > three quarters of the lesions (76.9%) were AHA/ACC class B2/C. Moreover, BIOMAG-I included 20.7% of the patients with NSTEMI who were excluded from the ABSORB-II, -III, -China, and -Japan trials, as well as from BIOSOLVE-II and BIOSOLVE-III trials, but included in the AIDA, EVERBIO-II, and TROFI-II trials using the Absorb scaffold, and in the BIOSOLVE-IV registry.^7^^,^^8^^,^^28^ Aside of device improvements, another contributing factor to the favorable results could be the adherence to implantation guidelines according to the“4-P-strategy: adequate patient selection, proper sizing, adequate pre-dilatation and adequate post-dilatation.^11^ Notably, a core laboratory-supported analysis of the BIOSOLVE trials reported that improper sizing and poor lesion preparation appeared to be related to increased TLF at follow-up.^29^

Based on the promising outcomes of BIOMAG-I, DREAMS 3G will be compared with contemporary DES in a randomized controlled trial (BIOMAG-II, ClinicalTrials.gov NCT05540223).

The present study has limitations inherent to a non-randomized, first-in-human study. The absence of direct comparison with other permanent stents limits the broad application of our results. Inherent to the nature of a first-in-human study, BIOMAG-I included less complex angiographic lesions and patient characteristics, excluding several frequent and clinically relevant anatomic subgroups. Nevertheless, in contrast to BIOSOLVE-II and BIOSOLVE-III, both of which investigated the precursor (DREAMS 2G), BIOMAG-I permitted inclusion of patients with NSTEMI.

An evaluation of imaging parameters by device diameter would have been interesting as the different device diameters have different strut thicknesses, however, the subgroup sample sizes were too small to create meaningful outcomes. Further studies with larger sample sizes will be able to address this question.

The ideal follow-up time for scaffolds remains unclear, and the time point for imaging analysis at six months remains debatable. However, the time point chosen was in alignment with that in previously reported studies and the literature on DES that mostly reports angiographic LLL at six or nine months. Considering that DREAMS 2G had a similar resorption period and did not show relevant complication rates beyond six months,^8^^,^^17^^,^^30^ this report provides robust insight into the safety and performance of DREAMS 3G. The final confirmation of the ability of DREAMS 3G to reduce long-term adverse events will be obtained from follow-up data.

In conclusion, initial results from the BIOMAG-I first-in-human trial, with nearly complete imaging and clinical follow-up data, showed that the third-generation drug-eluting resorbable magnesium scaffold DREAMS 3G is highly deliverable, exhibits good strut apposition, and has favorable safety and performance outcomes. The excellent safety profile of the previous generations was maintained in DREAMS 3G with low TLF rates, while angiographic LLL was markedly improved, making DREAMS 3G a potential alternative to permanent DES, avoiding lifelong metallic implants associated with adverse long-term outcomes.

## Contributors

MH: per ICMJE terms: conception and design of the work, acquisition, analysis, or interpretation of data for the work, drafting the work and revising it critically, final approval of the version to be published, agreement to be accountable for all aspects of the work in ensuring that questions related to the accuracy or integrity of any part of the work are appropriately investigated and resolved. Per CRediT terms: conceptualization, investigation, methodology, project administration, resources, supervision, writing- original draft, writing-review and editing.

MH and RW directly accessed and verified the underlying data of this manuscript.

AW, JvS, JT, BF, JFI, JB, GT, MJ, RT, MW, GO, PV: acquisition and interpretation of data, critically revising the work, final approval of the version to be published, agreement to be accountable for all aspects of the work. Per CRediT terms: investigation, resource, supervision, writing-review&editing.

HMGG, RW: analysis and interpretation of data, critically revising the work, final approval of the version to be published, agreement to be accountable for all aspects of the work. Per CRediT terms: investigation, resource, supervision, writing-review&editing, validation, project administration, visualization.

## Data sharing statement

Beginning 3 months and ending 5 years following article publication, researchers who provide a methodologically sound proposal will gain access to data that underlie the results reported in this article. Proposals should be directed to stephanie.sauter@biotronik.com to gain access, data requestors will need to sign a data access agreement. Individual patient data will not be shared.

## Declaration of interests

MH reports grants/contracts from Biotronik, Cardiac Dimensions, Orbus Neich and Philips, consulting fees from Biotronik, Cardiac Dimensions, and Obrus Neich, honoraria/speaker fees from Biotronik, Cardiac Dimensions, Orbus Neich and Philips, support to attend meetings/travel support from Biotronik, is a steering committee member of the BIOSOLVE and BIOMAG trials, and is a past president of EAPCI. JT reports speaker honoraria and support for attending meetings from Biotronik, and is an associated editor of Cardiovascular Biologics and Regenerative Medicine, JE reports speaker honoraria from Abbott, Boston Scientific, Philips and Shockwave and participation in advisory boards of Abbott, Phillips and Shockwave, the institution of JI receives grants or contracts from Terumo Corp, Biosensors, Concept Medical, Biotronik, Abbott Vascular, Philips Volcano, JI reports consulting fees from Biotronik, Medtronic, Cordis, and Terumo Corp and speaker fees from Terumo Corp, Biosensors, Medalliance, Orbus Neich, Concept Medical, Bristol Myers Squibb/Pfizer, Novartis, Cordis, AstraZeneca, and Philips Volcano, and support to attend meetings from Biotronik and Amgen, the institution of JB receives grants or contracts from Shockwave IVLS, receives consulting fees from Biotronik AG and Boston Scientific, speaker fees from Biotronik AG, Boston Scientific and Abbott Vascular, JB participates in the DSMB of Boston Scientific and has a leadership or fiduciary role for Biotronik, MJ reports grant support from Boston Scientific, Cardiac Dimensions, Edwards LifeSciences and Infraredx, consulting fees from Biotronik, TriCares, Veryan and Shockwave, speaker fees/honoraria from Abbott Vascular, Biotronik, Boston Scientific, Edwards LifeSciences, Cardiac Dimensions, AstraZeneca, Recor Medical, and Shockwave, travel support from SIS Medical, Edwards Lifesciences, Boston Scientific and Cardiac Dimensions, and participation in Steering Committees of Biotronik and Edwards Lifesciences, MW reports speaker honoraria and conference attendance support from Biotronik, GO reports lecturer honoraria from Abbott Vascular, Biotronik and Cordis and is a DSMB member of the SCIENCE trial and a CEC-member of the BIOFREEDOM STEMI trial, HGG has grants or contracts from Medtronic, Biotronik, Abbott, Neovasc, Corflow, Alucentbio, Philips, Chiesi (paid to institution), received consulting fees from Boston Scientific and ACIST, participates in DSMB/advisory board of the VIVID study, RW has grants or contracts from Astra Zeneca, Biotronik, Boston Scientific, Chiesi, Medtronic, and Philips IGT, and received consulting fees from Abbott Vascular, Biotronik, Boston Scientific, Cordis, Medtronic, Philips IGT, Pi. Cardia Ltd, Swiss Interventional Systems/SIS Medical AG, Transmural Systems Inc, andVenous MedTech, receives honoraria from AstraZeneca, participates in DSMB/advisory boards of Abbott Vascular, Boston Scientific, Medtronic, Philips IGT, and Pi-Cardia Ltd, and is an investor from MedAlliance and Transmural Systems Inc. All other authors have no conflict of interest to declare.

## Appendix A Supplementary data

Clinical Investigation Plan
