# Supplementary material for: Safety and performance of the third-generation drug-eluting resorbable coronary magnesium scaffold system in the treatment of subjects with de novo coronary artery lesions: 6-month results of the prospective, multicenter BIOMAG-I first-in-human study
Source: eClinicalMedicine. 2023 Apr 17;59:101940. doi: 10.1016/j.eclinm.2023.101940 (PMC10126775; doi:10.1016/j.eclinm.2023.101940)
Supplement: Clinical Investigation Plan [file mmc1.pdf]

Clinical Investigation Plan

BIOTRONIK – Safety and Clinical Performance of the Sirolimus-Eluting Resorbable Coronary Magnesium Scaffold System (DREAMS 3G) in the Treatment of Subjects with de Novo Lesions in Native Coronary Arteries: **BIOMAG-I**

Study No: C1702

Version: 3.0 16Apr2021

Version 1.0	22Oct2019
Version 1.1	17Dec2019
Version 1.2	06Jan2020
Version 1.3	20Jan2020
Version 1.4	18Feb2020
Version 2.0	29Apr2020
Version 3.0	16Apr2021

BIOTRONIK AG
Vascular Intervention
Ackerstrasse 6
8180 Bülach / SWITZERLAND

Notice: The information contained in this document is CONFIDENTIAL and PROPRIETARY to BIOTRONIK, and should not be disclosed to anyone who is not a recipient or reviewer of this document

Revision History

Version #	Date	Coutry submitted	Description of changes	Reason for change
1.0	22Oct2019	Germany, Spain, Switzerland, Sweden, Belgium, Netherlands, Poland, Austria	Initial version	Not appl.
1.1	17Dec2019	Spain, Germany	**Section 9.1:** specification added with regards to local safety reporting and reference to German procedure: "Verfahren schwerwiegende unerwünschte Ereignisse in Deutschland" dated 06Dec2019	Clarification of country specific safety reporting requirements
1.1	17Dec2019	Spain, Germany	**Section 5.12.2 and 5.12.3**: ECG as follow procedure was added for the 6 and 12 months follow up visit	ECG as follow up procedure after a re-angiography was added based on hospital's standard of care
			Synopsis and Section 4.9:<br>-Inclusion criteria n°	Inclusion criteria updated to be more specific on

Version #	Date	Coutry submitted	Description of changes	Reason for change
			3 changed as follow: subject eligible for PCI, according to the 2018 ESC/EACTS Guidelines on myocardial revascularization.	subject selection.
1.2	06 Jan 2020	Sweden	Synopsis and Section 4.9:<br>Inclusion criteria n° 8 changed to: Subjects with stable or unstable angina pectoris or documented silent ischemia or hemodynamically stable NSTEMI patients without angiographic evidence of thrombus at target lesion<br>NOTE: patient with acute STEMI can not be included in the study (according to exclusion criteria 2)	A note is added to the inclusion criteria for a better understanding of the exclusion of acute STEMI patients.
1.2	06 Jan 2020	Sweden	Synopsis and Section 4.9:<br>Inclusion criteria n°9 changed to: patient who has no contraindication for DAPT.	Inclusion criteria is re-worded to avoid any miss-understanding.

Version #	Date	Coutry submitted	Description of changes	Reason for change
1.2	06 Jan 2020	Sweden	Synopsis and Section 4.10: Exclusion criteria n° 2 changed to: Subject has clinical symptoms and electrocardiogram (ECG) changes consistent with acute ST elevation myocardial infarction (STEMI) within 72 hours prior to the index procedure. NOTE: after 72 hours, any lesion other than the one causing the acute STEMI (culprit lesion) in any other epicardial vessel, may be treated according to the inclusion and exclusion criteria	Exclusion criteria modified to be in line with inclusion criteria 8 on eligibility definition of STEMI and NSTEMI patients.
1.2	06 Jan 2020	Sweden	**Synopsis and Section 4.10**: Exclusion criteria n°4 changed to: Three-vessels with coronary artery disease requiring treatment at time of procedure, including: left main, left anterior descending artery (LAD) right coronary artery (RCA) and	Corornary artery vessels definitions added to the exclusion criteria for better clarity.

Version #	Date	Coutry submitted	Description of changes	Reason for change
			circumflex coronary artery (Cx)	
1.2	06 Jan 2020	Sweden	**Synopsis and Section 4.10**: Exclusion criteria n°7 changed to: Planned future intervention of a second lesion within the target vessel.	Criteria revised to better explain that only 2 separate vessels with signle target lesions each are allowed in the study.
1.2	06 Jan 2020	Sweden	**Synopsis and Section 4.10**: Exclusion criteria 11 was re-phrased as following: Heavily calcified lesions which can not be adequately pre-dilated by a non-compliant and/ or scoring balloon as described in exclusion criteria 13.	A heavily calcified lesion is more specifically defined in order to avoid any interpretation.
1.2	06 Jan 2020	Sweden	**Synopsis and Section 4.10**: Exclusion criteria n°15 changed to: known allergies or intolerances to: Acetylsalicylic Acid (ASA), P2Y12 inhibitors, Heparin, Contrast medium, Sirolimus, or similar drugs; or the scaffold material (Magnesium,	“Intolerance” is added to this criteria as in some cases, it is not a true allergy but can be another form of reaction.

Version #	Date	Coutry submitted	Description of changes	Reason for change
			Aluminium).	
1.2	06 Jan 2020	Sweden	**Synopsis and Section 4.10**: Exclusion criterion n°17 is deleted .	Criteria deleted as this condition is already covered by exclusion criteria 7.
1.2	06 Jan 2020	Sweden	**Section 6-Adverse Events** of the study protocol has been updated as follow: According to MEDDEV 2.7/3 revision 3, May 2015, the Sponsor shall: Report to the NCAs where the clinical investigation has commenced: - for all reportable events as described in MEDDEV 2.7/3 revision 3, section 4 which indicate an imminent risk of death, serious injury, or serious illness and that requires prompt remedial action for other patients/subjects, users or other persons or a new finding to it: immediately, but not	The timelines for the Sponsor to report events to the Competent Authorites are added according to the MEDDEV 2.7/3 revision 3, May 2015. This is described in the section 7-Reporting timelines, of the MEDDEV and has been added to the protocol.

Version #	Date	Coutry submitted	Description of changes	Reason for change
			later than 2 calendar days after awareness by sponsor of a new reportable event or of new information in relation with an already reported event.<br>- any other reportable events as described in MEDDEV 2.7/3 revision 3, section 4 or a new finding/update to it: immediately, but not later than 7 calendar days following the date of awareness by the sponsor of the new reportable event or of new information in relation with an already reported event.	
1.2	06 Jan 2020	Sweden	**Section 8.5-Data Management** has been updated as follow:<br>The data manager is responsible for setting up an EDC system, its validation and maintenance during study duration. Queries (manual or	The data management section is updated to be more specific on data management responsibilities.

Version #	Date	Coutry submitted	Description of changes	Reason for change
			pre-defined checks) are set up in the EDC system and should be resolved by the investigator or a person designated by the investigator in a timely manner. The data manger provides a clean data set at the end of the clinical investigation. Data snapshots will be performed for interim analysis. When all data is complete, the database will be locked and data analyzed.	
1.2	06 Jan 2020	Sweden	Section 8.7- Audits / Inspections is updated to:<br>BIOTRONIK will evaluate any non-compliance and issue corrective actions, discontinue enrolment or at last measure, close the clinical investigation site, if monitoring or auditing identifies serious or repeated deviations on the part of an investigator.	Possible criteria for the Sponsor to disqualify a study site is added for better definition.

Version #	Date	Coutry submitted	Description of changes	Reason for change
1.3	20Jan2020	Germany	Additional exclusion criteria added in synopsis and section 4.9	The criteria was added in order to exclude patients with impaired renal function
1.3	20Jan2020	Germany	The hypothesis are displayed with mathematical symbols in section 7.2.	The mathematical background was added in order to better clarify the hypothesis that will tested.
1.3	20Jan2020	Germany	The record retention period was prolonged to 10 years in section 9.6.	Based on regulations the minimum record retention period was updated.
1.4	18Feb2020	Switzerland	Section 3.1: the sentence was adapted “subjects may experience adverse events and/or outcomes that are listed in the IFU and are not expected to differ for other contemporary drug eluting stent implantation procesure	The statement was updated in order to include the uncertainty how the device will perform in this first in men study.
1.4	18Feb2020	Switzerland	A table was added listing the potential adverse events that are associated with PTCA, stent and scaffold placement	This table was added in order to give the investigators a better overview of event frequencies

Version #	Date	Coutry submitted	Description of changes	Reason for change
				and to support the monitoring of events.
1.4	18Feb2020	Switzerland	The sentence was updated in section 3.2 : "In addition, the faster resorption of Magmaris and DREAMS 3G (1 year) compared to ABSORB (3-4 years) is expected to reduce the risk of late events (1)"	The statement was updated in order to include the uncertainty how the device will perform in this first in men study.
1.4	18Feb2020	Switzerland	Section 5.4 was updated and the section about legal representative was deleted:<br><br>"The subject must be given ample time to read the subject information and to address questions before signing the consent form. The subject information and a copy of the EC approved signed consent form (by investigator and subject) will be handed out to the subject."	Only subjects that have due capacity and do not need a legal representative are enrolled in this study.

Version #	Date	Coutry submitted	Description of changes	Reason for change
1.4	18Feb2020	Switzerland	Section 5.11. was updated with the information that a patient implantation card will given to every patient prior to discharge.	The patient implantation card is an additional safety measure that should be used. Therefore the instructions about how and when it should be used were added to the CIP.
1.4	18Feb2020	Switzerland	Section 6.1 was updated with the definition of serious device deficiencies : Serious device deficiencies are device deficiencies that turned out to be serious because<br>• suitable action had not been taken;<br>• intervention had not been made; or<br>• if circumstances had been less fortunate.	Serious device deficiencies are reportable events and the definition was listed separately.
1.4	18Feb2020	Switzerland	Section 6.1: a sentence was added that devices should be returned to the sponsor in case of device deficiencies	It is important that devices are returned to the sponsor for further assessment.

Version #	Date	Coutry submitted	Description of changes	Reason for change
1.4	18Feb2020	Switzerland	Section 8.4 in the CIP was updated with the source documents required for device deficiencies.	Serious device deficiencies are reportable and therefore source documents are needed in order to assess these events properly.
1.4	18Feb2020	Switzerland	Section 8.6 was amended with an updated reporting time of 2 days.	According to Article 37 of the clinical ordinance in Switzerland, reporting time is 2 days.
1.4	18Feb2020	Switzerland	Section 9.1 was updated with the reference to a separate document about safety reporting procedures in Switzerland dated 10Jan2020	Safety reporting procedures can be country-specific, therefore the details were summarized in a separate document.
2.0	29Apr2020	Germany, Spain, Switzerland, Sweden, Belgium, Netherlands, Poland, Austria	All changes as listed above implemented	Alignement of all previous approved version on one CIP version
2.0	29Apr2020	Germany, Spain, Switzerland, Sweden, Belgium,	Clarification of Target Lesion Failure and Cardiac Death Endpoints (synopsis and section 4.8)	It was specified that cardiac death will be classified according to ARC-1 definition and

Version #	Date	Coutry submitted	Description of changes	Reason for change
		Netherlands, Poland, Austria		Target Lesion Failure will be classified according to ARC-1 and ARC-2 definition
2.0	29Apr2020	Germany, Spain, Switzerland, Sweden, Belgium, Netherlands, Poland, Austria	Enrolment time was prolonged to 15 months (synopsis and section 4.6)	The enrolment time was prolonged because of the pending situation with regards to the further development of the COVID-19 pandemic.
2.0	29Apr2020	Germany, Spain, Switzerland, Sweden, Belgium, Netherlands, Poland, Austria	Addition of ARC-1 death definition in Appendix 2	Death will be classified according to ARC-1 definition.
2.0	29Apr2020	Germany, Spain, Switzerland, Sweden, Belgium, Netherlands, Poland, Austria	Appendix 2: Definition of Target lesion failure according to ARC-2 was added and it was specified that the existing definition was based on ARC-1	Target Lesion Failure will be classified according to ARC-1 and ARC-2.
2.0	29Apr2020	Germany, Spain, Switzerland,	Correction of typo in Appendix 3	DREAMS 3G should not be implanted in vessels with a

Version #	Date	Coutry submitted	Description of changes	Reason for change
		Sweden, Belgium, Netherlands, Poland, Austria		diameter between <2.5 or >4.2 mm
3.0	19Mar2021	Germany, Spain, Switzerland, Sweden, Belgium, Netherlands, Poland, Austria	Change on the signature page sponsor side	There was a change in the responsible statistician and the contact details were updated.
3.0	19Mar2021	Germany, Spain, Switzerland, Sweden, Belgium, Netherlands, Poland, Austria	Addition of an additional exclusion criteria (# 19) in the synopsis and in section 4.10	Clarifiation of medication therapy because patients on triple anti-platelet therapy may have an increased bleeding risk and therefore DAPT would potentially have to be discontinued which is a protocol violation.
3.0	19Mar2021	Germany, Spain, Switzerland, Sweden, Belgium, Netherlands, Poland, Austria	In section 1.1.3, the information was updated for the BIOSOLVE IV study.	Analysis of the first 1075 patients of BIOSOLVE IV up to 12 months was published and the background information was added.
3.0	19Mar2021	Germany, Spain, Switzerland, Sweden,	The sentence about temperature-controlled shipments in section 2.10 was	More test results from thermal cycling testing are available and no temperature

Version #	Date	Coutry submitted	Description of changes	Reason for change
		Belgium, Netherlands, Poland, Austria	deleted.	controlled shipments are needed.
3.0	19Mar2021	Germany, Spain, Switzerland, Sweden, Belgium, Netherlands, Poland, Austria	Recommendation about Oral Anticoagulation Therapy was added to section 5.7.	Alignment of new exclusion criteria with medication guideline section 5.7.
3.0	19Mar2021	Germany, Spain, Switzerland, Sweden, Belgium, Netherlands, Poland, Austria	OCT assessment pre-procedure was added in section 5.8	OCT pre-procedure will allow a better assessment of healing process of the lesion that gets treated with the DREAMS 3G device (original lesion compared to after treatment compared to follow up time points).
3.0	19Mar2021	Germany, Spain, Switzerland, Sweden, Belgium, Netherlands, Poland, Austria	Chronic disease was added to the serious criteria in section 6.1	The definition was adapted to fit the updated version 2020 of ISO14155.

Protocol Signature Page – Sponsor

Prepared by:

**Dr. Stephanie Sauter**<br>Senior Clinical Project Manager<br><br>BIOTRONIK AG<br>Ackerstrasse 6<br>8180 Buelach, SWITZERLAND<br>Phone.:+41 44 864 55 75<br>Fax: +41 44 864 5480<br>Email: stephanie.sauter@biotronik.com	21 Apr 2021 [illegible]<br>Date, Signature

Approved by:

**Myriam Stieler**<br>Director Medical Affairs<br>Heart Disease<br><br>BIOTRONIK AG<br>Ackerstrasse 6<br>8180 Buelach, SWITZERLAND<br>Phone.:+41 44 864 5517<br>Fax: +41 44 864 5480<br>Email: myriam.stieler@biotronik.com	22 Apr 2021 [illegible]<br>Date, Signature
**Marcel Schaefer**<br>Senior Director Regulatory Affairs and PMS<br><br>BIOTRONIK AG<br>Ackerstrasse 6<br>8180 Buelach, SWITZERLAND<br>Phone.:+41 44 864 5520<br>Fax: +41 44 864 5480<br>Email: marcel.schaefer@biotronik.com	[illegible] Digitally signed by Marcel Schäfer<br>Date: 2021.04.26 09:22:10 +02'00'<br>Date, Signature

**Christian Knoll**<br>Senior Statistician<br><br>BIOTRONIK AG<br>Ackerstrasse 6<br>8180 Bülach, SWITZERLAND<br>+41 44 864 5684Fax: +41 44 864 5480<br>Email: christian.knoll@biotronik.com	21 Apr. 2021 Chr. Knoll<br>Date, Signature
**Klaus Schichl**<br>Director Clinical Affairs CRM/EP<br><br>On behalf of<br>Authorized Sponsor Representative<br><br>BIOTRONIK SE & Co. KG<br>Woermannkehre 1<br>12359 Berlin, GERMANY<br>Phone:+49 30 689 05 12 30<br>Fax: +49 30 689 05 96 12 30<br>Email: klaus.schichl@biotronik.com	K. Schichl<br>23 APR 2021<br>Date, Signature

Protocol Signature Page - Coordinating Clinical Investigator

I have read this CIP and agree to adhere to the requirements described in this study protocol, to local legal and regulatory requirements and the current versions of the Declaration of Helsinki (Fortaleza version 2013, active at protocol signature) ICH/GCP guidelines (version 2002 active at protocol signature), ISO 14155 (version 2011, active at protocol signature), Medical Devices Directive (93/42/EEC), (active at protocol signature) and to local IRB/IEC regulations.

I will provide copies of this study protocol and all necessary information about this study to the study staff under my supervision.

I will discuss this material with them and ensure they are fully informed about the device under investigation as well as all aspects concerning the conduct of this study.

I will supervise the conduct of the clinical investigation to be performed in compliance with the clinical investigational plan and all applicable legal and regulatory requirements.

**Prof. Dr. Michael Haude**<br>Rheinland Klinikum Neuss GmbH<br>Lukaskrankenhaus<br>Preussenstrasse 84<br>41464 Neuss<br>Germany	27.4.21 [signature]<br>Date, Signature

Protocol Signature Page – Investigator

I have read this CIP and agree to adhere to the requirements described in this study protocol, to local legal and regulatory requirements and the current versions of the Declaration of Helsinki (Fortaleza version 2013, active at protocol signature) ICH/GCP guidelines (version 2002 active at protocol signature), ISO 14155 (version 2011, active at protocol signature), Medical Devices Directive (93/42/EEC), (active at protocol signature) and to local IRB/IEC regulations.

I will provide copies of this study protocol and all necessary information about this study to the study staff under my supervision.

I will discuss this material with them and ensure they are fully informed about the device under investigation as well as all aspects concerning the conduct of this study.

Investigator

Name: ____

Institution: ____

Address: ____

ZIP / Place: ____

Phone: ____

Fax: ____

Email: ____

Date / Signature: ____

TABLE OF CONTENTS

PROTOCOL SYNOPSIS **24**
STUDY ASSESSMENT OVERVIEW **31**
PRIMARY CONTACTS **33**
SPONSORING AND AGREEMENT **33**
ROLES, RESPONSIBILITIES & QUALIFICATION OF INVESTIGATOR TYPES **34**
1. INTRODUCTION **35**
1.1 Prior BIOTRONIK Bioresorbable Vascular Scaffold (BVS) Investigations **37**
1.2 Other Manufacturers‘ Prior Investigations **49**
1.3 DREAMS 3G and BIOMAG-I **52**
1.4 Treatment recommendations **53**
2. INVESTIGATIONAL DEVICE **54**
2.1 Manufacturer of the device 54
2.2 Intended purpose 54
2.3 Device Description 54
2.4 Use of the device(s) during the investigation 54
2.5 Necessary training / experience for the use of the investigational device 55
2.6 Size Matrix 55
2.7 Preclinical Testing 55
2.8 Current state of the art in clinical care in the relevant field of application 55
2.9 Device Supply/ Device Labeling 56
2.10 Device Storage 56
2.11 Device traceability 56
2.12 Device Accountability 56
2.13 Recall of investigational devices 57
3. RISK-BENEFIT-ANALYSIS **57**
3.1 Potential Risks 57
3.2 Potential Benefits 61
3.3 Risk / Benefit Conclusion 65
4. CLINICAL INVESTIGATION **66**
4.1 Study Objectives 66
4.2 Hypothesis 66
4.3 Relevance of the trial in the context to the state of the art of clinical practice 67
4.4 Study Design 67
4.5 Study population 68

4.6 Study Duration ... 68
4.7 Primary Endpoint ... 68
4.8 Secondary Endpoints ... 68
4.9 Inclusion Criteria ... 70
4.10 Exclusion Criteria ... 70
5. CLINICAL INVESTIGATION PROCEDURES ... 73
5.1 Study Participation Status ... 73
5.2 Enrollment and Procedures ... 74
5.3 Pre-screening ... 75
5.4 Informed Consent Process ... 75
5.5 Enrollment ... 76
5.6 Baseline Examinations ... 76
5.7 Medication guidelines ... 77
5.8 Baseline Angiography ... 78
5.9 Index Procedure ... 79
5.10 Treatment Failure and Bailout Situation ... 80
5.11 Post Procedure until Discharge ... 81
5.12 Follow-Up Visits ... 82
5.13 Withdrawals / Study Exit ... 87
5.14 Study completion/Termination ... 87
6. ADVERSE EVENTS ... 87
6.1 Adverse Event Definition and Classification ... 87
7. STATISTICAL ANALYSIS ... 92
7.1 Study Populations ... 92
7.2 Determination of Sample Size ... 93
7.3 Analysis Methods ... 96
7.4 Missing Data ... 97
7.5 Interim Analysis and Final Report ... 97
7.6 Subgroup analysis ... 97
8. QUALITY CONTROL AND QUALITY ASSURANCE ... 97
8.1 Data Monitoring Committee ... 97
8.2 Core Laboratory ... 98
8.4 Source Data ... 100
8.5 Data Management ... 101
8.6 Protocol Compliance ... 101
8.7 Audits / Inspections ... 103
9. REGULATORY REQUIREMENTS ... 103

9.1 Compliance Statement 103
9.2 IRB/IEC and Regulatory Bodies 103
9.3 Insurance 104
9.4 Subject data protection 104
9.5 Records 104
9.6 Record Retention 105
9.7 Study Termination 106
9.8 Investigator Reimbursement and Contracting 107
10. PUBLICATION POLICY 107
11. APPENDICES 109
APPENDIX 1: Abbreviations 110
APPENDIX 2: Definitions 113
APPENDIX 3: The 4P Strategy 139
12. References 139

<u>The following terms will be used synonymously:</u>

Clinical Investigation Plan (CIP) = Clinical Protocol = Study Plan

Clinical Investigation = (clinical) Study = (clinical) Trial

CRF = eCRF = EDC

DREAMS 2G = DREAMS 2nd generation = Magmaris (trade name)

Mg = Magnesium

Protocol Synopsis

**Title**	**BIO**TRONIK – Safety and Clinical Performance of the Sirolimus-Eluting Resorbable Coronary Magnesium Scaffold System (DREAMS 3G) in the Treatment of Subjects with de Novo Lesions in Native Coronary Arteries: BIOMAG-I
**Indication**	Subjects with de novo coronary artery lesions: Subjects with symptomatic coronary artery disease who qualify for percutaneous coronary intervention (PCI).
**Design**	A prospective, multi-center, first-in-man trial.<br>Up to 115 subjects will be enrolled.<br>Clinical follow-up visits will take place at 1, 6, and 12 months and annually thereafter until 36 months post procedure.<br>All subjects will undergo an angiographic follow-up at 6- and 12-month follow up.<br>IVUS, (including IVUS-VH documentation) and OCT will be performed for all subjects at 6-month and 12-month follow-up (if the safety of the subject allows it and as per the investigator’s decision).<br>Vasomotion will be assessed angiographically with Acetylcholine followed by Nitroglycerine at 12 months follow up in a subgroup of subjects, upon the investigators discretion and if subject consents.
**Objectives**	Assessment of safety and clinical performance of the DREAMS 3G in de novo coronary artery lesion in order to achieve and to obtain CE-approval
**Investigational Devices**	The Dreams 3G will be available in the following sizes:

Scaffold length (mm)

Scaffold diameter (mm)	13	22	30
2.5	x	x	
3	x	x	x
3.5	x	x	x
4	x	x	

Primary Endpoints

The primary endpoint will be in-scaffold late lumen loss (LLL) at 6-month post-procedure.

Secondary Endpoints

Clinical

- Target Lesion Failure (TLF*) at 1, 6, 12 months and annually thereafter until 36 months post procedure
- Cardiac death at 1, 6, 12 months and annually thereafter until 36 months post procedure (according to ARC-1 definition)
- Target vessel MI at 1, 6, 12 months and annually thereafter until 36 months post procedure**
- Clinically driven target lesion revascularization at 1, 6, 12 months and annually thereafter until 36 months post procedure
- Clinically driven target vessel revascularization at 1, 6, 12 months and annually thereafter until 36 months post procedure
- Definite and probable scaffold thrombosis rate at 1, 6, 12 months and annually thereafter until 36 months post procedure (according to ARC-2 definition)

	▪ Procedure success: achievement of a final diameter stenosis of <30% by QCA, using any percutaneous method, without the occurrence of death, Q-wave or non-Q-wave MI, or repeat revascularization of the target lesion during the hospital stay.<br>▪ Device Success: final residual diameter stenosis of <30% by QCA, or visual assessment using the assigned device only with:<br>— Successful delivery of the scaffold to the target lesion, and<br>— Appropriate scaffold deployment, and<br>— Successful removal of the delivery system<br>* defined according to ARC-2 definition and ARC-1 definition<br>**periprocedural MIs will be adjudicated according to SCAI-definitions and ARC-2 definition<br>**Angiographic**<br>▪ In-segment late lumen loss at 6 months<br>▪ In-scaffold and in-segment late lumen loss at 12 months<br>▪ Binary in-scaffold and in-segment restenosis rate at 6 and 12 months<br>▪ % in-scaffold and in-segment diameter stenosis at 6 and 12 months<br>**OCT and IVUS**<br>Descriptive analysis of vessel morphology, lesion composition and scaffold strut data<br>**Vasomotion**<br>Descriptive analysis of vessel movement
**Inclusion**	1. Subject is ≥ 18 years and ≤ 80 years of age

**Criteria**	2. Written subject informed consent available prior to PCI<br>3. Subject eligible for PCI, according to the 2018 ESC/EACTS Guidelines on myocardial revascularization.<br>4. Subjects with a maximum of two single lesions in two separate coronary arteries which have to be de novo lesions and can be covered with 1 device each<br>5. Reference vessel diameter between 2.5-4.2 mm by visual estimation, depending on the scaffold size used<br>6. Target lesion length ≤ 28 mm by visual estimation, depending on the scaffold size used<br>7. Target lesion stenosis by visual estimation ≥ 50% - < 100% and TIMI flow ≥1 (assisted by e.g. QCA / IVUS /FFR).<br>8. Subjects with stable or unstable angina pectoris or documented silent ischemia or hemodynamically stable NSTEMI patients without angiographic evidence of thrombus at target lesion<br>NOTE: patient with acute STEMI can not be included in the study (according to exclusion criteria 2)<br>9. Subject who has no contraindication for.Dual Anti Platelet Therapy (DAPT)
**Exclusion Criteria**	1. Pregnant or breast-feeding females or females who intend to become pregnant during the time of the study<br>2. Subject has clinical symptoms and electrocardiogram (ECG) changes consistent with acute ST elevation myocardial infarction (STEMI) within 72 hours prior to the index procedure.<br>NOTE: after 72 hours, any lesion other than the one causing the acute STEMI (culprit lesion) in any other epicardial vessel, may be treated according to the

	inclusion and exclusion criteria<br>3. Left main coronary artery disease<br>4. Three-vessels with coronary artery disease requiring treatment at time of procedure, including: left main, left anterior descending artery (LAD) right coronary artery (RCA) and circumflex coronary artery (Cx)<br>5. Planned interventional treatment of any non-target vessel within 12-month post-procedure<br>6. Subjects on dialysis<br>7. Impaired renal function (serum creatinine > 2.5 mg/dl or 221 µmol/l, determined within 72 hours prior to intervention)<br>8. Planned future intervention of a second lesion within the target vessel.<br>9. Ostial target lesion (within 5.0 mm of vessel origin)<br>10. Target lesion involves a side branch >2.0 mm in diameter<br>11. Documented left ventricular ejection fraction (LVEF) ≤ 30% within the last 6 months<br>12. Heavily calcified lesion which can not be adequately pre-dilated by a non-compliant and/ or scoring balloon as described in exclusion criteria 15.<br>13. Target lesion is located in or supplied by an arterial or venous bypass graft<br>14. Target lesion requiring treatment with a device other than the non-compliant pre-dilatation balloon or scoring balloon prior to scaffold placement (including but not

	limited to rotational atherectomy, etc.)<br><br>15. Unsuccessful pre-dilatation, defined as a residual stenosis rate more than 20%, estimated by any method and/or angiographic complications (e.g. distal embolization, side branch closure, extensive dissections)<br><br>16. Known allergies or intolerances to: Acetylsalicylic Acid (ASA), P2Y12 inhibitors, Heparin, Contrast medium, Sirolimus, or similar drugs; or the scaffold material (Magnesium, Aluminium)<br><br>17. Subject is receiving an oral or intravenous immuno-suppressive therapy (e.g., inhaled steroids are not excluded) or has a known life-limiting immunosuppressive or autoimmune disease (e.g., human immunodeficiency virus, systemic lupus erythematosus) diabetes mellitus is not excluded)<br><br>18. Life expectancy less than 1 year<br><br>19. Subjects under oral anticoagulation therapy (OAC) prior to implantation of DREAMS 3G unless DAPT can be maintained for a minimum of 6-month.<br><br>Recommendation: If a subject requires OAC after DREAMS 3G implantation, DAPT should be maintained until 6 months follow up. Afterwards DAPT can be downsized to either ASA or Clopidogrel alone together with OAC for the remaining time period up to 12 months. After this, OAC monotherapy can be prescribed if still required.<br><br>20. Planned surgery or dental surgical procedure within 6 months after index procedure unless DAPT will be

	maintained<br>21. In the investigators opinion, subject will not be able to comply with the follow-up requirements<br>22. Subject is currently participating in another study with an investigational device or an investigational drug and has not reached the primary endpoint yet
**Study Duration**	The total study duration is approximately 3.5 years, including the enrolment phase of 15 months and the 36 months follow-up period.
**Coordinating Investigator**	Prof. Dr. Michael Haude
**Data Monitoring Committee**	Combination of a Clinical Event Committee and Data Safety Monitoring Board responsible for review and adjudication of clinical events and periodic safety reviews.
**Steering Committee**	Scientific committee responsible for protocol development and oversight of study conduct.
**Angiographic/ IVUS and OCT Core Laboratory**	MedStar Health Research Institute<br>Washington DC, USA

Study assessment overview

Test and procedure	Visit*						
	Screening - 7 d prior to intervention	Procedure	Discharge	30 d FUP ± 7 d	6 M FUP ± 30 d	12 M FUP ± 30 d	24 and 36 M ± 30 d
Subject Information and Informed Consent	✓						
Review of in-/ exclusion criteria	✓	✓					
Demographic assessment and medical history	✓						
Physical examination	✓						
Pregnancy test[a]	✓				✓	✓	
Angina status	✓			✓	✓	✓	✓
Serum Creatinine[b]	✓						
Cardiac biomarkers[1c]	✓		✓				
12-lead[d] electrocardiogram	✓		✓		✓[f]	✓[f]	
Procedural information		✓					
Angiography		✓			✓	✓	
IVUS		✓			✓	✓	
OCT		✓			✓	✓	
Vasomotion[e]						✓[e]	
Concomitant Medication	✓	✓	✓	✓	✓	✓	✓
Adverse Events		✓	✓	✓	✓	✓	✓
Device Deficiencies		✓	✓	✓	✓	✓	✓

[1]Assessment of CK-MB is mandatory.

[a]Women with childbearing potential only, 72 hours before index angiography.

[b] Within 72 hours prior to procedure.

[c] CK, CK-MB and Troponin (cTn (I or T)) within 24 hours of the procedure and 6-24 hours post-procedure respective at discharge, whichever comes first. If elevation is noted post-procedure, CK, CK-MB and Troponin measurements should be repeated every 8 hours until the values have returned to normal.

[d] Within 24 hours of the procedure and 6-24 hours post-procedure respective at discharge, whichever comes first.

[e] Only if the subject consents

[f] According to hospital's standard of care

*Follow-up visits can be conducted by telephone unless an angiographic, IVUS or OCT assessment is scheduled.

Primary Contacts

Sponsor

BIOTRONIK AG
Dr. Stephanie Sauter
Vascular Intervention
Ackerstrasse 6
8180 Buelach
Switzerland
Phone: + 41 44 864 55 75
Email:
stephanie.sauter@biotronik.com

BIOTRONIK AG
Souheila Moutiq
Vascular Intervention
Ackerstrasse 6
8180 Buelach
Switzerland
Phone: +41 44 864 56 25
Email:
souheila.moutiq@biotronik.com

Coordinating Investigator

Prof. Dr. med Michael Haude
Städtische Kliniken-Neuss
Lukaskrankenhaus GmbH
Preussenstrasse 84
41464 Neuss
Germany
Phone:+49 2131 888 2000
Email:mhaude@lukasneuss.de

Participating Sites

A current list of participating centers and a detailed list of contacts (incl. emergency contact details of the PIs) are filed in the Trial Master File at Biotronik.

Angiographic/ IVUS and OCT Core Laboratory:

MedStar Health Research Institute
Hector Garcia Garcia
6525 Belcrest Road, Suite 700,
Hyattsville, MD, 20782
USA

Phone: +1 202 877 77 54
Email: hector.m.garciagarcia@medstar.net

Sponsoring and agreement

The BIOMAG-I study is financed by BIOTRONIK AG.
There is an agreement between BIOTRONIK AG and each site stipulating the responsibilities of each party involved.

Roles, responsibilities & qualification of investigator types

Principle investigator

The responsibilities of and qualification requirements for the principal investigator (PI) are defined according to ISO 14155. The PI is required to be trained and experienced in the use of the investigational device or of comparable devices.

Co-Investigator

A co-investigator is any individual member of the clinical trial team designated and supervised by the PI at a trial site to perform critical trial-related procedures and/or to make important trial-related decisions (e.g. associates, residents, research fellows) according to ICH-GCP E6. All other functions are defined in the site signature and responsibility log.

1. INTRODUCTION

Standard of care treatment of coronary artery disease with state of the art drug-eluting stents (DES) delivers good clinical outcomes with low target lesion failure (TLF) and stent thrombosis rates (2,3). Nevertheless the use of DES presents some limitations, mainly due to the permanent presence of foreign material in the vessel wall. In particular there is a long term risk of stent failure, thrombosis, chronic inflammation due to the metal or polymer components and neoatherosclerosis (4). Moreover the metal cage can impair the vessel geometry, access and flow into side branches and can inhibit a normal vasomotor function, which may hinder compensatory positive re-modelling and limits use of imaging and future treatment options (5,6). Bioresorbable scaffolds (BRS) were developed to overcome these problems, enabling vessel restoration and reducing long term risks. BRS are meant to provide a temporary drug eluting scaffold, which supports the vessel after implantation as long as needed, limiting acute recoil and negative re-modelling, and enable a natural biologic reconstruction of the arterial wall and restoration of the vascular function once the scaffold is resorbed, which may also reduce the need of prolonged dual antiplatelet therapy and the occurrence of related bleeding complications (6,7).

The ability of scaffolds to meet these expectations however have been partly questioned by suboptimal results of the Absorb BVS (Abbott Vascular, Santa Clara, CA) showing higher incidence of scaffold thrombosis and target vessel-related myocardial infarction (8,9). Notably, while ABSORB BVS Bioresorbable Vascular Scaffold (BVS) consists solely of a poly-L-lactic acid (PLLA) polymer which resorbs over a period of more than 24 months, other scaffolds have different materials and designs. In particular, the Magmaris (here after refered to also as DREAMS 2G) (BIOTRONIK AG, Bülach, Switzerland) consist of a magnesium alloy which resorbs in approximately 12 months. Data from clinical trials thus far have demonstrated the clinical safety and performance of

Magmaris and have not shown the same safety concerns as Absorb. DREAMS 2G gained CE-Mark in 2016 and is since then marketed as Magmaris.

Moreover, there are several indicators that Magmaris is less thrombogenic, e.g.

- In 30 patients assessed up to 6 months and 11 up to 12 months, no intraluminal mass was detected by OCT. Furthermore, at 6 months, no malapposed struts were detected because the struts were already embedded in the vessel wall (10,11)
- DREAMS is laser polished, leading to a very smooth surface (10,11)
- The strut cross section is rectangular with rounded edges, which might result in better embedding into the vessel wall (11)
- DREAMS does not require stepwise inflation as required for polymeric scaffolds (12), which may result in better expansion and apposition (11)
- A porcine arterio-venous shunt model compared the acute thrombogenicity of DREAMS 2G, the ultrathin Orsiro DES that uses the same polymer/drug combination as DREAMS 2G, and the ABSORB BVS scaffold. It demonstrated that DREAMS 2G had significantly less (a) platelet adherence, (b) thrombus deposition, and (c) inflammatory cell adhesion than the ABSORB BVS scaffold. Despite a greater strut thickness of DREAMS 2G as compared to Orsiro, the findings were similar in both devices with the exception of significantly less inflammatory cell adhesion in DREAMS 2G (13).
- Similarly, a study in porcine and rabbit models showed an increased endothelialization and decreased thrombus formation for DREAMS 2G compared to Absorb. Inflammation for DREAMS 2G peaked at 90 days and decreased thereafter; at one and two years, inflammation was lower for DREAMS 2G versus an everolimus-eluting cobalt-chromium stent (14).
- In-vitro tests showed an improved deliverability of DREAMS 2G as compared to the ABSORB BVS scaffold due to the metallic properties of DREAMS 2G, with less bending stiffness despite higher radial strength, indicating a better vessel conformability and no time dependent recoil of

DREAMS 2G in contrast to ABSORB BVS and DESolve, a novolimus-eluting bioresorbable coronary scaffold system (15).

1.1 Prior BIOTRONIK Bioresorbable Vascular Scaffold (BVS) Investigations

Table 1 summarizes the clinical trials performed and the iterative design improvements on BIOTRONIK’s magnesium scaffold which have resulted in improved late lumen loss (LLL) and similar target lesion failure (TLF) rates.

Table 1: Overview of Magnesium Scaffold Trials

Study	PROGRESS-AMS	BIOSOLVE-I	BIOSOLVE-II	BIOSOLVE-III	BIOSOLVE-IV
Scaffold Iteration	AMS (bare absorbable metal scaffold)	DREAMS 1G (paclitaxel-eluting PLGA)	Magmaris (DREAMS 2G) (sirolimus-eluting PLLA)	Magmaris (DREAMS 2G) (sirolimus-eluting PLLA)	Magmaris (DREAMS 2G) (sirolimus-eluting PLLA)
Design/ Subjects	Multi-center<br>Single-arm FIH<br>N=63 subjects	Multi-center<br>Single-arm FIH<br>N=43 subjects	Multi-center<br>Single-arm FIH<br>N=123	Multi-center<br>Single-arm pre-market study<br>N=61	Multi-center<br>Single-arm registry (post CE mark)<br>N=1065; Expanded to N=2054
Primary Endpoint/ Key Secondary	MACE	TLF	LLL	In-hospital procedural success	TLF@ 12 months
Results	• LLL: 1.08±0.49 mm @ 4 months<br>• TLF: 26.7%<br>• Definite/probable ST: 0.0% @ 12 months	• LLL: 0.65±0.50 mm @ 6 months<br>• TLF: 7.0%<br>• Definite/probable ST: 0.0% @ 36 months	LLL: 0.44±0.36 mm @ 6 months<br>• TLF: 6.8%<br>• cardiac death: 1.7%<br>• TV-MI: 0.9%<br>• cd-TLR: 4.3%.<br>• Definite/probable ST: 0% @ 36 months	• TLF: 3.3%<br>• cardiac death: 1.6%<br>• TV-MI: 0%<br>• cd-TLR: 1.6%<br>• Definite/probable ST: 0%<br>@ 12 months	• TLF: 4.3% @ 12 months<br>• cardiac death: 0.2%<br>• TV-MI: 1.1%<br>• cd-TLR: 3.9%.<br>Definite/probable ST: 0.5%<br>@ 12 months
Follow-up	12 months	36 months	60 months	36 months	60 months

Study	PROGRESS-AMS	BIOSOLVE-I	BIOSOLVE-II	BIOSOLVE-III	BIOSOLVE-IV
Duration					
Study Status	Enrollment and follow-up complete	Enrollment and follow-up complete	Enrollment complete	Enrollment complete	Enrollment of full cohort complete. 12 months results of first cohort (1075 patients) published.
Reference	(16)	(10)<br>(17)	(18)<br>(19,20)	(19)<br>(21)	(22)

cd-TLR = clinically-driven target lesion revascularization, LLL = in-scaffold late lumen loss, MACE = major adverse cardiac events, ST = scaffold thrombosis, TLF = target lesion failure, TV-MI = target vessel myocardial infarction.

Table 1 ctd.

**Study**	**Magnesium 2000**	**ACS registry**	**Blachutzik et al.**	**STEMI-Pilot**	**MAGSTEMI**
Scaffold Iteration	Magmaris (DREAMS 2G) (sirolimus-eluting PLLA)	Magmaris (DREAMS 2G) (sirolimus-eluting PLLA)	Magmaris (DREAMS 2G) (sirolimus-eluting PLLA)	Magmaris (DREAMS 2G) (sirolimus-eluting PLLA)	Magmaris (DREAMS 2G) (sirolimus-eluting PLLA)
Design/ Subjects	Multi-center Postmarket survey N=2018 subjects	IIT: Prospective single-center registry in ACS patients N=50 subjects	IIT: Single center registry N=35 subjects	IIT: Single center prospective pilot study in STEMI patients N=18 subjects	IIT: multi-center, 1:1 randomized-controlled singleblinded trial in STEMI patients N=150 subjects (N=74 Magmaris, N=76 Orsiro)
Primary Endpoint/ Key Secondary	Procedural characteristics	Clinical findings	OCT and clinical findings	DOCE, a composite of cardiac death, TV-MI and TLR within 30 days	Vasomotion at 12 months
Results	• Successfully deployed in 99%<br>• Performance rated as good or very good in 96%	• TLF at 6 month: 0%<br>• Scaffold thrombosis: 0%	• TLF at 6 months: 6%<br>• TLR at 6 months: 6%<br>• Mortality: 0%<br>• Myocardial infarction: 0%<br>• Scaffold thrombosis: 0%	• DOCE at 30 days: 0%<br>• DOCE at 153 days: 5.6% (n=1 TLR)<br>• Probable or definite scaffold thrombosis: 0%	In-stent/in-scaffold vasodilatory response ≥3% (delta in mean lumen diameter) after intracoronary nitroglycerin injection at 12-month angiographic follow-up with superiority of Magmaris over Orsiro in the as treated

Study	Magnesium 2000	ACS registry	Blachutzik et al.	STEMI-Pilot	MAGSTEMI
					population
Follow-up Duration	Procedure only	6 months	6 months	153 days	Up to 60 monts
Study Status	Completed	Completed	Completed	Completed	Ongoing
Reference	(23)	(24)	(25)	(26)	(27)

ACS = acute coronary syndrome, DOCE = device-oriented composite endpoint, IIT = investigator-initiated trial, MI = myocardial infarction, OCT = optical coherence tomography, STEMI = ST-elevation myocardial infarction, TLR = target lesion revascularization, TV = target vessel

1.1.1 AMS

The first metal scaffold made of an absorbable magnesium (Mg) alloy was the Absorbable Metal Stent (AMS) (BIOTRONIK AG, Bülach, Switzerland). This scaffold had a high mechanical strength, low elastic recoil (<8%), high collapse pressure (0.8 bar), and minimum foreshortening (<5%), all of which are comparable to stainless steel stents (28,29). Furthermore, the resorption of Mg produces an electronegative charge resulting in a hypothrombogenic scaffold. The initial preclinical study in a porcine model showed that the scaffold was rapidly re-endothelialized and degraded into inorganic salts with little inflammatory response (30,31).

The device showed acceptable safety and patency rates in the treatment of infrapopliteal arteries in 20 subjects (32).

<u>PROGRESS AMS Study</u>

In coronary arteries, the AMS device was tested in the PROGRESS-AMS trial (33). This multi-center, single-arm First in Human (FIH) study assessed the efficacy and safety of the scaffold in 63 subjects with single *de novo* lesions. The study showed a good safety profile with no death, myocardial infarction (MI) or scaffold thrombosis during the 12 month follow up. Furthermore, intravascular ultrasound (IVUS) analysis showed the absence of residual metal. However, in-scaffold late lumen loss and ischemia-driven target lesion revascularization (ID-TLR) were high: 1.08±0.49 mm and 23.8%, respectively, at 4 months. IVUS analysis showed that acute recoil was the primary cause of the high late lumen loss and restenosis likely due to early loss of radial force of the scaffold, suggesting the need for slower scaffold absorption and addition of an anti-proliferative drug elution concept to prevent neointimal hyperplasia (16,33,34). In summary, this study showed the safety and feasibility of an AMS, but also a necessity for the modification of some of the scaffold properties.

1.1.2 DREAMS 1G

The AMS device was improved by using a different Mg alloy to prolong the resorption time and to improve the scaffolding properties. In particular, to improve radial strength, the cross section shape of the strut was altered from rectangular to square. In addition, to inhibit neointimal proliferative response, the scaffold strut surface was coated with a drug-polymer matrix of the absorbable polymer carrier polylactic-

coglycolic-acid (PLGA) and the antiproliferative drug Paclitaxel (29,35). This next generation iteration was named the Drug Eluting Absorbable Metal Scaffold (DREAMS) 1G.

BIOSOLVE-I Study

DREAMS 1G was evaluated in the BIOSOLVE-I study (10). In this prospective, multicenter, FIH trial, 46 subjects at five European centers were enrolled. The study results revealed a good clinical safety profile as there was neither cardiac death nor scaffold thrombosis up to the 12-month follow-up. Only one peri-procedural target vessel MI occurred due to the treatment of a stenosis located at the circumflex artery during the 12 month follow-up angiography. As by definition the target vessel also includes side branches, this MI was classified as target vessel MI even though it was not related to the original target lesion located in obtuse marginal artery. Due to this event, the TLF rate increased to 7% at the 1 year follow up while the 6-month result was 4%. In comparison to its AMS precursor, DREAMS demonstrated a significant improvement in 12-month clinically driven TLR (4.7% (10) vs 26.7% (33)). The TLF rate for BIOSOLVE-I remained stable with no further TLF cases up to 3 years and thus the 3-year TLF rate (7%) was comparable or better than the 10% TLF rate in ABSORB Cohort B (36). Moreover no probable or definite stent thrombosis was reported throughout the study.

With an in-scaffold late lumen loss (LLL) of 0.65±0.50 mm at 6 months and 0.52±0.39 mm at 12 months, DREAMS did not reach the excellent late lumen loss of ABSORB BVS scaffold or other contemporary DESs (37–40). However, the in-scaffold LLL at 6 months (0.65±0.50 mm) and at 12 months (0.52±0.39mm) showed a reduction of 40% and 52%, respectively, compared to the LLL of 1.08 ± 0.49 mm at the 4 month follow up reported in the PROGRESS study (33).

In summary, the BIOSOLVE-I study showed that DREAMS 1G was not competitive to ABSORB BVS scaffold or other commercially available DES with respect to late lumen loss, although the refinement of the device resulted in significant improvements compared to the bare AMS and although the TLF rates were comparable to contemporary DESs and the ABSORB BVS scaffold (10).

1.1.3 DREAMS 2G

The next generation of the scaffold, DREAMS 2G, addressed the limitations of DREAMS 1G. The scaffold backbone was modified to obtain a more flexible and stronger device, especially to improve acute and chronic radial strength. Radiopaque markers were added for x-ray visibility of the scaffold. Furthermore, the drug-polymer coating was changed to bioabsorbable Poly-L-Lactic Acid (PLLA) and Sirolimus, BIOlute™ was chosen instead of Paclitaxel to decrease neointimal formation more effectively. The same coating is also successfully used in the Orsiro Sirolimus-eluting coronary stent system that has been commercially available outside the US since 2011 (BIOTRONIK AG, Buelach, CH) and that recently has gained FDA-approval (41–45).

BIOSOLVE-II Study

DREAMS 2G has been investigated in the BIOSOLVE-II study (BIOTRONIK – Safety and Clinical Performance of the Drug Eluting Absorbable Metal Scaffold DREAMS 2G in the Treatment of Subjects with de novo lesions in native coronary arteries) (18). It has been the first study to assess the safety and performance of a novel Sirolimus-eluting absorbable magnesium scaffold in symptomatic patients with de novo coronary artery lesions.

The primary endpoint of this prospective and multicenter single-arm study was in-segment LLL at 6 months post-procedure. 123 subjects have been enrolled in the study with clinical follow up planned at 1, 6, and 12 months and annually thereafter until 5 years post-procedure. An angiographic follow up has been performed at 6 months post procedure for 113 implanted subjects. For a subset of up to 30 evaluable subjects, intravascular ultrasound (IVUS) and optical coherence tomography (OCT) have been performed at 6-month follow-up. At one center, vasomotion has been assessed with acetylcholine followed by nitroglycerin in a subgroup of 25 subjects.

Mean in-segment LLL at 6 months was 0.27±0.37 mm. In segment LLL decreased by 41.8% (from 0.52 mm to 0.27 mm) from BIOSOLVE-I to BIOSOLVE-II with DREAMS 2G. This result is better than the LLL of the ABSORB BVS Cohort A (46) (0.36±0.29 mm) whereas it is inferior to the LLL of the ABSORB Cohort B1 (37) (0.11±0.28 mm) at 6 months. Notably, as late lumen enlargement and favourable tissue response were reported for the absorbable scaffolds (34,47), LLL might not be as relevant for long

term outcomes. For instance, with a 0.44 mm LLL at the 6-month follow-up, the 5-year outcome of the ABSORB Cohort A was excellent without any additional MACE (47). However, these results need to be confirmed in larger studies.

The neointimal hyperplasia area at 6 months was 0.08 mm^2 and 0.30 mm^2 in BIOSOLVE-I and BIOSOLVE-II, respectively. These are comparable to those of the ABSORB Cohort B (0.08 mm^2)(37) and of ABSORB Cohort A (0.30mm^2)(46). The decrease of radial strength during the absorption process might explain some of the variations in neointimal hyperplasia across these studies.

IVUS analysis in a subgroup showed that resorption of DREAMS 2G is slower than DREAMS 1G whose resorption time was too fast: mean scaffold and mean lumen area decreased faster in BIOSOLVE-I (10) than in BIOSOLVE-II (18) (mean scaffold area –11.1% vs –0.5%; mean lumen area –15.3% vs –2.4%).

No malapposed struts were detectable during the OCT analysis as the investigational device was fully embedded in the vessel wall. Furthermore, 80% of the vasomotion subgroup subjects showed vasoconstriction or vasodilatation with a threshold of change of ≥3.0% (18).

TLF was 3% at 6 months which is similar to the TLF rate of new generation metallic stent (39). One target vessel MI occurred (<1%) and 2 subjects underwent clinically driven TLR (2%). One death of unknown cause (<1%) was classified as cardiac death. No definite or probable scaffold thrombosis was observed in BIOSOLVE-II (18), in line with the previous studies performed with precursor devices (10,33).

In summary, the 6 month follow-up results of the BIOSOLVE-II study reveal that DREAMS 2G improves late lumen loss compared to its precursor devices while maintaining a favorable clinical and safety profile (10,33).

From 6 to 12 months, quantitative coronary angiography (QCA) parameters remained stable (paired data of 42 patients: in-segment late lumen loss 0.20 mm vs. 0.25 mm, P = 0.117; in-scaffold late lumen loss 0.37 mm vs. 0.39 mm, P = 0.446). IVUS and OCT findings corroborated the QCA results. Furthermore, no additional TLF event occurred between 6 months and 12 months; and none of the patients experienced a definite or probable scaffold thrombosis (11).

At 24 months, TLF rate was 5.9%, based on two cardiac deaths (1.7%), one target vessel myocardial infarction (0.9%) and four TLR (3.4%). There was no definite or probable scaffold thrombosis (19).

Between two and three years, only one additional TLR occurred, resulting in a TLF-rate of 6.8%, including 1.7% cardiac death, 0.9% target vessel MI, and 4.3% clinically driven TLR. No definite or probable scaffold thrombosis occurred (20).

In summary, 3-year results of BIOSOLVE-II demonstrate that the device iteration DREAMS 2G resulted in improved LLL compared to its precursor devices while maintaining a favourable clinical and safety profile. The study has been prolonged to add an additional 60-month follow-up.

BIOSOLVE-III

The BIOSOLVE-III study is a prospective, multicenter study conducted in 61 subjects enrolled at 8 investigational sites. Clinical follow-up visits take place at 1, 6, and 12 months and annually until 3 years post-procedure, with an angiographic follow-up at 12 months. Primary endpoint is in-hospital procedural success.

BIOSOLVE-II and BIOSOLVE-III considered together have enrolled 184 patients. BIOSOLVE-II and III pooled data at 6 month follow-up showed 3.3% TLF rate, consisting of two cardiac deaths (1.1%), one target vessel myocardial infarction (0.6%), and three clinically driven TLR (1.7%) (19). In BIOSOLVE-III, there were significantly more type B2/C lesions than in BIOSOLVE-II (80.3% vs 43.4%, P<0.0001) and significantly more moderate-to-severe calcifications (24.2% vs 10.7%, *P*=0.014). In spite of that, the clinical event rates remained stable. There was no definite or probable scaffold thrombosis (21).

At 12 months, there was no difference in LLL between BIOSOLVE-II and BIOSOLVE-III (21). In the overall population, it was 0.25±0.31 mm in-segment and 0.39±0.34 mm in-scaffold. No additional TLF occurred between 6 and 12 months and no definite or probable scaffold thrombosis was observed.

BIOSOLVE-IV

BIOSOLVE-IV is a prospective multi-centre registry including more than 2000 patients in a real world setting, involving up to 130 centres and 30 countriesThe following event rates are reported for the first 1075 patients up to 12 months follow up: 4.3% for TLF, 0.2% for cardiac death, 1.1% for target vessel MI, 3.9% for clinically driven TLR, and 0.5% for definite or probable scaffold thrombosis (22).

All subjects suffering a scaffold thrombosis also had a target vessel MI and a clinically driven revascularization and four of them additionally underwent DAPT discontinuation at day 4, 5, 46 and 95 post implantation, respectively.

As several recent publications of ABSORB BVS raised concerns about elevated scaffold thrombosis rate for ABSORB, the sample size of the BIOSOLVE-IV registry has been increased to 2054 subjects to add a powered secondary endpoint to evaluate the superiority of Magmaris compared to ABSORB BVS in terms of scaffold thrombosis rate at 12 months follow up.

MAGNESIUM 2000

The Magnesium 2000 program was initiated to ensure a safe roll-out of the device into clinical practice and to review intraprocedural performance parameters. There was no restriction in form of in-or exclusion criteria and data were collected via anonymized post-market evaluation forms. As part of the certification process to use Magmaris, physicians needed to complete these post-market evaluation forms for their first cases (at least 10 cases were required for certification).

From June 2016 to May 2018, data on 2018 Magmaris implants were collected. The rationale to use Magmaris was patient’s life expectancy in 67% of cases.

Magmaris was successfully implanted in 99% of cases. The performance of Magmaris was rated as good or very good in 96% of cases, and compared to the ABSORB BVS scaffold, the conformability of Magmaris was rated better in 73% of cases, the crossability in 77%, the trackability in 74% and the pushability in 74% (23).

ACS-registry

The Magmaris-ACS Registry was designed as part of the Polish Magmaris registry. Fifty patients with acute coronary syndrome (ACS), without STEMI, were enrolled in this

single center registry. Angiographic success of the target lesion was 100%. One case of recurrent ischemia was observed at day 1 post-procedure due to a significant distal edge dissection, which resulted in an overlapping DES implantation. No other TLR events occurred until 6 months, and death, MI, and scaffold thrombosis was absent (24).

Single center registry (25)

In 35 patients, 40 Magmaris were implanted to treat 37 de novo lesions. The implantation was successful in all cases, in one case a non-flow limiting distal edge dissection occurred after implantation and before post-dilatation.

Post-dilatation with a noncompliant balloon led to significantly larger mean scaffold diameter (3.21 ± 0.32 mm vs. 2.80 ± 0.39 mm, P < 0.001), abluminal scaffold area (7.92 ± 1.43 mm2 vs. 6.72 ± 1.28 mm2, P < 0.001) and lumen area (7.58 ± 1.1 mm2 vs. 6.83 ± 1.12 mm2, P < 0.001). Incomplete scaffold apposition area was significantly lower if post-dilatation was performed (0.01 ± 0.04 mm2 vs. 0.17 ± 0.11 mm2, P < 0.001). Strut fractures were not observed before or after post-dilatation.

Clinical outcomes at 6 months showed 2 target lesion failure (6%) caused by TLR. There was no death, myocardial infarction or scaffold thrombosis.

STEMI-Pilot (26)

In this single-center prospective pilot study, the feasibility of the implantation of Magmaris in STEMI patients was assessed. 18 patients were enrolled. The primary endpoint was device-oriented composite endpoint (DOCE), including cardiac death, target-vessel myocardial infarction, and target-lesion revascularization (TLR) within 30 days of the index procedure. Secondary endpoints were procedural success, any probable/definite scaffold thrombosis, and DOCE at subsequent follow-up.

Procedural success was 100%, and there were no primary endpoint events. After a median follow-up of 153 days (range 59–326 days), one TLR occurred (5.6%) at 102 days post-procedure; but cardiac death, TV-MI and scaffold thrombosis was absent.

MAGSTEMI

MAGSTEMI is a prospective, controlled, randomized, multi-center trial comparing Magmaris with Orsiro in STEMI patients. The primary endpoint was vasodilatory response ≥ 3% in response to nitroglycerin at 1 year. 76 patients were implanted with Orsiro, 74 with Magmaris. At 1-year, vasoreactivity was significantly higher with Magmaris (27).

1.2 Other Manufacturers‘ Prior Investigations

The bioabsorbable scaffold with the most clinical evidence available is ABSORB BVS (Abbott Vascular, Santa Clara, CA), which consists of a backbone of poly-L-lactide (PLLA) coated with poly-D-L-lactide controlling the release of the antiproliferative drug Everolimus. The first-in-man ABSORB (Cohort A) trial was designed to assess the safety and performance of the first generation scaffold with an enrolment of 30 subjects (46). This trial revealed a very good clinical safety as there was no cardiac death, ID-TLR or scaffold thrombosis during 2 years follow-up (48). A 3.4% ischemia-driven MACE (ID-MACE) rate with only one non-Q wave MI in the 2 years after index procedure was promising as well. No additional event occurred until 5 years (49). Angiographic results such as an in-scaffold late lumen loss of 0.44±0.35 mm and 0.48±0.28 mm at the 6 months and 2 years follow up, respectively, and a diameter stenosis of 27% for both the 6-month and 2-year follow-ups were comparable to some of the permanent DESs (50,51) and better than BMS (52).

As the ABSORB Cohort A trial revealed that the initial generation of the scaffold had some limitations such as a high rate of incomplete strut apposition and late acquired malapposition due to poor conformability, scaffold shrinkage, and chronic recoil problems (46,48), a second generation bioresorbable everolimus-eluting scaffold systems was developed (ABSORB BVS 1.1). The ABSORB Cohort B trial enrolled 101 subjects (45 subjects in Cohort B1 and 56 subjects in Cohort B2) treated with ABSORB BVS 1.1 and showed very good 6-month angiographic results with an in-scaffold late lumen loss of 0.19±0.18 mm in Cohort B1 (37) and 0.27±0.32 mm in Cohort B2 (53) at 6 months and 1 year follow up, respectively. These results are similar to permanent DESs (40,51,54). At 5 years, there were 3% deaths, no cardiac death, 3% MI, 8% ischemia-driven TLR and 11% MACE events; definite or probable scaffold thrombosis

was absent (55). The first-in-man ABSORB Cohort A study and subsequently the Cohort B study thus showed safety and efficacy of the scaffold under clinical study conditions.

Many clinical trials and registries were then initiated to expand the experience with scaffold system with broader inclusion criteria for the treatment of complex lesions, subjects in real-world settings, and in different geographies.

First, the multicentre, randomised, single-blind, active-controlled ABSORB-II trial comparing the ABSORB everolimus-eluting bioresorbable scaffold versus the Xience everolimus-eluting stent was initiated. This trial is the first randomised controlled comparison of a bioresorbable scaffold with a DES. The study enrolled 501 subjects and showed similar 1 year composite secondary clinical outcomes between ABSORB BVS and Xience arms (56). The TLF rate in the ABSORB and Xience groups was 5% and 3% (p=0.35), respectively. The clinically indicated TLR (CI-TLR) rate was 1% for the ABSORB arm and 2% for the Xience arm (p=0.69). Definite and probable scaffold or stent thrombosis rate was 0.9% and 0% (p=0.55), respectively. (56). At 4 years, TLF was 11.1% for ABSORB and 5.6% for Xience, p=0.050, and the ABSORB group had significantly more myocardial infarctions (8.6% versus 3.3%, p=0.0363) and definite or probable scaffold thrombosis (2.6% versus 0%, p=0.0347) (57).

ABSORB III is a multicentre, randomised trial (2:1 ABSORB vs Xience) with 2008 subjects at 220 US and non-US sites (58). TLF at one year was 7.8% and 6.1% in the ABSORB and Xience groups, respectively, demonstrating non-inferiority for the primary endpoint of the study. The device thrombosis rate of the ABSORB and Xience arms was 1.5% and 0.7% (p=0.13), respectively (58). At 3 years, TLF was 13.4% versus 10.4%, p= 0.06, TV-MI was 8.6% versus 5.9%, p=0.03, ID-TLR 7.2% versus 5.9%, p=0.27, and definite or probable scaffold thrombosis was 2.3% versus 0.7% (p=0.01) (8).

The AIDA trial was a single-blind multi-center investigator-initiated trial (IIT) that compared ABSORB with the Xience everolimus-eluting stent and showed non-inferiority for ABSORB. 924 patients with 1237 lesions were treated with the ABSORB scaffold and 921 patients with 1209 lesions with the Xience DES. At 2 years, TLF was 9.7% versus 8.0% (p=ns), cardiac death 1.9% versus 2.2% (p=ns), target-vessel MI 5.1% versus 3.1% (p=0.034), and TLR 6.5% vs 4.8% (p=ns). Definite or probable device thrombosis was 3.3% vs 0.9% (p<0.001) and definite scaffold thrombosis 2.9% versus 0.5% (p<0.001) (9).

Since these studies showed more events for BRS than for DES, the active controlled, blinded, multicentre randomized ABSORB IV trial was initiated that compared the ABSORB BVS scaffold with the Xience stent and that used optimised implantation techniques and an expanded patient population including patients with ACS in which scaffolds might be particularly useful. 1296 patients were assigned to ABSORB and 1308 to Xience. Outcomes showed non-inferiority of ABSORB, with a 30-day TLF rate of 5.0% for ABSORB and 3.7% for Xience patients and a one-year rate of 7.8% versus 6.4%. Device thrombosis occurred in 0.7% versus 0.3%, p=0.1586 (59).

Several registries have also been initiated to obtain more evidence in a real world patient population, including:

- ABSORB EXTEND, a prospective single-arm study with an enrolment of 812 patients at 56 sites. Cardiac death at one and 3 years was 0.7% and 2.1%, MI 3.0% and 4.0% and ID-TLR 1.4% and 3.1%. The rate of definite/probable scaffold thrombosis was 1.0% and 2.2% (60)
- The GHOST-EU registry enrolled 1189 subjects and reported an acceptable 6-month TLF rate (4.4%) although early and midterm scaffold thrombosis rate were high (61). In 2016, Tamburino et al reported a 1.8% definite or probable scaffold thrombosis rate at 12-month follow up (62).
- The ABSORB UK registry enrolled 1005 subjects at 23 sites. At 12 months TLF was 3.2% and definite or probable scaffold thrombosis 1.7% (63).
- IT-Disappears enrolled 1002 subjects with a high atherosclerotic burden and multivessel disease at 50 sites in Italy (64). The primary endpoint of the study was the device-oriented composite endpoint (DOCE, syn TLF), a composite of cardiac death, target vessel MI, and ischemia-driven TLR at 12 months. The rate of DOCE was 9.9% and the rate of definite or probable scaffold thrombosis was 0.9%.
- ISAR-ABSORB is a registry that enrolled 419 subjects with symptomatic coronary artery disease. The rate of definite or probable scaffold thrombosis at 12 month follow up was 3.1%(65). At 2 years, rate of cardiac death was 3.1%, rate of myocardial infarction 3.9%, TLR 16.0%, definite scaffold thrombosis 3.8%, and definite or probable scaffold thrombosis 4.2% (66).

- The FRANCE ABSORB registry enrolled 2072 patients at 86 centers (67). At 1 year, TLF was 3.5%, cardiac death 0.6%, target-vessel-MI 1.9%, TLR 2.4%, definite scaffold thrombosis 1.2% and definite or probable scaffold thrombosis 1.5%.

Some of the trials above raised concerns related to the safety of ABSORB. A systematic review and meta-analysis of available randomized and nonrandomized studies demonstrated a 2-fold higher incidence of scaffold thrombosis after ABSORB BVS implantation compared with implantation of permanent metallic DES (68). More recently also higher incidences of target vessel-related myocardial infarction have been reported (8,9).

Other bioresorbable scaffolds with limited clinical evidence also exist and are well summarized by (69).

Some evidence is available for the DESolve novolimus-eluting polymeric scaffold:

- The initial study enrolled 126 patients. TLF was 5.7% at one year and 7.4% at 2 years, cardiac death was 1.6% and 2.5%, target-vessel MI was 0.8% at one and 2 years, and TLR was 3.3% and 4.1%. Definite stent thrombosis occurred in 0.8% at one and 2 years (70).
- A post-market follow-up study enrolled 102 patients at 10 European sites. At 12 months, the DOCE was 3%, consisting of TLR. Definite scaffold thrombosis was 1.0% (71).

Little published evidence is available for the Fantom sirolimus-eluting scaffold:

- Cohort A of the FANTOM II Study enrolled 117 patients. At 6 months, no patient had died, 2 (1.7%) had an MI and 2 (1.7%) a clinically driven TLR. Definite scaffold thrombosis occurred in 1 patient (0.9%) (72).

1.3 DREAMS 3G and BIOMAG-I

So far, the clinical trial results have demonstrated very low adverse event rates for Magmaris. While other bioresorbable scaffolds showed relatively high rates of thrombosis, thrombosis rates were low across all Magmaris and precursor studies and consistent with contemporary DES thrombosis rates.

Potential factors contributing to this difference in clinical outcomes, include different scaffold design and materials, reduced thrombogenicity, as shown in animal models (13,73), shorter resorption time, and the BIOlute™ coating as that is also used in the Orsiro Sirolimus Eluting Coronary Stent that has demonstrated consistently low TLF rates in randomized clinical trials (41,42,44,45).

Despite these positive results, iterative improvement of Magmaris, to enhance performance and usability led to the development of the next generation scaffold, DREAMS 3G. This scaffold is built with a refined magnesium alloy and enhances some scaffold properties, such as radial strength, scaffolding time and marker visibility, broadens the size range and reduces crossing profile and strut thickness. All of these changes are meant to improve the overall clinical outcomes.

BIOTRONIK is proposing to evaluate the safety and performance of next generation DREAMS 3G in a clinical trial program in a first in man study: BIOMAG-I.

1.4 Treatment recommendations

Treatment recommendations in coronary artery disease include either medication therapy or a percutaneous interventional procedure with a DES for example. In the ECS_EACTS guidelines 2018, current generation BRS for clinical use outside clinical studies is a class III recommendation only (74). This trial will further evaluate the safety and efficacy of a next generation BRS within a clinical study.

2. INVESTIGATIONAL DEVICE

A detailed description of the device is provided in the latest Investigator Brochure (CIB) version.

2.1 Manufacturer of the device

BIOTRONIK VI / Ackerstrasse 6/ 8180 Bülach / Switzerland

2.2 Intended purpose

The Dreams 3G device is indicated for improving the luminal diameter for treatment of de novo coronary artery lesions by PTCA.

2.3 Device Description

DREAMS 3G is a scaffold system consisting of a balloon-expandable bioabsorbable scaffold pre-mounted on the balloon of a rapid-exchange PTCA catheter. The scaffold backbone is made from bioabsorbable Magnesium and contains two permanent x-ray markers made from Tantalum on the distal and on the proximal scaffold end, respectively. The surface of the scaffold backbone is completely coated with bioresorbable PLLA (Poly-L-Lactidic Acid) which incorporates Sirolimus. Sirolimus load is 1.4 ± 0.3 µg per mm^2 scaffold surface.
The DREAMS 3G scaffold system is packaged in a dispenser. The dispenser is placed in an aluminium Tyvek pouch. The device is sterilized using ethylene oxide sterilization.

In vivo investigations using a porcine model show that DREAMS 3G provides vessel support for about 3-6 months and releases around 70% Sirolimus from the PLLA carrier during a period of 3 months after implantation.

2.4 Use of the device(s) during the investigation

Please see the instructions for use (IFU) for a detailed description of the use of the device during the intervention.

2.5 Necessary training / experience for the use of the investigational device

The implanting physicians have to be trained on the consensus paper (75), the IFU, and the “4 P” program of BIOTRONIK AG (Appendix 3).

2.6 Size Matrix

Dreams 3G devices will be available in the following sizes:

Table 2: Investigational device sizes:

Scaffold diameter (mm) \ Scaffold length (mm)	13	22	30
2.5	x	x	
3	x	x	x
3.5	x	x	x
4	x	x	

2.7 Preclinical Testing

DREAMS 3G has undergone preclinical testing in order to assure the safety and performance of the device according to relevant standards and guidelines. Amongst others, these tests considered in vivo evaluation in porcine model, biocompatibility, sterility, drug release characteristics, mechanical properties and device stability.

Note: More detailed description about the DREAMS 3G can be found in the IFU and the CIB.

2.8 Current state of the art in clinical care in the relevant field of application

The current state of the art in clinical care of coronary artery disease consists of either medication therapy alone or in combination with implantation of a permanent DES (74). The use of current generation of BRS outside of clinical studies is currently only a class III recommendation according to the guidelines (74). In BIOMAG-I, a next generation of a Mg based bioresorbable scaffold will be tested.

2.9 Device Supply / Device Labeling

The investigational device and its associated components will have a label according to Medical Devices Regulation (EU) 2017/745 (MDR) (version 2017) that will be visible on the storage containers and sterile barriers. The labels or manuals will bear the following information:

- Name, model and lot number of the device
- Name and addresses of the manufacturer
- Labeling statement: “Exclusively for Clinical Investigations”.
- Quantity of contents
- All relevant contraindications, hazards, adverse device effects, interfering substances or devices, warnings and precautions
- Expiration date

2.10 Device Storage

Store according to IFU; the study device has to be stored in a dark and dry location at a temperature from 15 to 25 °C.

2.11 Device traceability

In this pre-market clinical investigation, the device identification and traceability is secured by the use of a device accountability log.

2.12 Device Accountability

Access to investigational devices shall be controlled and their use limited to the clinical investigation according to the CIP.

The sponsor shall keep records to document the physical location of all investigational devices from their shipment to the investigation sites until their disposal or return. The principal investigator or an authorized designee shall keep records documenting the receipt, use, return and disposal of the investigational devices, which shall include:

- Date of receipt
- Identification of each investigational device (batch number/serial number or unique code)
- Expiry date
- Date of use
- Subjects identification code, if applicable
- Date on which the investigational device was returned/explanted from subjects, if applicable
- Date of return of unused, expired or malfunctioning investigational devices, if applicable.

2.13 Recall of investigational devices

In case of a recall, the Field Safety Notice and Customer Acknowledgement Form should be prepared by complaint management according to BIOTRONIK AG internal working instructions. The documents are provided to Medical Affairs who is responsible for the following steps:

- Inform clinical study sites
- Inform respective national competent authorities, ethic committees and all other relevant authorities
- Collect affected devices if applicable
- Collect completed Customer Acknowledgement Forms and provide forms to post market surveillance
- Confirm closure of all actions to post market surveillance

3. RISK-BENEFIT-ANALYSIS

3.1 Potential Risks

The risk assessment, bench testing and pre-clinical animal testing used to support the safety of the DREAMS 3G are documented in the CIB.

As with any subjects undergoing percutaneous coronary intervention, subjects may experience adverse events and/or outcomes that are listed in the IFU and are not expected to difer from other contemporary drug eluting stent implantation procedures.

Table 3: Overview of potential adverse events associated with PTCA, stent and scaffold placement that can occur when using the deive as intended

**Potential adverse events associated with PTCA, stent and scaffold placement that can occur when using the device as intended:**		**Frequency**	**References**
Cardiac events	Myocardial infarction or ischemia, abrupt closure of coronary artery,<br>restenosis of treated artery,<br>cardiogenic shock,<br>angina,<br>tamponade, perforation or dissection of coronary artery or aorta, cardiac perforation, emergency cardiac surgery, pericardial effusion, aneurysm formation	0-2.9%<br>0-0.3%<br><br>0-18.6%<br>1.1-4.6%<br>0-26%<br>0-2.3%	(11,76,77)<br>(78)<br><br>(79–81)<br>(82,83)<br>(84,85)<br>(82,86–89)
Arrhythmic events	Ventricular tachycardia, ventricular fibrillation, atrial fibrillation, bradycardia	1.2-42%	(83,90–93)
Scaffold system events*	Failure to deliver scaffold to intended site, scaffold dislodgement from the delivery system, scaffold misplacement, scaffold deformation, scaffold embolization, scaffold thrombosis or occlusion, scaffold fracture, scaffold migration, inadequate apposition or compression of scaffold /s, inflation difficulties, rupture or pinhole of balloon,	0-7%	(10,94,95)

Potential adverse events associated with PTCA, stent and scaffold placement that can occur when using the device as intended:		Frequency	References
	deflation difficulties,<br>withdrawal difficulties,<br>embolization of catheter material.		
Respiratory events	Acute pulmonary edema, congestive heart failure, respiratory insufficiency or failure	0.9-11.6%	(96–98)
Vascular events	Access site hematoma,<br>hypotension/ hypertension,<br>pseudoaneurysm,<br>arteriovenous fistula formation, retroperitoneal hematoma,<br>vessel dissection or perforation,<br><br><br><br><br><br>restenosis,<br>thrombosis,<br><br>compromise of side branch patentcy, or occlusion,<br>vasospasm, peripheral ischemia, dissection, distal embolization (air, tissue	0.3-0.62%<br>0-4.8%<br>0-2.3%<br><br><br><br>Up to 30% (and 0.3-0.6% perforation)<br><br><br><br>0-1.0 %<br><br><br><br>0-0.4% (For vasospam;	(99)<br>(92)<br>(88,89,100)<br><br><br>(82,101)<br><br><br><br><br><br><br>(11,70,71,77,102)<br>(103)

**Potential adverse events associated with PTCA, stent and scaffold placement that can occur when using the device as intended:**		**Frequency**	**References**
	debris, thrombus)	for the other events prevalence not reported in literature)	
Neurological events	Permanent (stroke) or reversible (TIA) neurologic event, femoral nerve injury, peripheral nerve injury	0-0.5%	(82,104)
Bleeding events	Access site bleeding or hemorrhage, hemorrhage requiring transfusion or other treatment	0.3-1.8%	(99)
Allergic reactions	To contrast media, tantalum, aluminium, anti-platelets, anticoagolants, to the drug carrier PLLA, to Sirolimus (derivates or similar drugs)	<1.0%	(139)
Allergic reactions	Magnesium and aluminium	Estimated <1%	(140)**
Infection and sepsis		<1.0%	***
Death	All cause	0-1.7%	(24,95)

* The sumof all scaffold system events that have occurred are considered as device failure rate

** Allergic reactions against magnesium are generally very rare and were not observed in previous studies with the Magmaris scaffold. There is no data published regarding implants containing aluminium. Generally, patients who develop problems after implantation of a metal

implant seem to have a higher in-vitro allergization against other metals (e.g. 52% of patients with a metal implant with a high allergization against nickel also developed an allergization against aluminium and vanadium (140). Allergic reactions against aluminium cannot be excluded but are estimated to occur with a frequency less than 1%.

*** No data published on infection and sepsis associated with a PTCA as it hardly occurs.

Potential adverse events related to sirolimus (following oral administration) include but are not limited to: Abnormal liver function, anemia, arthralgia, diarrhea, hypercholesterolemia, hypersensitivity, including anaphylactic/ anaphylactoid type reactions, hypertriglyceridemia, hypokalemia, infections, interstitial lung disease, leukopenia, lymphoma and other malignancies, thrombocytopenia. Appropriate contraindications and warnings are included in the IFU provided with the study device.

For all subjects, quantitative coronary angiography including IVUS and OCT will be performed at 6 and 12 months follow-up. Coronary angiography, IVUS and OCT are common medical tests. They rarely cause serious problems. However, complications can occur and include, but are not limited to:

- Bleeding, infection, and pain at the catheter insertion site
- Damage to blood vessels (including dissections)
- Allergic reaction to contrast media or medication

Other, less common complications include: arrhythmias, kidney damage, blot clots that can trigger a stroke, heart attack, or other serious problems, low blood pressure, pericardial effusion

The vasomotion test involves an infusion of acetylcholine, which can provoke a pathological narrowing of the vessel, which in turn may lead to chest pain, reduced blood supply to the heart, cardiac arrhythmia or an occlusion of the blood vessel. As the vasomotion test is performed during the coronary angiography, these side effects are generally easily controlled.

3.2 Potential Benefits

Coronary stents have improved significantly the immediate and long-term results of percutaneous coronary interventions. However, once the vessel has healed, the

scaffolding function of the stent is no longer needed, and the presence of a permanent metallic stent poses important disadvantages. Bioresorbable scaffolds have been introduced with the aim to overcome the limitations posed by the presence of a permanent implant. The basic concept is based upon the degradation of the stent backbone to inert particles after its function has been achieved (105–107).

One benefit of bioresorbable scaffolds is that the artery would not be permanently caged and late positive remodeling in response to physiological stimulus (restoration of vasomotion) would be possible (5,108). Persistent impairment of endothelial vasomotor function after treatment has been associated with adverse cardiovascular events at the long-term follow-up. Therefore, the restoration of coronary vasomotion is essential after percutaneous coronary intervention (107). In the BIOSOLVE II trial, angiographically discernible vasomotion was documented after implantation of the Magmaris (DREAMS 2G) scaffolds in 80% of the patients evaluated at 6 months (95).

The resorption of the scaffold would also allow future treatments in the vessels if needed (either percutaneous or surgical) and would facilitate the access to side branches initially jailed by the scaffold (109). Moreover it would avoid that subjects have several metal layers in their arteries. The resorbability of the scaffold also has important psychological implications. Many patients are concerned about having a permanent implant in their coronary arteries and would prefer a device that is able to disappear after a determined time (109).
The implantation of a resorbable device may also facilitate non-invasive imaging technologies, like CT or MRI, because they do not create any of the artifacts originating from permanent metallic stents (110–112).

Permanent stents are associated with a certain risk of stent thrombosis and in-stent restenosis. For bioresorbable scaffolds, the risks associated with stent thrombosis and in-stent restenosis would theoretically be removed once the scaffold has been fully degraded (105–107).
To determine its thrombogenicity, Magmaris was tested against the ABSORB in an ex-vivo arteriovenous porcine shunt model. Despite a similar scaffold strut thickness, the Magmaris scaffold was significantly less thrombogenic compared with the ABSORB (13). Similarly, a study in porcine and rabbit models showed an increased endothelialization

and decreased thrombus formation for DREAMS 2G compared to ABSORB. Inflammation for Magmaris peaked at 90 days and decreased thereafter; at one and two years, inflammation was lower for Magmaris versus an everolimus-eluting cobalt-chromium stent (14). Clinical evidence from several trials associated the ABSORB BRS with an elevated thrombosis risk compared to DES and its commercial distribution was stopped. For Magmaris, no elevated thrombosis risk was identified in clinical experience. No definite or probable scaffold thrombosis was reported in the BIOSOLVE-II and BIOSOLVE-III studies up to 36 and 12 months, respectively. In the BIOSOLVE-IV study, the definite/probable scaffold thrombosis rate was 0.5%, whereby 4 of the 5 scaffold thrombosis cases were associated to early DAPT interruption, in the full cohort up to 12 months which is comparable to DES.

Another contributing factor to the risk of scaffold thrombosis is the scaffold properties. Polymer coating, strut dimensions, and strut positioning against the vessel wall are considered critical factors influencing thrombogenicity (1). The percentage of uncovered or malapposed struts is one predictor of late stent thrombosis (107). Protruding struts disrupt the laminar flow and cause the induction of endothelial shear stress and platelet aggregation. Malapposed struts show higher flow disturbances around the edges and increase the risk of platelet activation and thrombus aggregation. In the BIOSOLVE-I study Magmaris showed a high rate of strut apposition, which was higher than the rate of strut apposition achieved with ABSORB (10). In the BIOSOLVE-II study, no mal-apposed struts were present at 6 months because the Magmaris scaffold struts were already fully embedded in the vessel wall. No intraluminal mass was observed at any time (11). Compared to ABSORB the round-shaped edges of Magmaris and DREAMS 3G cause less disturbance of the blood flow and therefore contribute to a lower thrombogenicity. In addition, the faster resorption of Magmaris and DREAMS 3G (1 year) compared to ABSORB (3-4 years) is expected to reduce the risk of late events (1).

Neoatherosclerosis has also been shown to play a pathogenic role in development of both scaffold thrombosis and in-stent restenosis (113)(114). By reducing vascular constraints and vessel rigidity, bioresorbable scaffolds have been postulated to improve flow dynamics and consequently reduce the incidence of neoatherosclerosis. Recent data stemming from the direct comparison of resorbable magnesium scaffolds and conventional DES in pre-clinical models lend support to this hypothesis, by showing a significant reduction of neointimal macrophages, a sign of early neoatherosclerosis in the former group (115). In line with these pre-clinical findings, retrospective OCT analysis of

21 patients at baseline and at 3-year follow-up (2 years beyond the resorption time) from the BIOSOLVE-II study suggests favorable vascular healing and overall disease regression in 12 out of 21 patients (116).

Nevertheless, several limitations still exist for currently available BRS. These include thick struts (150 µm), scaffold deliverability, visibility, and expansion, as well as concerns about the provided radial strength (107). Magmaris (DREAMS 2G) was further developed into DREAMS 3G to address these limitations.

For DREAMS 3G, the scaffold structure design was adapted and a Magnesium alloy with increased mechanical strength was developed. Magnesium has superior mechanical properties with its higher tensile strength and greater % elongation-at-break compared with polymeric material. The Magnesium alloy used in the Magmaris offers higher deformation resistance and lighter weight as compared with pure Magnesium (1). The improved Magnesium alloy of DREAMS 3G scaffold contains Aluminum to support the required material characteristics regarding mechanical performance and resorption behavior. Accordingly, the DREAMS 3G scaffold alloy has an increased mechanical strength compared to the Magmaris alloy and provides a more uniform resorption. The improved scaffold acute mechanical properties are expected to support treatment of complex lesions with DREAMS 3G. In addition to higher mechanical strength, the improved Mg alloy of DREAMS 3G will provide an increased scaffolding period. At the same time, the Mg resorption period of 12 months is maintained. The increased scaffolding period is expected to further improve chronic luminal outcome for DREAMS 3G in comparison to Magmaris without compromising safety.

In comparison to Magmaris, the scaffold strut dimensions of DREAMS 3G were further decreased to further reduce the risk of thrombosis. Compared to the 150 µm strut thickness of Magmaris, the strut thickness of DREAMS 3G is 117 µm for scaffolds with a diameter of 3 and 3.5 mm. Additional sizes were added to the device portfolio of DREAMS 3G. The increased size range of the DREAMS 3G product portfolio will allow for treatment of smaller/larger vessels and longer/shorter lesions in comparison to Magmaris. The new Ø 2.5 mm DREAMS 3G scaffold has a strut thickness of 99 µm and the Ø 4 mm DREAMS 3G scaffold has a strut thickness of 147 µm. The polymeric scaffolds ABSORB and DESolve have a strut thickness of 150 µm and a strut width of 190 µm and 165 µm, respectively. With a maximum strut thickness of 147 µm and strut

width of 150 µm, DREAMS 3G will have a lower vessel coverage than the polymeric scaffolds.

The reduced scaffold strut dimensions will also contribute to improved deliverability of the device. In terms of deliverability, DREAMS does not require stepwise inflation as required for polymeric scaffolds (12), which contributes to a better scaffold expansion and apposition (11,15). To further improve scaffold deliverability of DREAMS 3G, the profile of the crimped scaffold was further reduced to support deployment in challenging vessel anatomy. In preclinical testing, trackability/deliverability of DREAMS 3G was rated excellent, same as the drug-eluting stent Orsiro.

To increase the scaffold marker x-ray visibility, the marker material density and marker dimensions were increased compared to Magmaris. This will support scaffold positioning and post-dilatation for DREAMS 3G.

3.3 Risk / Benefit Conclusion

Pre-clinical findings indicate that the Mg based scaffold is less thrombogenic, provokes less inflammatory response and reduces formation of new artherosclerosis when compared to other available scaffolds and DES which eventually results in improved endothelial integrity. Available data on DREAMS 2G on more than 1000 subjects with a low rate of target lesion failure and scaffold thrombosis further support these beneficial findings and show that the device is safe and efficient when used according to the instructions of use.

The iterations of the device from DREAMS 2G to DREAMS 3G have addressed potential disadvantages of DREAMS 2G while maintaining the characteristic features that are thought to be responsible for the beneficial pre-clinical and clinical findings. This FIM trial will provide further evidence of the safe use of bioresorbable Mg based scaffolds in the treatment of coronary artery lesions. The device will be used under controlled conditions and subjects will be closely monitored. Every precaution has been taken to protect the health and safety of the subjects.

Moreover, subjects will be closely monitored throughout the clinical investigation duration. They will be evaluated at pre-determined time points to assess their clinical status and follow-up angiographies (including additional imaging by IVUS and OCT) will be performed to assess the status of both target lesion and the scaffold.

An independent Data Monitoring Committee (DMC) will monitor safety throughout the clinical investigation. Stopping rules will be discussed with the DMC and applied for subject safety throughout enrollment.

Therefore we conclude that the benefits of the new DREAMS 3G device outweigh the risks that are involved within this study.

4. CLINICAL INVESTIGATION

4.1 Study Objectives

The study objective is the assessment of safety and clinical performance of the DREAMS 3G in de novo coronary artery lesion and in order to obtain CE-approval.

4.2 Hypothesis

As a primary endpoint, the BIOMAG-I trial will assess non-inferiority of the 6-month in-scaffold late lumen loss of the DREAMS 3G scaffold vs. in-scaffold late lumen loss of a historical control from clinical trials with PLLA scaffolds as well as metallic scaffolds.

**Scaffold**	**6 months In-scaffold LLL (mm) Mean (SD)**	**N**	**Publication**
**ABSORB**	0.44(0.35)	26	Ormiston 2008 (46)
**ABSORB**	0.19(0.18)	41	Serruys 2010 (37)
**Desolve**	0.19(0.19)	14	Verheye 2014 (94)
**Magmaris**	0.44(0.36)	113	Haude 2016 (11)
**Desolve**	0.20(0.32)	113	Abizaid 2016 (70)

Scaffold	6 months<br>In-scaffold LLL (mm)<br>Mean (SD)	N	Publication
**Fantom II**	0.25(0.4)	100	Chevalier 2019 (102)

4.3 Relevance of the trial in the context to the state of the art of clinical practice

This trial will provide further information on the efficacy and safety of a novel drug eluting absorbable Mg based scaffold. State of the art technologies (coronary angiography, OCT and IVUS) will be applied to evaluate the physiological healing process at the lesion site.

4.4 Study Design

This is a prospective, multi-center and single arm first-in-man trial that will be conducted in up to 15 investigational study sites.

It will enrol up to 115 subjects (including an estimated drop-out rate of 25% at 12 months follow up). Clinical follow up visits will take place at 1, 6, and 12 months and annually thereafter until 36 months post procedure.

Subjects will undergo an angiographic follow up (including OCT and IVUS) at 6 months and at 12 months post-procedure.

If subjects consent, vasomotion will be assessed angiographically with acetycholine followed by nitroglycerine at 12 months follow up.

Information on previous clinical investigations with predecessors of the device can be found in section 1.1. Concomitant medication guidelines are described in section 5.7.

4.5 Study population

The study population will consist of subjects with de novo coronary artery lesions who suffer from symptomatic coronary artery disease and who need a percutaneous coronary intervention (PCI). Only subjects with up to 2 single lesions in 2 separate vessels will be included.

4.6 Study Duration

The anticipated duration of this clinical investigation is approximately 3.5 years (First Subject In until Last Subject Out):

Enrolment duration:	approximately 15 months
Follow up phase:	3 years

4.7 Primary Endpoint

The primary endpoint is in-scaffold late lumen loss (LLL) at 6-month post-procedure. Late lumen loss is the most commonly used surrogate efficacy endpoint in coronary artery disease. The angiographic assessment of late lumen loss reflects the efficacy of the device independent of inter-subject variations of vessel diameters (117,118).

The DREAMS 3G starts to lose its mechanical integrity between 3 and 6 months and thus does not have any scaffolding properties after 6-month, which is the reason for choosing 6-month LLL as the primary endpoint. Additionally the DREAMS 3G should be fully absorbed at approximately 12 months post procedure, therefore an angiographic examination at 12 months is planned to assess LLL, % diameter stenosis and binary restenosis rate to further support the assessment of the efficacy and safety of the DREAMS 3G device.

4.8 Secondary Endpoints

Clinical

- Target Lesion Failure (TLF*) at 1, 6, 12, and annually up to 36 months follow up

- Cardiac death at 1, 6, 12, and annually up to 36 months follow up (according to ARC-1 definition)
- Target vessel MI at 1, 6, 12, and annually up to 36 months follow up**
- Clinically driven target lesion revascularization at 1, 6, 12, and annually up to 36 months follow up
- Clinically driven target vessel revascularization at 1, 6, 12, and annually up to 36 months follow up
- Definite and probable scaffold thrombosis rate at at 1, 6, 12, and annually up to 36 months follow up (according to ARC-2 definition)
- Procedure success: achievement of a final diameter stenosis of <30% by QCA, using any percutaneous method, without the occurrence of death, Q-wave or non-Q-wave MI, or repeat revascularization of the target lesion during the hospital stay.
- Device Success: final residual diameter stenosis of <30% by QCA, or visual assessment using the assigned device only with:
  - Successful delivery of the scaffold to the target lesion
  - Appropriate scaffold deployment
  - Successful removal of the delivery system

*defined according to ARC-2 and ARC-1 definition.

** periprocedural MIs will be adjudicated according to SCAI-definitions and ARC-2 definitions

Secondary clinical endpoints were selected as per recommendations of the American College of Cardiology (ACC) and the American Heart Association (AHA) (Ref. ACC/AHA CLINICAL DATA STANDARDS: “”, JACC VOL2014 ACC/AHA Key Data Elements and Definitions for Cardiovascular Endpoint Events in Clinical Trials. 66, NO. 4, 2015) (119).

Angiographic

- In-segment late lumen loss at 6 months
- In-scaffold and in-segment late lumen loss at 12 months
- Binary in-scaffold and in-segment restenosis rate at 6 and 12 months
- % in-scaffold and in-segment diameter stenosis at 6 and 12 months

OCT and IVUS

Descriptive analysis of vessel morphology, lesion composition and scaffold strut data

Vasomotion

Descriptive analysis of vessel movement

4.9 Inclusion Criteria

1. Subject is ≥ 18 years and ≤ 80 years of age
2. Written subject informed consent available prior to PCI
3. Subject eligible for PCI according to the 2018 ESC/EACTS Guidelines on myocardial revascularization (72)
4. Subjects with a maximum of two single lesions in two separate coronary arteries which have to be de novo lesions and can be covered with 1 device each
5. Reference vessel diameter between 2.5-4.2 mm by visual estimation, depending on the scaffold size used
6. Target lesion length ≤ 28 mm by visual estimation, depending on the scaffold size used
7. Target lesion stenosis by visual estimation ≥ 50% - < 100% and TIMI flow ≥1 (assisted by e.g. QCA / IVUS /FFR).
8. Subjects with stable or unstable angina pectoris or documented silent ischemia or hemodynamically stable NSTEMI patients without angiographic evidence of thrombus at target lesion
9. NOTE: patient with acute STEMI can not be included in the study (according to exclusion criteria 2)Subject who has no contraindication for Dual Anti Platelet Therapy (DAPT)

4.10 Exclusion Criteria

1. Pregnant or breast-feeding females or females who intend to become pregnant during the time of the study.

2. Subject has clinical symptoms and electrocardiogram (ECG) changes consistent with acute ST elevation myocardial infarction (STEMI) within 72 hours prior to the index procedure.

NOTE: after 72 hours, any lesion other than the one causing the acute STEMI (culprit lesion) in any other epicardial vessel, may be treated according to the inclusion and exclusion criteriaLeft main coronary artery disease

3. Left main coronary artery disease

4. Three-vessels with coronary artery disease requiring treatment at time of procedure, including: left main, left anterior descending artery (LAD) right coronary artery (RCA) and circumflex coronary artery (Cx)

5. Planned interventional treatment of any non-target vessel within 12-month post-procedure

6. Subjects on dialysis

7. Impaired renal function (serum creatinine > 2.5 mg/dl or 221 μmol/l, determined within 72 hours prior to intervention)

8. Planned future intervention of a second lesion within the target vessel.

9. Ostial target lesion (within 5.0 mm of vessel origin)

10. Target lesion involves a side branch >2.0 mm in diameter

11. Documented left ventricular ejection fraction (LVEF) ≤ 30% within the last 6 months

12. Heavily calcified lesion which can not be adequately pre-dilated by a non-compliant and/ or scoring balloon as described in exclusion criteria 15.

13. Target lesion is located in or supplied by an arterial or venous bypass graft.

14. Target lesion requiring treatment with a device other than the non-compliant pre-dilatation balloon or scoring balloon prior to scaffold placement (including but not limited to rotational atherectomy, etc.).

15. Unsuccessful pre-dilatation, defined as a residual stenosis rate more than 20%, estimated by any method and / or angiographic complications (e.g. distal embolization, side branch closure, extensive dissections).

16. Known allergies or intolerances to: Acetylsalicylic Acid (ASA), P2Y12 inhibitors, Heparin, Contrast medium, Sirolimus, or similar drugs; or the scaffold material (Magnesium, Aluminium).

17. Subject is receiving an oral or intravenous immuno-suppressive therapy (e.g., inhaled steroids are not excluded) or has a known life-limiting immunosuppressive or autoimmune disease (e.g., human immunodeficiency virus, systemic lupus erythematosus) diabetes mellitus is not excluded).

18. Life expectancy less than 1 year.

19. Subjects under oral anticoagulation therapy (OAC) prior to implantation of DREAMS 3G unless DAPT can be maintained for a minimum of 6-month.

    Recommendation: If a subject requires OAC after DREAMS 3G implantation, DAPT should be maintained until 6 months follow up. Afterwards DAPT can be downsized to either ASA or Clopidogrel alone together with OAC for the remaining time period up to 12 months. After this, OAC monotherapy can be prescribed if still required.

20. Planned surgery or dental surgical procedure within 6 months after index procedure unless DAPT will be maintained.

21. In the investigators opinion subject will not be able to comply with the follow-up requirements.

22. Subject is currently participating in another study with an investigational device or an investigational drug and has not reached the primary endpoint yet.

5. CLINICAL INVESTIGATION PROCEDURES

5.1 Study Participation Status

Provisionally enrolled:

Subjects who are fully informed about the specifics of the study by authorized site personnel and provide informed consent by properly signing an informed consent form after confirmation of the initial enrolment criteria are considered as provisionally enrolled. All provisionally enrolled subjects will receive a unique study participation number.

Subjects for whom consent was not obtained prior to participation in the study will not be considered provisionally enrolled. No data collected from these “subjects” will be included in any analysis.

Screen failure:

Provisionally enrolled subjects who withdraw consent prior to the index procedure or are unsuitable for treatment with the study device following e.g. laboratory assessments, pre-procedure ECG or any study specific invasive or non-invasive treatment as pre-specified in the clinical investigation, plan are considered as screen failure. These subjects will be exited from the study once screen failure is confirmed. Enrolment data and information regarding inclusion and exclusion criteria will be entered into the EDC for screen failures subjects and their informed consent forms will be kept in the site’s administrative files.

Enrolled:

Provisionally enrolled subjects who met all eligibility criteria are considered enrolled. Enrolled subjects will be followed in accordance with the clinical investigation plan.

Study exit:

Early termination of study participation applicable to subjects who have signed an informed consent form. It can be due to:

- Subject death
- Subject lost to follow up

- Subject withdrawal of informed consent
- Subject is considered as a screen failure
- Non-implanted subject, after the 1 month follow-up

Study completion: Subject who completes all protocol-required study procedures and follow ups.

5.2 Enrollment and Procedures

The enrollment and follow up process is outlined in the Figure 1 below.

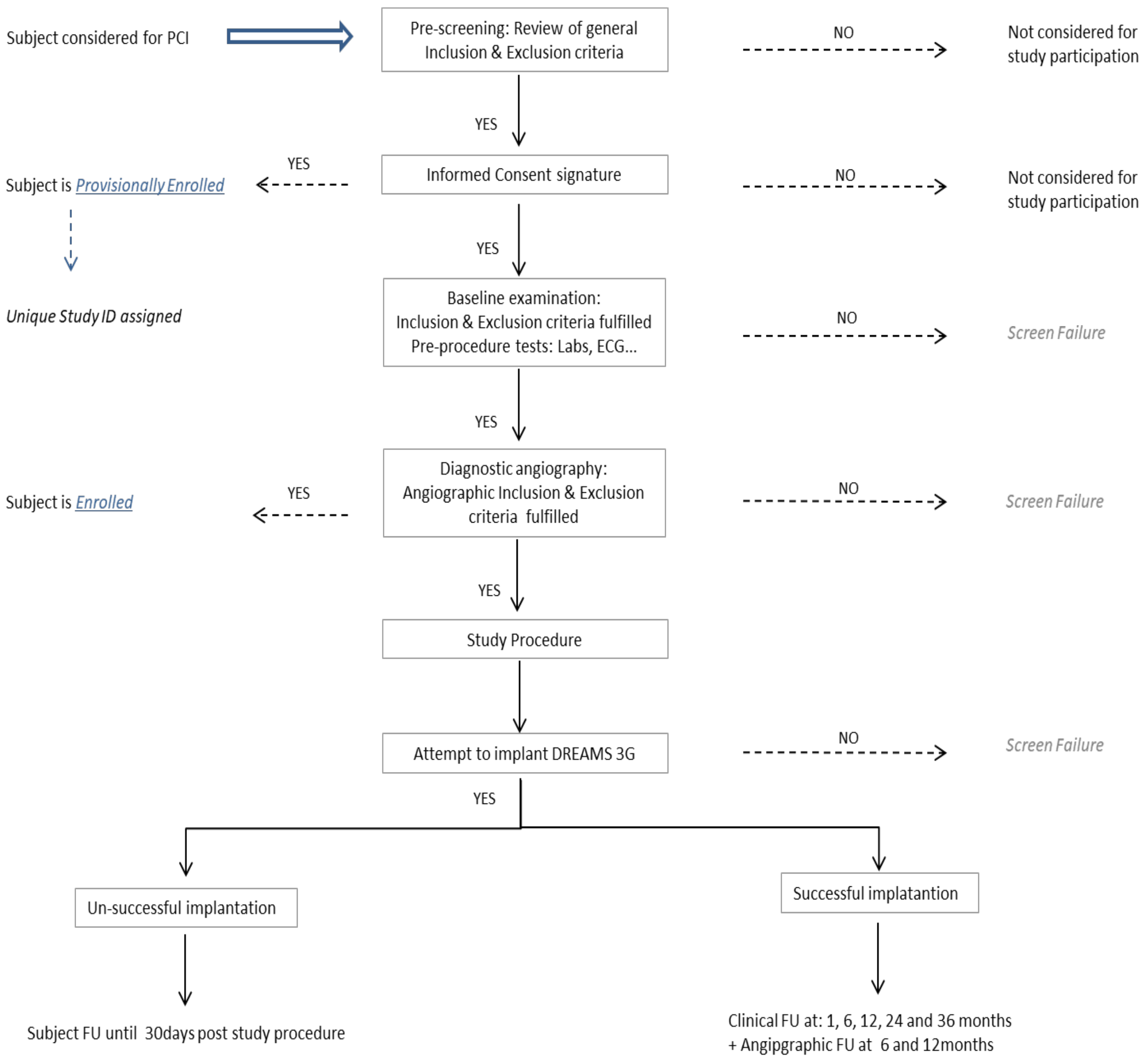

5.3 Pre-screening

Subjects being considered for percutaneous coronary interventions should be pre-screened via a general review of inclusion/exclusion criteria without any study assessment. After the subject signed the approved informed consent, study specific screening assessments can be started.

5.4 Informed Consent Process

Informed consent should be obtained in accordance with the applicable regulations and current versions of Declaration of Helsinki, ICH-GCP and ISO 14155. Information to a potential subject shall be given in a prior interview in a layperson vocabulary and in the country's main language(s) with a member of the investigating team who is appropriately qualified under national law.

Informed consent shall be written, dated (including time of consent) and signed by the person performing the interview and by the subject himself or herself. During the consent process, the background of the proposed study, the benefits and risks and inconveniences of study participation should be explained in detail to the subject. Furthermore the conditions under which the clinical investigation is to be conducted, including the expected duration of the subject's participation, have to be elucidated.

It has to be emphasized that a subject's participation in the trial is voluntary and that the subject may refuse to participate or withdraw from the trial, at any time, without penalty or loss of benefits to which the subject is otherwise entitled. The possible alternative treatments, including follow-up measures if the participation of the subject is discontinued have to be explained. More details about the content of the patient information are kept in the Informed Consent Form.

The subject must be given ample time to read the subject information and to address questions before signing the consent form. The subject information and a copy of the EC approved signed consent form (by investigator and subject) will be handed out to the subject.

Subjects are considered *provisionally enrolled* in the study once they signed the informed consent signature form. A unique study identification number is then assigned to them by the EDC system. Provisionally enrolled subjects who do not fulfill the angiographic in- and exclusion criteria or who withdraw informed consent prior to the

study procedure are screening failure and need to be documented in the EDC (inclusion and exclusion criteria page, termination page).

5.5 Enrollment

Provisionally enrolled subjects are considered *enrolled* in the clinical investigation only after fulfilling the angiographic in- and exclusion criteria, assessed by diagnostic angiography before the index procedure.
The Intention to Treat (ITT) and the Per Protocol (PP) population will only be based on enrolled subjects.
All target lesions must be treated in one procedure. Planned staged procedures are not allowed within the BIOMAG-I study.
Only DREAMS 3G may be implanted during the index procedure unless a different treatment is required in case of an emergency situation.

If there is an attempt to implant the DREAMS 3G and the study device enters the guiding catheter, but the implantation is unsuccessful (e.g: target lesion not crossed) and a non-study device is implanted, the subjects will be followed up to 1 month.
Those subjects will only be considered for baseline characteristics analysis and device and procedure success evaluation and will be excluded from all angiographic and clinical endpoint analysis.
There is no restriction in the enrolment of subjects per site and enrolment will end once up to 115 subjects have been included in the study.

5.6 Baseline Examinations

The subjects' clinical investigation eligibility must be confirmed prior to the intervention through the following tests and examinations:

- Demographics
- Review of in- and exclusion criteria
- Physical examination and relevant medical history including angina status or myocardial ischemia
- Within 72 hours prior to study procedure:
    - o For women of child-bearing potential < 55 years, a pregnancy test must be performed
    - o Serum creatinine assessment
- Within 24 hours prior to study procedure:
    - Cardiac biomarkers CK, CK-MB and Troponin (assessment of CK-MB is mandatory)
    - A 12-lead electrocardiogram

5.7 Medication guidelines

Following medication regimen is recommended throughout the clinical investigation.

Table 4: Overview medication guideline

Timing	Medication	Procedure
**Prior to Procedure**	Heparin	Left to the discretion of the cardiologist
	ASA	75 mg for 3 days prior to procedure or a peri-procedural dose of 250-500mg at the discretion of the cardiologist.
	Clopidogrel	Loading dose of 300- 600 mg < 6 hours prior or during the intervention<br>Subjects on Clopidogrel therapy (at least 7 days): no loading dose required
**During Procedure**	IV LMWH / Unfractionated Heparin	Per routine hospital practice. It is recommended to maintain an ACT > 250 seconds
	IIb/IIIa Inhibitor	Left to the discretion of the cardiologist
	Intracoronary Nitroglycerin or,	100-200 µg prior to baseline and post intervention angiograms

Timing	Medication	Procedure
	Intracoronary Isosorbide Dinitrate	1-3 mg prior to baseline and post intervention angiograms
Post-Procedure	IIb/IIIa Inhibitor	Per routine hospital practice
	ASA	75 to 320 mg per day
	Clopidogrel[1]	75 to 150 mg per day
Discharge	ASA	75 to 330 mg per day, lifelong
	Clopidogrel[1]	75 to 150 mg per day for a minimum of 6 months unless contraindicated

[1]Cardiologist may substitute to Prasugrel, Ticlopidine or Ticagrelor for subjects who are allergic, hypersensitive or resistant to Clopidogrel. Subjects on Ticlopidine must have CBC(s) performed per instructions for use.

Recommendation: If a subject requires OAC after DREAMS 3G implantation, DAPT should be maintained until 6 months follow up. Afterwards DAPT can be downsized to either ASA or Clopidogrel alone together with OAC for the remaining time period up to 12 months. After this, OAC monotherapy can be prescribed if still required.

5.8 Baseline Angiography

Following intracoronary injection of nitroglycerin or isosorbide dinitrate, a baseline angiography of the target vessel must be performed in at least 2 orthogonal views, presenting the target lesion free of foreshortening or vessel overlap. In addition to the clinical routine, OCT and IVUS including IVUS VH documentation should also be done pre-procedure. Please see the Core lab angiographic OCT, and IVUS acquisition guidelines for more details.

5.9 Index Procedure

All study investigators and site personnel involved in the clinical trial have to sign the Site Signature and Responsibility log and provide signed and dated curriculum vitae (CV).

Implanting physicians and study investigators will be thoroughly trained by the study sponsor on the study protocol, the device and procedures prior to clinical investigation procedure.

The number of implanting physicians will be limited to investigators who are trained on the CIB, the consensus paper (75), the "4 P" program of BIOTRONIK AG and the instructions for use.

Subject preparation and percutaneous access should be performed according to the standard hospital practice.

The procedure begins once percutaneous access has been made.

5.9.1.Lesion Access and Pre-dilatation

Lesion access is up to the discretion of the investigator.

Pre-dilatation of the target lesion is mandatory. A 6F guiding catheter or larger should be used. The use of drug coated balloons or rotational atherectomy device is NOT allowed.

The scaffold system is compatible with most guidewires. The target lesion should be crossed with a 0.014" guide wire.

- Pre-dilatation with a non-compliant balloon with a 1:1 balloon-to-artery ratio is mandatory.
- The balloon should expand fully.
- The residual stenosis before DREAMS 3G implantation is required to be ≤ 20%, confirmed by any method.

5.9.2. Scaffold Implantation

The scaffold implantation and sizing should be performed in accordance with the IFU. The scaffold length should cover the target lesion from normal reference vessel diameter (RVD) proximal to normal RVD distal to assure full coverage of the lesion.

Device Diameter:

2.5 mm: RVD 2.5 - 2.7mm

3.0 mm: RVD 2.7 - 3.2mm

3.5 mm: RVD 3.2 - 3.7mm

4.0 mm: RVD 3.7 - 4.2mm

Device Length:

13 mm: Lesion Length ≤11mm

22 mm: Lesion Length ≤20mm

30 mm: Lesion Length ≤28mm

Only one clinical investigation scaffold should be used per lesion.

After scaffold implantation an angiography must be performed. Following intracoronary injection of nitroglycerine or isosorbide dinitrate, angiograms should be made in the exact same 2 orthogonal views as pre-scaffolding using the same catheter type and size.

5.9.3. Post-dilatation

Post-dilatation with a non-compliant balloon of up to 0.5 mm bigger than the nominal scaffold size and at high pressure (>16 atm) is mandatory.

- DREAMS 3G expansion limit is 0.6 mm beyond nominal scaffold size.
- The use of cutting/scoring or drug coated balloons is NOT allowed.

After post-dilatation, all subjects will undergo the routine angiography as well as additional IVUS (including IVUS VH documentation) and OCT assessment. The angiographic, IVUS and OCT acquisition post-procedure is described in the corelab guidelines.

5.10 Treatment Failure and Bailout Situation

For dissections occurring during, or immediately after implantation of DREAMS 3G, a second DREAMS 3G may be used, not overlapping with initial one but implanted end-to-end.

If another stent needs to be implanted in the immediate vicinity of an already implanted DREAMS 3G , e.g., clinically significant residual stenosis or restenosis, up to 120 days post procedure, only a DREAMS 3G coronary scaffold system should be used.

Contact of the DREAMS 3G with an uncoated or polymer coated stent up to 120 days may result in accelerated resorption of the DREAMS 3G and undesired local and downstream effects. After 120 days any other stent type may be used.

The treatment with an additional stent or scaffold must be documented angiographically, and by IVUS and OCT images in following the same process described in the previous treatment section.
When a second device needs to be implanted, the "4Ps" need to be observed.

5.11 Post Procedure until Discharge

Between end of procedure and hospital discharge, following examinations have to be performed:

- A 12-lead ECG within 6-24 hours post-procedure or at discharge, whichever comes first.
- Cardiac biomarkers CK, CK-MB and Troponin between 6-24 hours post-procedure or at discharge, whichever comes first (assessment of CK-MB is mandatory)

If CK, CKMB and/or Troponin elevation is noted post-procedure, CK, CK-MB and Troponin measurements should be performed every 8 hours and documented in the CRF until values have returned to normal or until discharge, starting from when the first elevation is noted.

Note: Every effort must be made to obtain cardiac biomarkers values within the specified time ranges. Results of all cardiac biomarkers measurements, even measurements performed outside the time range must be documented on the CRFs.

Adverse events and device effects, concomitant medications, and any interventional treatment that occurred since the procedure must be recorded before discharge.

A patient implantation card must be given to the patient prior to discharge. The card documents that the patient has received a DREAMS 3G device with the corresponding

device information. The patient must be instructed to carry the patient implantation card at all times with him.

5.12 Follow-Up Visits

All enrolled subjects will be followed through hospital discharge and will undergo follow-up evaluations at following time points:

Follow-up visit	Visit window
1 month	30 days ± 7 days
6 months	180 days ± 30 days
12 months	365 days ± 30 days
24 months	730 days ± 30 days
36 months	1095 days ± 30 days

5.12.1 One Month Clinical Follow-Up

All subjects will be contacted for a clinical evaluation at 30 days post-procedure (± 7 days), including:

- Assessment of the ischemic/angina status (CCS, Braunwald or silent ischemia),
- Any adverse- or serious adverse events or device deficiencies
- Anti-platelet/anti-coagulant medical therapy

The visit can be performed by phone or as in hospital visit.

5.12.2 6 Months Clinical Follow-Up and Imaging follow-up

All subjects will be contacted for clinical and imaging evaluation at 180 days post-procedure (± 30 days), including:

- Assessment of the ischemic/angina status (CCS, Braunwald or silent ischemia),
- Any adverse- or serious adverse events or device deficiencies
- Anti-platelet/anti-coagulant medical therapy
- Angiography, IVUS (including IVUS-VH documentation) and OCT. These assessments will be done in addition to the clinical routine.
- ECG (according to hospital's standard of care)

For women of child-bearing potential < 55 years, a pregnancy test must be performed before the 6-month angiographic examination.

5.12.3 12 Months Clinical Follow-Up and Imaging follow-up

All subjects will be contacted for clinical and imaging evaluation at 365 days post-procedure (± 30 days), including:

- Assessment of the ischemic/angina status (CCS, Braunwald or silent ischemia),
- Any adverse- or serious adverse events or device deficiencies
- Anti-platelet/anti-coagulant medical therapy
- Angiography, IVUS (including IVUS-VH documentation) and OCT. These assessments will be done in addition to the clinical routine.
- Vasomotion will be assessed angiographically with Acetylcholine followed by Nitroglycerine, if subject consents. This is in addition to the clinical routine.
- ECG (according to hospital's standard of care)

For women of child-bearing potential < 55 years, a pregnancy test must be performed before the 12-month angiographic examination.

5.12.4 24 and 36 Months Clinical Follow-Up

All subjects will be contacted yearly for clinical evaluation at:

730 days post-procedure (± 30 days) and

1095 days post-procedure (± 30 days)

The following assessment will be performed:

- Ischemic/angina status (CCS, Braunwald or silent ischemia),

- Any adverse- or serious adverse events or device deficiencies
- Anti-platelet/anti-coagulant medical therapy

The visits can be performed by phone or as in hospital visit.

5.12.5 Vasomotion

If subject consents, a vasomotion test will be performed at 12 months follow up in order to obtain additional information on vasoreactivity of the vessel as well as vessel movement throughout absorption of the DREAMS 3G.

All anti-anginal agents that influence vasomotor tone, including long-acting nitrate, calcium-channel blockers, and beta-blockers, should be withheld for at least 48 h before coronary angiography, except for sublingual nitroglycerin as needed.

A coronary angiography will be taken prior to injection of acetylcholine (ACH) in the same two orthogonal views used during baseline procedure.

Diagnostic left / right heart catheterization and coronary angiography will be performed by a standard percutaneous approach. A 6-F diagnostic catheter will be introduced into the left main or right coronary artery, depending on the vessel studied. To ensure that the segments were fully bathed by the infusion of acetylcholine (ACH), the tip of the infusion catheter will be placed 8 -12 mm proximal to the proximal border of the scaffolded segment. To avoid wire induced coronary spasm, the wire will be removed.

Vasomotor reactivity will be estimated by infusing incremental doses of ACH into the coronary artery using an infusion catheter (i.e. Finecross MG; Terumo).

ACH should be admitted in different concentration.

- ACH1 (low dose): 0.36 µg/mL
- ACH2 (middle dose): 3.6 µg/mL
- ACH3 (high dose): 18 µg/mL

Speed of infusion should be 2 mL/min. Duration of infusion should be around 3 minutes. Contrast injection should be filmed after 3 minutes of i.c. infusion of ACH.

When the maximum dose is reached, an intracoronary bolus injection of nitroglycerin (200 µg) will be administered followed immediately by angiography in the same two orthogonal views used during baseline procedure.

Throughout each infusion, the heart rate, systemic arterial pressure, and ECG will be monitored continuously

End-diastolic images for each segment will be chosen and analyzed with the automated edge detection program by QCA. The scaffolded segment and the 5-mm proximal and distal to the site of scaffolding will be studied. Changes in coronary diameter in response to ACH and nitrate coronary infusion will be expressed as percent changes versus baseline angiograms. The mean lumen diameters will be measured by QCA

5.12.6 Angiographic, IVUS and OCT Follow Up

Several studies have confirmed the bias of increased TLR events introduced by protocol catheterization (120,121). With this comes the dilemma of whether subjects would have remained stable for a long time without further revascularization or would soon have become symptomatic if revascularization had not been performed. Although attempts have been made to stratify TLR driven by protocol catheterization, even independent adjudication is very complex in this setting (122).
Thus, the Academic Research Consortium (ARC) consensus for DES evaluation is to primarily assess clinically driven TLR within a time interval that precedes any protocol-mandated repeat catheterization and include subsequent TLR in secondary analyses, with best adjudication as to clinical need (123). Thus every effort should be made, to conduct the clinical evaluation prior to the angiographic evaluation.

5.12.7 Unscheduled Clinical Follow-up

If a subject comes for an unscheduled visit with the need of a (re-)intervention (e.g. based on present symptoms, diagnostic measures) the following assessments should be done **prior** to the (re-)intervention:

- Documentation of adverse events which might have occurred since last visit and the actual status of the adverse events
- Documentation on concomitant medication in case medication changed since last visit and the actual status of the concomitant medication.

- Ischemia should be documented by ECG, stress ECG, cardiac biomarkers or fractional flow reserve (FFR).
- Assessment of the ischemic/angina status (CCS, Braunwald or silent ischemia).

Unscheduled Angiographic follow-up

Any unplanned angiography and revascularization must be followed by a full clinical evaluation. The required data are to be entered into the CRF. All films must be sent to the angiographic core laboratory for review.

- If subjects undergo a clinically driven angiography within 3 or 9 months post procedure, having no re-intervention of the target lesion(s) they must return for the angiographic follow up visit at 6 and respectively, 12 months post procedure. Subjects shall not have the IVUS or the OCT examination during a clinically driven angiography within 3 months post procedure

- If subjects undergo a clinically driven angiography between 3-6 months post procedure (or at 9-12 months, respectively), this will be considered as the 6 months, respectively 12 months follow up angiography. IVUS and the OCT examination shall be done during the clinically driven angiography between 3-6 months or 9-12 months post procedure.

- If subjects have a re-intervention for in-scaffold restenosis at any time prior to the 6 months or prior to the 12 months follow up visit, they will not have an additional angiography at the 6 months or at the 12 months follow up visit respectively. This also applies for treatment of an acute or sub-acute thrombotic occlusion of the target lesion(s). Subjects should have the IVUS and the OCT examination during the time of re-intervention.

- However, if a subject has two target vessels treated with the study scaffold, whereof only one had a re-intervention, they must return for the repeat angiography at the 6 months and 12 months follow up visit. IVUS and the OCT examination shall be done during the repeat angiography.

5.13 Withdrawals / Study Exit

Early termination of study participation may apply for a study subject in any of the following situations:

- Screening failure
- Withdrawal of informed consent
- Not implanted, after the 1 month visit
- Lost to follow-up
- Death

The subject is free to withdraw its written informed consent at any time. The reason for withdrawal/discontinuation should, if available, be documented in the subjects' medical file and in the eCRF. When a subject withdraws or is withdrawn from the clinical investigation, a final clinical evaluation should be performed, documented in the medical record and in the eCRF.

A subject is not considered lost to follow up until the full 3 years have elapsed. The investigator should try to contact the subject at every follow-up until clinical investigation ends. At least 3 phone attempts and 1 attempt by registered mail should be performed for each missed visit. These attempts should be documented in the subject's medical records.

5.14 Study completion / Termination

Subject will terminate study participation when all protocol-required study procedures and follow-ups have been completed.

6. ADVERSE EVENTS

During the course of this clinical investigation adverse events might occur. For all (provisionally) enrolled subjects, adverse event information will be collected throughout the clinical investigation in the eCRF by the investigator or designee. Any adverse event will be followed until study end.

6.1 Adverse Event Definition and Classification

Adverse Events are defined and classified in this section according to the current version of ISO 14155.

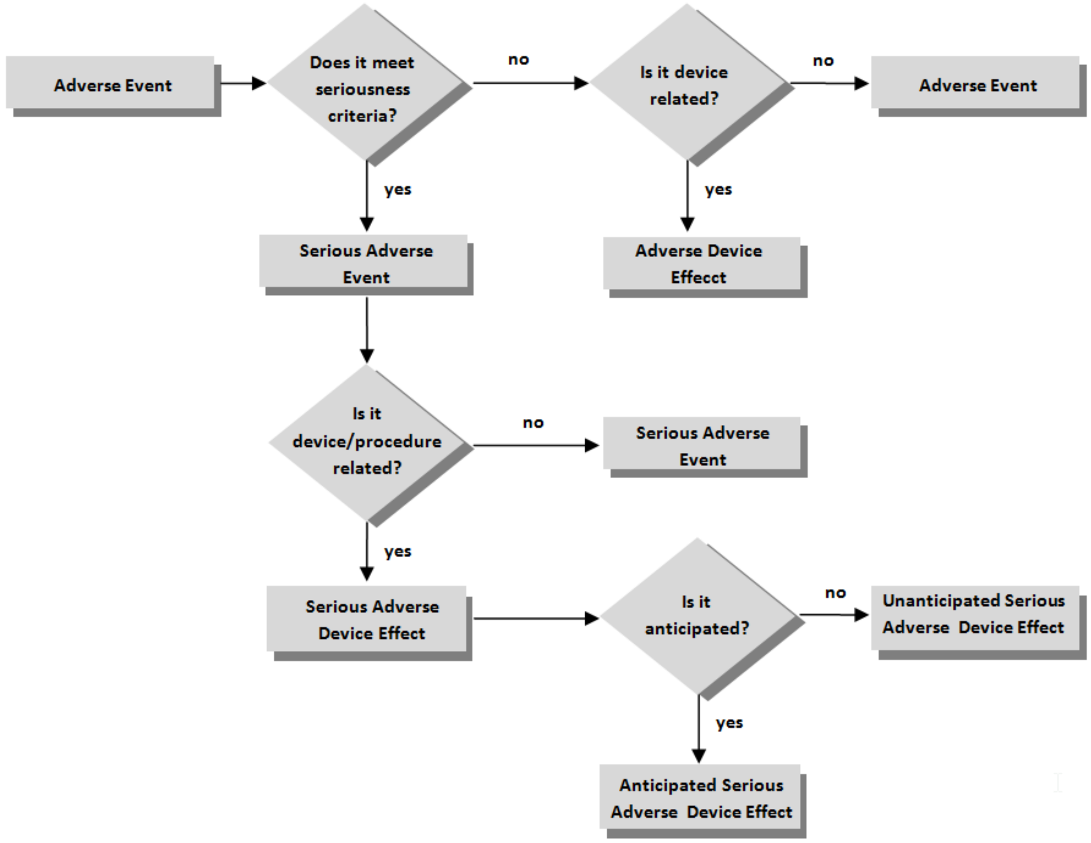

Figure 1: Adverse Event classification

AE – Adverse Event:

ISO 14155: any untoward medical occurrence, unintended disease or injury, or untoward clinical signs (including abnormal laboratory findings) in subjects, users or other persons, whether or not related to the investigational medical device.

Please note: Any current condition that is recorded as a pre-existing condition either in the medical history of physical examination section, is not an AE unless there is a change in nature, severity, or degree of incidence.

ADE – Adverse Device Effect:

ISO 14155: *adverse event* related to the use of an investigational medical device

- This definition includes adverse events resulting from insufficient or inadequate instructions for use, deployment, implantation, installation, or operation, or any malfunction of the investigational medical device.
- This definition includes any event resulting from use error or from intentional misuse of the investigational medical device.

SAE – Serious Adverse Event:

ISO 14155: *adverse event* that

a) led to death,

b) led to serious deterioration in the health of the subjects, that either resulted in

    1) a life-threatening illness or injury, or

    2) a permanent impairment of a body structure or a body function, including chronic disease or

    3) in-subject or prolonged hospitalization, or

    4) medical or surgical intervention to prevent life-threatening illness or injury or permanent impairment to a body structure or a body function,

c) led to foetal distress, foetal death or a congenital abnormality or birth defect

Please note: Planned hospitalization for a pre-existing condition, or a procedure required by the CIP, without serious deterioration in health, is not considered a serious adverse event.

SADE – Serious Adverse Device Effect:

ISO 14155: *adverse device effect* that has resulted in any of the consequences characteristic of a serious adverse event.

USADE – Unanticipated Serious Device Effect:

ISO 14155: Unanticipated adverse device effect means any serious adverse device effect which by its nature, incidence, severity or outcome has not been identified in the current version of the risk analysis report.

Device Deficiencies

ISO14155: Inadequacy of a medical device with respect to its identity, quality, reliability, safety or performance. Device deficiencies include malfunctions, use errors and inadequate labelling.

Serious Device Deficiencies

Serious device deficiencies are device deficiencies that turned out to be serious because

- suitable action had not been taken;
- intervention had not been made; or
- if circumstances had been less fortunate.

Adverse Event Reporting

Adverse event reporting will start once the patient is provisionally enrolled. Only events related to any study specific invasive procedures (e.g. blood draw) will be reported for this study population. For subjects considered fully enrolled inthe study, all events will be reported.

According to ISO14155, the investigator shall:

- Record every AE and observed device deficiency, together with an assessment
- Report to the sponsor, without unjustified delay, all SAEs and device deficiencies that could have led to an SADE; this information shall be promptly followed by detailed written reports
- Return the device in case of a device deficiency to the sponsor for further asssements.

- Report to the institutional review board (IRB)/independent ethic committee (IEC) SAEs and device deficiencies that could have led to an SADE, if required by national regulations or IRB/IEC
- Report to regulatory authorities SAE and device deficiencies that could have led to an SADE, as required by national regulations.
- Supply the sponsor, upon sponsor's request, with any additional information related to the safety reporting of a particular event.

Adverse events classified to be “serious” and/or with a relation to the investigational device have to be reported to BIOTRONIK as soon as possible after awareness by the investigator or by the investigator's designee without any unjustified delay.

Reporting should be done using the "Adverse Event or Device Deficiency Form" in the CRF. This report should deliver any information available at the time of reporting. Effort has to be made to collect as much information as possible during initial reporting. Follow-up information has to be delivered as fast as possible to ensure timely completion of the report.

The investigator is obliged to provide copies of blinded source documents from the patients' medical records, for all SAEs and ADEs, as requested by BIOTRONIK, as well as any additional information required in relation to the event. Angiographic films and technical worksheets must be sent to BIOTRONIK.

According to MEDDEV 2.7/3 revision 3, May 2015, the Sponsor shall:

Report to the Notified Competent Authorities (NCAs) where the clinical investigation has commenced:

- for all reportable events as described in **MEDDEV 2.7/3 revision 3,** section 4 which indicate an imminent risk of death, serious injury, or serious illness and that requires prompt remedial action for other patients/subjects, users or other persons or a new finding to it: immediately, but not later than 2 calendar days after awareness by sponsor of a new reportable event or of new information in relation with an already reported event.

- any other reportable events as described in **MEDDEV 2.7/3 revision 3,** section 4 or a new finding/update to it: immediately, but not later than 7 calendar days following the date of awareness by the sponsor of the new reportable event or of new information in relation with an already reported event.

<u>Primary contact for reporting Serious Adverse Events:</u>

Safety Reporting

BIOTRONIK AG

Vascular Intervention
Ackerstrasse 6, CH-8180 Buelach, Switzerland
Phone: +41 44 864 5867
Fax: +41 44 864 3130
sae.vi@biotronik.com

7. STATISTICAL ANALYSIS

This chapter gives an overview on the planned analysis of the data. The final description of analysis will be laid down in a Statistical Analysis Plan (SAP) prior to analysis of the data.

7.1 Study Populations

Primary and secondary endpoint analysis will be conducted on the following pre-defined analysis populations:

- Intent-to-treat population (primary analysis)
  - Subjects for whom an investigational scaffold enters the guide catheter following the diagnostic angiography, regardless of whether the investigational scaffold is implanted in the intended location or not.
  - Subjects who received an investigational scaffold and who are later determined not to meet study eligibility criteria or who have CIP violations.

  Note: Subjects, for whom DREAMS 3G entered the guiding catheter following the diagnostic angiography but who are not implanted (e.g. a different device had to be implanted) will be followed up until 1 month. Only baseline characteristics, device and procedure success, and safety up to 1 month will be reported for them.

- Per protocol population
  - Subjects implanted with the investigational scaffold at the intended location and who do not deviate from any relevant eligibility criteria and complete the study follow-up requirements without any relevant CIP violation.

7.2 Determination of Sample Size

The BIOMAG-I trial will assess non-inferiority of the 6-month in-scaffold late lumen loss for the DREAMS 3G scaffold vs. in-scaffold late lumen loss of a historical control from clinical trials with PLLA scaffolds as well as metallic scaffolds.

1-Primary endpoint in-scaffold late lumen loss at 6-month:

A weighted mean of 0.29mm for in-scaffold late lumen loss and a weighted pooled SD of 0.34mm were calculated based on below 6-month data from listed clinical trials.

	**In-scaffold LLL (mm) @ 6 months Mean (SD)**	**N**	**Publication**
**ABSORB**	0.44(0.35)	26	Ormiston 2008 (46)
**ABSORB**	0.19(0.18)	41	Serruys 2010 (37)
**Desolve**	0.19(0.19)	14	Verheye 2014 (94)
**Magmaris**	0.44(0.36)	113	Haude 2016 (11)
**Desolve**	0.20(0.32)	113	Abizaid 2016 (70)
**Fantom II**	0.25(0.4)	100	Chevalier 2019 (102)

For the DREAMS 3G the in-scaffold late lumen loss is thus estimated to be 0.29 mm.

The null and alternative hypotheses for non-inferiority testing of the primary endpoint are formulated as the following:

$$H_0: \mu_1 \geq \mu_{01} + \Delta$$
$$H_a: \mu_1 < \mu_{01} + \Delta$$

Here, μ_1 is the mean in-scaffold LLL of DREAMS 3G at 6 months after index procedure, μ_{01} is the historical control value derived as the weighted mean of in-scaffold LLL at 6

months obtained from the literature review ($\mu_{01} = 0.29\, mm$), and Δ is the prespecified non-inferiority margin (Δ= $0.145\, mm$).

Substituting the absolute values, the hypotheses can be simplified to the following:

$$H_0: \mu_1 \geq 0.435\, mm$$
$$H_a: \mu_1 < 0.435\, mm$$

The sample size calculation is made with:

Power:	0.95
Alpha:	0.025
NIM (Non-Inferiority Margin):	0.145
S (Standard Deviation):	0.34
Dropout rate:	15%

Rejection of the null hypothesis will mean that in-scaffold LLL of DREAMS 3G is non inferior to the historical control at 6-month. A total of 88 subjects enrolled (74 subjects plus 15% dropout) will have 95% power to reject the above null hypothesis in favor of the alternative assumptions.

Secondary endpoint in-scaffold late lumen loss at 12-month:

A weighted mean of 0.33 mm for in-scaffold late lumen loss and a weighted pooled SD of 0.35 mm were calculated based on the 12 month results of below listed clinical trials.

Scaffold	In scaffold LLL (mm) @ 12 months Mean (SD)	N	Publication
**Absorb**	0.27 (0.32)	56	Serruys 2011 (38)
**Magmaris**	0.39 (0.34)	99	Haude 2018 (21)
**Fantom II**	0.29 (0.36)	31	Gomez Lara 2011 (124)
**Mirage Vs. Absorb**	0.37 (IQR: 0.08 to 0.72)<br>0.23 (IQR: 0.15 to 0.37)	35<br>27	Tenekecioglu 2017 (125)

For the DREAMS 3G the in-scaffold late lumen loss is estimated to be 0.33 mm.

The null and alternative hypotheses for non-inferiority testing of the powered secondary are formulated as the following:

$$H_0: \mu_2 \geq \mu_{02} + \Delta$$
$$H_a: \mu_2 < \mu_{02} + \Delta$$

Here, μ_2 is the mean in-scaffold LLL of DREAMS 3G at 12 months after index procedure, μ_{02} is the historical control value derived as the weighted mean of in-scaffold LLL at 12 months obtained from the literature review ($\mu_{02} = 0.33\,mm$), and Δ is the prespecified non-inferiority margin (Δ= $0.145\,mm$).

Substituting the absolute values, the hypotheses can be simplified to the following:

$$H_0: \mu_2 \geq 0.475\,mm$$
$$H_a: \mu_2 < 0.475\,mm$$

The sample size calculation is made with:

Power:	0.95
Alpha:	0.025
NIM (Non-Inferiority Margin):	0.145
S (Standard Deviation):	0.35
Dropout rate:	25%

Rejection of the null hypothesis will mean that in-scaffold LLL of DREAMS 3G is non inferior to the historical control at 12-month. A total of 104 subjects enrolled (78 subjects plus 25% dropout) will have 95% power to reject the above null hypothesis in favor of the alternative assumptions.

Conclusion: as the study is powered for both endpoints at 6 and 12-month, a total of up to 104 subjects need to be enrolled in order to prove non-inferiority of the DREAMS 3G scaffold vs. historical control devices. Increasing the number of enrolled subjects beyond the sample size requirement of evaluable subjects, in order to account for potential losses to follow-up will result in a total number of up to 115 patients to be enrolled.

Sample size was calculated using PASS 15 and planning for a one sample t-test.

7.3 Analysis Methods

Descriptive statistics will be presented for all baseline, procedure and FUP data, as well as device and procedure success, and angiographic endpoints.

For quantitative variables, the mean values, standard deviation, median, maximum and minimum, as well as the 95% CI (confidence interval) for the mean will be calculated. For qualitative variables, absolute and relative frequencies are determined, and the exact 95% confidence interval for proportions is calculated when relevant.

Adverse event rates will refer to the respective time intervals (e.g. 365 +/- 30 days).

Hypothesis testing will be performed for the primary endpoint using one sample one-sided non-inferiority t-test, see section 7.2.

For all clinical secondary endpoints and their components (TLF, cardiac death, all-cause mortality, TV MI, CD TLR, CD TVR, Definite and probable scaffold thrombosis) the survival rate (and 95% CI) will be calculated using Kaplan-Meier estimator. Kaplan-Meier survival curves will also be presented.

7.4 Missing Data

All possible steps will be taken to minimize missing data in the study, including monitoring of data forms for completeness and efforts to track and maintain contact with study subjects during the follow-up period.
Unless otherwise noted, denominators for computation of percentages exclude subjects with missing data.

7.5 Interim Analysis and Final Report

An interim analysis is planned after all subjects have reached the primary endpoint at 6 months. Another analysis will be performed after all subjects have reached the 12 months follow up visit. Afterwards, annual interim reports will be issued upon request. The final report will be issued after 3 years follow up.

7.6 Subgroup analysis

Subgroup analyses may be performed ad hoc for exploratory purposes.

8. QUALITY CONTROL AND QUALITY ASSURANCE

8.1 Data Monitoring Committee

8.1.1. Data Safety Monitoring Board

An independent Data Safety Monitoring Board (DSMB) will have the responsibility to assess in intervals, the progress of the clinical investigation, the safety data or the critical performance endpoints and to recommend the sponsor, whether to continue, suspend, modify or stop the clinical investigation.

Its conduct will be governed by a written DSMB charter describing its rules of operation and responsibilities.

8.1.2. Clinical Events Committee

BIOTRONIK will instruct the centres to compile blinded source documents with the necessary data for the Clinical Events Committee (CEC). The CEC will meet depending on the event rate to review and adjudicate AEs, ADEs, SAEs, SADEs and USADEs. The decision of the committee is based on independent physician review of the eCRF and supporting source documentation, as required. All events will be supported by clinical data from the subjects' medical records. Classification of cause and circumstances surrounding the event will occur upon agreement of the reviewers. The classification system used and procedures implemented will be summarized in a CEC charter which will document the definitions and adjudication procedure.

Final adjudication of events will be done by the CEC. The adjudication by the CEC will overrule the one of the investigator, should the investigator and CEC disagree.

8.2 Core Laboratory

A core laboratory (MedStar Health Reearch Institute, Maryland, USA) will be used for the independent assessment of image findings (angiographies, IVUS and OCT). Standard measurement process will be designed by the independent core laboratory and specified in written core laboratory guidelines. Each of the selected investigative sites will receive training on the acquisition, interpretation and transmission of images.

The images should be stored and sent according to core laboratory guidelines.

Any additional (unscheduled) imaging data should be collected and sent to the core laboratory as well.

8.3 Data Collection / Data MonitoringData Collection

The investigator or an individual designated by the investigator is responsible for recording all data from the study in the electronic Case Report Form (eCRF).

The data will be entered in an electronic data capture system (EDC), compliant with the General Data Proection Regulation. Data should be entered within 5 days of the visit

and have to be done in English. Sites with incomplete or outstanding eCRFs may be prohibited from enrolment until data entry is complete.

All site staff must be trained on correct completion of the eCRF, before they are given access for data entry. All CRF pages must be electronically signed by the investigator or a person designated by the investigator at each site.

Subjects will only be identified in the eCRFs by a unique reference number, a composition of letters and numbers. eCRFs are confidential documents and will only be available to BIOTRONIK (including BIOTRONIK designees), the investigator, the clinical investigation statistician, and the DMC and regulatory authorities. The investigator or a person designated by the investigator will maintain as part of the clinical investigation file a subject's identification log of the subjects participating in the clinical investigation at the clinical investigation site.

An appropriate internet access is required for data entry and upload of pseudo-anonymised source documents.

8.3.2 Monitoring

Monitoring will be performed by BIOTRONIK or by BIOTRONIK designees, to ensure that the investigator and the clinical investigation team conduct the clinical investigation in accordance with the CIP, current versions of Declaration of Helsinki, ISO 14155, International conference of harmonization-Good clinical practice (ICH-GCP) and applicable regulations to ensure adequate protection of the rights, safety and wellbeing of subjects and the quality and integrity of the resulting data.

Periodic monitoring visits will assure that the facilities are still acceptable; that the CIP is being followed, that the IEC/IRB has been informed about approved CIP changes as required, that records on study conduct and data collection are complete and present, that appropriate and timely reports have been made to the sponsor and the authorities, that device inventory is controlled, and that the investigator is carrying out all agreed activities.

During monitoring visits, 100 % source data verification as defined in the monitoring plan will be performed. For this purpose, the investigator must permit access to medical records of the subjects (source data) throughout the study. In case of

electronic medical records the investigator has to ensure that the monitor receives appropriate access or print outs of all study relevant source documents will be made (signed and dated by the investigator or other designated staff).

If a monitor becomes aware that an investigator is not complying with the requirements as outlined in the CIP, the monitor is obliged to notify BIOTRONIK study management. BIOTRONIK will evaluate the non-compliance and issue corrective actions, discontinue enrollment or as a last measure close the clinical investigation site. The investigator must in such case return all unused devices to BIOTRONIK.

8.4 Source Data

Original or signed and dated copies of all clinical findings, observations, and other activities throughout the clinical investigation must be recorded and maintained in the medical file of each enrolled subject. For data documented in the eCRF respective source data in the medical record (source document) must be available.

The medical record/subjects record (source document) must contain, but is not limited to:

- Executed informed consent
- Subjects participation in the clinical investigation
- Demographics
- Documentation of medical history
- Intervention report
- Vessel and lesion sizes and characteristics
- All angiographies, ECGs, laboratory examination outcomes including reference values
- Discharge letter
- All adverse events: diagnosis, symptoms, onset date, severity, device and procedure relationship, action taken, outcome
- Device deficiencies: including the assessment by the investigator whether the device deficiency is in connection with a malfunction, use error, inadequate labelling and whether the device deficiency is considered serious. Concomitant medication

- Follow-up information
- Date of clinical investigation completion

8.5 Data Management

The data manager is responsible for setting up an EDC system, its validation and maintenance during study duration. Queries (manual or pre-defined checks) are set up in the EDC system and should be resolved by the investigator or a person designated by the investigator in a timely manner. The data manger provides a clean data set at the end of the clinical investigation. Data snapshots will be performed for interim analysis. When all data is complete, the database will be locked and data analyzed.

8.6 Protocol Compliance

The investigator is required to conduct the study in accordance with the signed study agreement and clinical protocol.

The investigator shall notify BIOTRONIK and the reviewing IRB/IEC in writing, no later than 2 days after any significant violations from the study plan, conducted to protect the life or physical well-being of a subject in an emergency. Except in such emergency, prior approval by BIOTRONIK and, if applicable, IRB/IEC approval is required for significant violations from the study plan. Such an approval will be documented in writing and maintained in the study files.

BIOTRONIK categorizes instances of protocol non-compliance as either violations or deviations.

8.6.1 Protocol Violations

Protocol violations are defined as instances where the protocol requirements and/or regulatory guidelines were not followed and are generally more serious in nature. Protocol violations are considered to potentially affect the scientific soundness of the study and/or the rights, safety or welfare of subjects.

Protocol violations include, but are not limited to:

- Failure to obtain informed consent

- An unapproved (BIOTRONIK and IRB/IEC) investigator implanting an investigational device for study purposes
- Violation of relevant inclusion/exclusion criteria
- Protocol requirement violations that affect the primary endpoints analysis

In some instances, compliance issues with the consent process may occur. The investigator should seek guidance from the site's IRB/IEC to ensure the subject received appropriate information to consider their participation in the study. The investigator is obliged to take any action the IRB/IEC feels is necessary, including subjects removal from the study. Please note that subjects who haven't signed informed consent prior to implantation will not be included in the study analysis, even if informed consent could be obtained retrospectively.

All violations will be reported to the competent authority in accordance with applicable regulatory timelines. The study site should report the protocol violation to the reviewing IRB/IEC as applicable and provide a copy of the notification to BIOTRONIK. The site should also report the protocol violation to BIOTRONIK on the applicable CRF.

8.6.2 Protocol Deviations

Protocol deviations are defined as instances where protocol requirements are not followed in such a manner whereby data is unusable or unavailable. Protocol deviations are less serious in nature and do not require IRB/IEC notification, as long as they do not have an effect on the rights, safety or welfare of the study subjects.

Protocol deviations include, but are not limited to:

- Procedure not performed within the allowed follow-up window
- Required data not obtained
- Follow-up procedure performed at an unapproved location

The study site should report the protocol deviation on the applicable eCRF.

8.7 Audits / Inspections

Study centers may be audited during the course of and after completion of the clinical investigation by BIOTRONIK or BIOTRONIK designees, IRB/IEC, competent authority or other applicable regulatory authorities.

The investigator must provide the auditor with all clinical investigation documents including the medical records for all enrolled subjects.
BIOTRONIK will evaluate any non-compliance and issue corrective actions, discontinue enrolment or at last measure, close the clinical investigation site, if monitoring or auditing identifies serious or repeated deviations on the part of an investigator.

9. REGULATORY REQUIREMENTS

9.1 Compliance Statement

This clinical investigation will be contributed in compliance with the applicable regulations for conduction of clinical investigations with human beings, especially with regard to:

- The Declaration of Helsinki current version (Fortaleza version 2013, active at protocol signature)
- ISO 14155 current version (version 2011, active at protocol signature) Clinical investigation of medical devices for human subjects - Good clinical practice[38]
- Medical Device Directive 93/42/EEC
- ICH/GCP current version (version 2002, active at protocol signature)
- All applicable local and national requirements in the participating countries (including safety reporting, e.g. “Verfahren schwerwiegende unerwünschte Ereignisse in Deutschland” dated 06Dec2019 and “Safety reporting procedures Switzerland” dated 10Jan2020).

9.2 IRB/IEC and Regulatory Bodies

The clinical investigation plan as well as other relevant study documents required will be submitted for approval by the IRB/IEC and competent authority prior to study start as required by local and national regulations.

BIOTRONIK will record changes to the clinical investigation plan in the revision history. Amendments will be submitted to the involved IRB/IEC and respective competent authorities as applicable according to national or local regulations. All study personnel will be trained on all amendments as required.

All subjects must be provided with a written informed consent form which is approved by the site's IRB/IEC. Each site must provide BIOTRONIK AG with a copy of the clinical site's IEC/IRB approval letter or vote and the IRB/IEC approved informed consent. In addition the composition of the IRB/IEC and a statement about compliance to GCP is required. Approvals for the continuation of the trial at each clinical site must be kept current and notifications forwarded to BIOTRONIK.

9.3 Insurance

Subjects who participate in this study will be insured for study related injury according to local regulatory requirements. BIOTRONIK will organize appropriate insurance coverage which will be available throughout the entire study.

9.4 Subject data protection

Subject's protection of personal data will be maintained throughout the study. It has to be ensured that all data collected can be tracked back to the source data, if required. For this purpose, a unique subject identification code (i.e. subject number) will be used that allows identification of all data reported for each subject.
Data relating to the study might be made available to third parties (e.g. in case of an audit performed by regulatory authorities) preconditioned the data are treated confidentially and that each subject's privacy is guaranteed.

9.5 Records

Records to be maintained by the investigator include, but are not limited to:

- Clinical trial investigational plan and all amendments
- Investigators Brochure and amendments
- Contact list sponsor/study site
- Signed clinical trial agreement

- IRB/IEC correspondence, approval letter, including informed consent
- IRB/IEC membership list
- Regulatory authority notification, correspondence and approval
- Insurance certificate
- If applicable, device shipping records, device accountability
- Correspondence relating to the trial
- CVs for all investigators and key members of the investigation site team
- Training records
- Financial Disclosure Forms for all investigators
- Site personnel signature list and delegation of authority form
- Monitoring visit log
- Blank set of CRFs and instructions for completion
- Subjects screening/enrollment log
- Subjects identification log
- Lab certification and lab test normal ranges
- Adverse event forms
- Reports (including interim reports, final reports and safety reports)

BIOTRONIK will retain relevant study documentation in the Trial Master File (TMF) according to international legislation and BIOTRONIK Standard Operating Procedure (SOPs).

9.6 Record Retention

All study records and reports will remain on file at the sites for a minimum of 10 years after completion of the trial and will further be retained in accordance with local and international guidelines as identified in the clinical study agreement.

The investigator must contact BIOTRONIK before destruction of any records and reports related to the clinical investigation. BIOTRONIK must be informed if the investigator plans to leave the clinical investigation site. In such case the site must

name a new contact person before the investigator parts from the clinical investigation site.

9.7 Study Termination

BIOTRONIK will monitor the progression of the clinical investigation. If warranted, the clinical investigation may be suspended or discontinued early if there is an observation of serious adverse reactions presenting an unreasonable risk to the clinical investigation population.

The following stopping rules were set up by the data safety monitoring board. As the enrolment duration is planned for up to 5 months, only events during the first 30 days of each patient's individual follow-up time will be considered for the stopping rules.

In general, the monitoring method expressed in the stopping rules are based on the application of the sequential ratio probability test (126). For the different event types a postulated value, an upper limit, a fixed alpha error of 5% and a beta error of 20% were used. If the number of events is equal or higher than the numbers of events in the left column of the tables at the moment when the number of patients in the right column is recruited, a DSMB meeting will be held in order for the members to review and to vote whether a potential suspension of patient enrolment must be considered.

Cardiac death, target vessel related MI, and scaffold thrombosis are relevant for patient's safety. All events are rare. In the comparable BIOSOLVE II and BIOSOLVE III (studies testing the Magmaris device, see also section 1.1.3), one patient was affected by these events.

Using the point estimator of 0.8% (BIOSOLVE III 30 days event rate of the combination cardiac death, target vessel related MI, and scaffold thrombosis) as the postulated value, and 4.6% (BIOSOLVE III upper 95% confidence limit for estimation of combined event rate) as an upper limit, the resulting stopping rules are as follows:

Number of event	Patients included
2	20
3	66
4	112

In addition, the 30 days event rate of clinical driven TLR should be monitored. Using 0.1% as postulated value (BIOSOLVE III no event) and 4.6% as an upper limit, the resulting stopping rules are as follows:

Number of event	Patients included
1	23
2	107

BIOTRONIK may also terminate investigator and site participation in the clinical investigation if there is evidence of failure to maintain adequate clinical standards, failure to comply with the clinical investigational plan, fraud or any other forms of misconduct.

In the event of clinical investigation termination or suspension, BIOTRONIK will send a report outlining the circumstances to the corresponding IRB/IEC, regulatory body and all investigators. A suspended or terminated clinical investigation may not be re-initiated without approval of the corresponding IRB/IEC and competent authority.

9.8 Investigator Reimbursement and Contracting

BIOTRONIK will reimburse efforts undertaken for inclusion and follow up of subjects, and documentation of subject data within the study. A contract with the principal investigator and/or the respective hospital will be agreed on and signed prior to study start. Within this an overall fee per subjects broken down to individual visits will be included.
This subject fee will cover all expenses for material used and procedures to be performed according to the CIP. The CIP and any future changes thereof will be part of the contract.

10. PUBLICATION POLICY

BIOTRONIK intends to publish the results of this clinical investigation. BIOTRONIK reserves the right to include the report of this clinical investigation in any regulatory

documentation or submission or in any informational materials prepared for the medical profession. The ownership of the data shall at all times be held by BIOTRONIK.

Within one year after study completion a final report shall be submitted to the Member States, signed by the participating investigators of the clinical investigation. No publication of results from single center experience will be allowed until the primary endpoint is analyzed in order to allow for preparation and publication of the multicenter results.

BIOTRONIK agrees that investigators shall be permitted to present at symposia, national or regional professional meetings, and to publish in journals, thesis or dissertations, or otherwise of their own choosing, methods and results of the clinical investigation after the first publication. Any prior publication in any way or form is not permitted, without approval by BIOTRONIK.

The investigator should provide BIOTRONIK with a copy of any publication in journals, thesis or dissertations at least 4 weeks prior to submission.

11. APPENDICES

Appendix 1: Abbreviations

Appendix 2: Definitions

Appendix 3: “4 P” STRATEGY

APPENDIX 1: Abbreviations

ACH	Acetylcholine
ADE	Adverse Device Effect
AE	Adverse Event
AMI	Acute Myocardial Infarction
AMS	Absorbable Metal Scaffold
ASA	Acetyl Salicylic Acid
BVS	Bioabsorbable Vascular Scaffold
BRS	Bio-Resorbable Scaffold
CA	Competent Authority
CABG	Coronary Artery Bypass Graft
CI	Confidence Interval
CIB	Clinical Investigator’s Brochure
CIP	Clinical Investigation Plan
CCS	Canadian Cardiovascular Society
CEC	Clinical Event Committee
CRA	Clinical Research Associate
CRF	Case Report Form
cTn	Cardiac troponin
DAPT	Dual-Antiplatlet Therapy
DES	Drug-Eluting Stent
DS	Diameter Stenosis
DSMB	Data Safety Monitoring Board
DREAMS	Sirolimus-Eluting Resorbable Coronary Magnesium Scaffold System
ECG	ElectroCardioGram
FFR	Fractional Flow Reserve
FIM	First in Men (clinical study)
FUP	Follow UP
GCP	Good Clinical Practice
ICH	International Conference on Harmonization
IFU	Instructions For Use
IRB/IEC	Institutional Review Board/Independent Ethics Committee
ISO	International Standard Organisation

ITT	Intention To Treat
IVUS	IntraVascular UltraSound
LLL	Late Lumen Loss
LST	Late Stent Thrombosis
MI	Myocardial Infarction
MLD	Minimal Luminal Diameter
OAC	Oral Anticoagulation Therapy
OCT	Optical Coherence Tomography
PCI	Percutaneous Coronary Intervention
PLLA	Poly-L-Lactid Acid
PTCA	Percutaneous Transluminal Coronary Angioplasty
QCA	Quantitative Coronary Angiography
RMS	Resorbable Magnesium Scaffold
RVD	Reference Vessel Diameter
SADE	Serious Adverse Device Effect
SAE	Serious Adverse Event
SOP	Standard Operating Procedure
ST	Stent Thrombosis
TIA	Transient Ischemic Attack
TIMI	Thrombolysis In Myocardial Infarction
TLF	Target Lesion Failure
TLR	Target Lesion Revascularization
TVF	Target Vessel Failure
TVR	Target Vessel Revascularization
ULN	Upper Limit Normal
URL	Upper Reference Limit
US	United States
USADE	Unanticipated Serious Adverse Device Effect
VLST	Very Late Stent Thrombosis

APPENDIX 2: Definitions

ACUTE GAIN

Acute gain was defined as the immediate dimensional change in minimal luminal diameter (in mm) that occurred after the final post-dilatation as compared to the minimal luminal diameter at baseline and measured by quantitative coronary angiography from the average of 2 orthogonal views.

BRAUNWALD CLASSIFICATION (127)

**Severity**		**Clinical circumstances in which unstable angina occurs**		
		**A**	**B**	**C**
		Develops in Presence of Extra cardiac Condition That Intensifies Myocardial Ischemia (Secondary Unstable Angina)	Develops in Absence of Extra cardiac Condition (Primary Unstable Angina)	Develops Within 2 wk of AMI (Post infarction Unstable Angina)
**I**	New onset of severe angina or accelerated angina; no rest pain	IA	IB	IC
**II**	Angina at rest within past month but not within preceding 48 h	IIA	IIB	IIC

	(angina at rest, sub-acute)			
**III**	Angina at rest within 48 h (angina at rest, acute)	IIIA	IIIB-Troponin$_{neg}$ IIIB-Troponin$_{pos}$	IIIC

CARDIOGENIC SHOCK (CS) (128)

Cardiogenic shock (CS) is a state of end-organ hypoperfusion due to cardiac failure. The definition of CS includes hemodynamic parameters: persistent hypotension (systolic blood pressure < 80 to 90 mm Hg or mean arterial pressure 30 mm Hg lower than baseline) with severe reduction in cardiac index (< 1.8 L x min^{-1} x m^{-2} without support or < 2.0 to 2.2 L x min^{-1} x m^{-2} with support) and adequate or elevated filling pressure (eg,
left ventricular end-diastolic pressure > 18 mm Hg or right ventricular end-diastolic pressure > 10 to 15 mm Hg)

CANADIAN CARDIOVASCULAR SOCIETY (CCS) ANGINA CLASSIFICATION (129,130)

Class	Definition
I	Ordinary physical activity does not cause angina such as walking, climbing stairs. Angina (occurs) with strenuous, rapid, or prolonged exertion at work or recreation.
II	Slight limitation of ordinary activity. Angina occurs on walking or climbing stairs rapidly, walking uphill; walking or stair climbing after meals, in cold, in wind, or under emotional stress, or only during the

	few hours after awakening;, Angina occurs on walking more than 2 blocks on the level and climbing more than one flight of ordinary stairs at a normal pace and in normal conditions.
III	Marked limitation of ordinary physical activity. Angina occurs on walking one to two blocks on the level and climbing one flight of stairs in normal conditions and at a normal pace.
IV	Inability to carry on any physical activity without discomfort - angina symptoms may be present at rest.

DE NOVO LESION

A native coronary artery lesion not previously treated.

DEATH ACCORDING TO ARC-1 DEFINITION (131)

Cardiac death	Any death due to proximate cardiac cause (e.g., MI, low-output failure, fatal arrhythmia), unwitnessed death and death of unknown cause and all procedure-related deaths, including those related to concomitant treatment, will be classified as cardiac death.
Vascular death	Death caused by non-coronary vascular causes, such as cerebrovascular disease, pulmonary embolism, ruptured aortic aneurysm, dissecting aneurysm, or other vascular diseases.
Non-cardiovascular	Any death not covered by the above definitions, such

death	as death caused by infection, malignancy, sepsis, pulmonary causes, accident, suicide, or trauma.
All deaths are considered cardiac unless an unequivocal non-cardiac cause can be established. Specifically, any unexpected death even in subjects with coexisting potentially fatal non-cardiac disease (e.g., cancer, infection) should be classified as cardiac.	

DEATH ACCORDING TO ARC-2 DEFINITION (132)

Type of death	Definition
Cardiovascular	Cardiovascular death as defined as death resulting from cardiovascular causes. The following categories may be collected:<br>1) Death caused by acute MI<br>2) Death caused by sudden cardiac, including unwitnessed death<br>3) Death resulting from heart failure<br>4) Death caused by stroke<br>5) Death caused by cardiovascular procedure<br>6) Death resulting from cardiovascular haemorrhage<br>7) Death resulting from other cardiovascular causes
Non-cardiovascular death	Non-cardiovascular death is defined as any death that is not thought to be the result of a cardiovascular cause. The following categories may be collected:<br>1) Death resulting from malignancy<br>2) Death resulting from pulmonary cause<br>3) Death caused by infection (including sepsis)<br>4) Death resulting from gastrointestinal causes<br>5) Death resulting from accident/trauma<br>6) Death caused by non-cardiovascular organ failure<br>7) Death resulting from other non-cardiovascular causes

Undetermined	Undetermined cause of death is defined as a death non attributable for any other category because of the absence of any relevant source documents. Such deaths will be classified as cardiovascular for endpoint determination.

DEVICE SUCCESS

Device Success is defined as a final residual diameter stenosis of <30% by QCA, using the assigned device only

- successful delivery of the scaffold to the target lesion site in the coronary artery
- appropriate scaffold deployment
- successful removal of the device
- safe removal of the device in case of deployment failure

The definition is based on the EU guideline on medical devices from 2015 (MeDDev 2.7.1 Appendix 1).

DEVICE AND /OR PROCEDURE CAUSALITY ASSESSMENT

The following definitions will be used to assess the relationship of the adverse event to the investigational medical device or to the study procedures:

1) Not related: relationship to the device or procedures can be excluded when:

- the event is not a known side effect of the product category the device belongs to or of similar devices and procedures;
- the event has no temporal relationship with the use of the investigational device or the procedures;
- the serious event does not follow a known response pattern to the medical device (if the response pattern is previously known) and is biologically implausible;
- the discontinuation of medical device application or the reduction of the level of activation/exposure - when clinically feasible – and reintroduction of its use (or increase of the level of activation/exposure), do not impact on the serious event;
- the event involves a body-site or an organ not expected to be affected by the device or procedure;
- the serious event can be attributed to another cause (e.g. an underlying or concurrent illness/ clinical condition, an effect of another device, drug, treatment

or other risk factors);

- the event does not depend on a false result given by the investigational device used for diagnosis, when applicable;
- harms to the subject are not clearly due to use error;
- In order to establish the non-relatedness, not all the criteria listed above might be met at the same time, depending on the type of device/procedures and the serious event.

2) Unlikely: the relationship with the use of the device seems not relevant and/or the event can be reasonably explained by another cause, but additional information may be obtained.

3) Possible: the relationship with the use of the investigational device is weak but cannot be ruled out completely. Alternative causes are also possible (e.g. an underlying or concurrent illness/ clinical condition or/and an effect of another device, drug or treatment). Cases were relatedness cannot be assessed or no information has been obtained should also be classified as possible.

4) Probable: the relationship with the use of the investigational device seems relevant and/or the event cannot be reasonably explained by another cause, but additional information may be obtained.

5) Causal relationship: the serious event is associated with the investigational device or with procedures beyond reasonable doubt when:

- the event is a known side effect of the product category the device belongs to or of similar devices and procedures;
- the event has a temporal relationship with investigational device use/application or procedures;
- the event involves a body-site or organ that
  - o the investigational device or procedures are applied to;
  - o the investigational device or procedures have an effect on;
- the serious event follows a known response pattern to the medical device (if the response pattern is previously known);
- the discontinuation of medical device application (or reduction of the level of

activation/exposure) and reintroduction of its use (or increase of the level of activation/exposure), impact on the serious event (when clinically feasible);

- other possible causes (e.g. an underlying or concurrent illness/ clinical condition or/and an effect of another device, drug or treatment) have been adequately ruled out;
- harm to the subject is due to error in use;
- the event depends on a false result given by the investigational device used for diagnosis, when applicable;
- In order to establish the relatedness, not all the criteria listed above might be met at the same time, depending on the type of device/procedures and the serious event.

PERCENT DIAMETER STENOSIS

Mean lumen diameter divided by the reference vessel diameter times one hundred

DISTAL EMBOLIZATION

Distal embolization is defined as free-flowing blood clot or lesion material that was located in the coronary circulation distal to the treated lesion.

ELIGBLE SUBJECTSS

Enrolled subjects that fulfils all inclusion and none of the exclusion criteria

EVALUABLE SUBJECTS

Evaluable subjects are subjects of intended for imaging analysis where imaging quality is sufficient to perform a full analysis.

IN-SCAFFOLD

- In-scaffold is defined as proximal edge to distal edge of the implanted scaffold

IN-SEGMENT

- In-segment is defined as in-scaffold plus 5 mm distal and 5 mm proximal

INTENT-TO-TREAT

The intention-to-treat (ITT) population includes all subjects in whom scaffold implantation is attempted (i.e. the clinical investigation scaffold has entered the guiding

catheter). In case a different device needs to be implanted and the patient did not receive a DREAMS 3G scaffold, the patient will be excluded from all angiographic and clinical endpoint analysis. Those patients will be included for device and procedure success analysis only.

LATE LUMEN LOSS (LLL)

Post-procedure minimal lumen diameter (MLD) minus follow-up MLD as determined by quantitative angiography.

LESION CLASSIFICATION, ACC/AHA (133)

(American College of Cardiology/American Heart Association)

Type	Definition
A	Discrete (< 10 mm length); Concentric; Readily accessible; Non-angulated segment, < 45°; smooth contour; little or no calcification; less than totally occlusive; Not ostial in location; no major branch involvement; absence of thrombus
B1	One of the following adverse characteristics: Tubular (10 – 20 mm length); Eccentric, moderate tortuosity of proximal segment; moderately angulated segment, > 45 - < 90; irregular contour; moderate – heavy calcification; total occlusion < 3 months old; ostial in location; bifurcation lesions requiring double guide wires; some thrombus present
B2	≥ two of the following adverse characteristics: Tubular (10 – 20 mm length); Eccentric, moderate tortuosity of proximal segment; moderately angulated segment, > 45 - < 90; irregular contour; moderate – heavy calcification; total occlusion < 3 months old; ostial in location; bifurcation lesions requiring double guide wires; some thrombus present
C	Diffuse (> 2 cm length); excessive tortuosity of proximal segment;

	extremely angulated segments > 90°; total occlusion > 3 months old; inability to protect major side branches; degenerated vein grafts with friable lesions

MAJOR BLEEDING COMPLICATIONS

All pseudo aneurysms, vascular access site bleeding associated with a decrease in haemoglobin ≥3.0 mmol/L as well as vascular events which required surgical repair or transfusion of > 2 units within 30 days of the procedure.

MAJOR VASCULAR COMPLICATIONS

Haemorrhagic vascular complications included the following:

1. Haematoma at access site >5 cm
2. False aneurysm
3. AV fistula
4. Retroperitoneal bleed
5. Peripheral ischemia/nerve injury
6. Any transfusion required will be reported as a vascular complication unless clinical indication clearly other than catheterization complication
7. Vascular surgical repair

MINIMAL LUMINAL DIAMETER (MLD)

Mean minimum lumen diameter derived from two orthogonal views (by the quantitative coronary angiography laboratory)

MULTIVESSEL DISEASE

The presence of a greater than 50% diameter stenosis as measured by quantitative coronary angiography on-line in 2 or 3 major epicardial coronary vessels or bypassed branches.

MYOCARDIAL INFARCTION (MI)

Myocardial infarction will be adjudicated according to the 3rd (134,135) and 4th (136) universal definition of myocardial infarction, on the basis of the 2010 ARC extended historical definition (137) of myocardial infarction, of ARC-2 (132) and using the consideration of the SCAI (138) for a definition of Clinically Relevant Myocardial

Infarction After Coronary Revascularization.

Definition of Clinically Relevant Myocardial Infarction After Coronary Revascularization according to SCAI (138)**:**

1- In patient with normal baseline CK-MB:

- The peak CK-MB measured within 48 hours of the procedure rises to ≥10x the local laboratory upper limit normal (ULN), or to ≥5x ULN with new pathologic Q waves in ≥2 contiguous leads or new persistent LBBB, OR
- In the absence of CK-MB measurement and a normal baseline cTn, a cTn (I or T) level measured within 48 hours of the PCI rises to ≥70x of the local laboratory ULN, or

  ≥35x ULN with new pathologic Q waves in ≥2 contiguous leads or new persistent LBBB,

2-In patient with elevated baseline CK-MB (or cTn) in whom the biomarker levels are stable or falling:

The CK-MB or (cTn) rises by an absolute increment equal to those levels recommended above from the most recent pre-procedure level.

3 In patient with elevated CK-MB (or cTn) in whom the biomarker levels have not been shown to be stable or falling:

The CK-MB or (cTn) rises by an absolute increment equal to those levels recommended above, plus new ST-segment elevation or depression, plus signs consistent with a clinically relevant MI, such as new onset or worsening heart failure or sustained hypotension.

Definition of Clinically Relevant Myocardial Infarction After Coronary Revascularization according to the Academic Research Consortium-2 (ARC-2) (132):

Myocardial infarction

- Absolute rise in cardiac troponin (from baseline) > 35 times URL
- Plus one or more of the criterion below:
  - o New significant Q-waves or equivalent*
  - o Flow limiting angiographic complications

  - New “substantial” loss of myocardium on imaging

Significant periprocedural myocardial injury:

- Absolute rise in cardiac troponin (from baseline) ≥ 70 times URL

* Q-wave criteria requires the development of new Q waves ≥40 ms in duration and ≥1 mm deep in voltage in ≥2 contiguous leads.

3rd Universal definition of myocardial infarction (134,135): The term acute myocardial infarction should be used when there is evidence of myocardial necrosis in a clinical setting consistent with acute myocardial ischemia. Under these conditions any one of the following criteria meets the diagnosis for myocardial infarction:

- Detection of rise and/or fall of cardiac biomarkers values [preferably cardiac troponin (cTn)] with at least one value above the 99th percentile of the upper reference limit (URL) and with at least one of the following:
  - → Symptoms of ischemia;
  - → New or presumed new significant ST-segment–T wave (ST–T) changes or new left bundle branch block (LBBB).
  - → Development of pathological Q waves in the ECG;
  - → Imaging evidence of new loss of viable myocardium or new regional wall motion abnormality
  - → Identification of an intracoronary thrombus by angiography or autopsy.
- Cardiac death with symptoms suggestive of myocardial ischaemia and presumed new ischaemic ECG changes or new LBBB, but death occurred before cardiac biomarkers were obtained, or before cardiac biomarker values would be increased.
- Percutaneous coronary intervention (PCI) related MI is arbitrarily defined by elevation of cTn values (>5 x 99th percentile URL) in patients with normal baseline values (≤99th percentile URL) or a rise of cTn values >20% if the baseline values are elevated and are stable or falling. In addition, either
  - → (i) symptoms suggestive of myocardial ischaemia or
  - → (ii) new ischaemic ECG changes or
  - → (iii) angiographic findings consistent with a procedural complication or
  - → (iv) imaging demonstration of new loss of viable myocardium or new regional wall motion abnormality are required.

- Stent thrombosis associated with MI when detected by coronary angiography or autopsy in the setting of myocardial ischaemia and with a rise and/or fall of cardiac biomarker values with at least one value above the 99th percentile URL.
- Coronary artery bypass grafting (CABG) related MI is arbitrarily defined by elevation of cardiac biomarker values (>10 x 99th percentile URL) in patients with normal baseline cTn values (≤99th percentile URL). In addition, either
  - → (i) new pathological Q waves or new LBBB, or
  - → (ii) angiographic documented new graft or new native coronary artery occlusion, or
  - → (iii) imaging evidence of new loss of viable myocardium or new regional wall motion abnormality.

3rd Universal Classification of Myocardial Infarction (134,135)

Classification	Description
Type 1 Spontaneous myocardial infarction	Spontaneous myocardial infarction related to atherosclerotic plaque rupture, ulceration, fissuring, erosion, or dissection with resulting intraluminal thrombus in one or more of the coronary arteries leading to decreased myocardial blood flow or distal platelet emboli with ensuing myocyte necrosis. The patient may have underlying severe CAD but on occasion non-obstructive or no CAD.
Type 2 Myocardial infarction secondary to an ischaemic imbalance	In instances of myocardial injury with necrosis where a condition other than CAD contributes to an imbalance between myocardial oxygen supply and/or demand, e.g. coronary endothelial dysfunction, coronary artery spasm, coronary embolism, tachy-/brady-arrhythmias, anaemia, respiratory failure, hypotension and hypertension with or without LVH.

Classification	Description
Type 3<br>Myocardial infarction resulting in death when biomarker values are unavailable	Cardiac death with symptoms suggestive of myocardial ischaemia and presumed new ischaemic ECG changes or new LBBB, but death occurring before blood samples could be obtained, before cardiac biomarker could rise, or in rare cases cardiac biomarkers were not collected.
Type 4a<br>Myocardial infarction related to percutaneous coronary intervention (PCI)	Myocardial infarction associated with PCI is arbitrarily defined by elevation of cTn values >5 x 99th percentile URL in patients with normal baseline values (≤99th percentile URL) or a rise of cTn values >20% if the baseline values are elevated and are stable or falling. In addition, either<br>(i) symptoms suggestive of myocardial ischaemia, or<br>(ii) (ii) new ischaemic ECG changes or new LBBB, or<br>(iii) (iii) angiographic loss of patency of a major coronary artery or a side branch or persistent slow or no-flow or embolization, or<br>(iv) (iv) imaging demonstration of new loss of viable myocardium or new regional wall motion abnormality are required.
Type 4b<br>Myocardial infarction related to stent thrombosis	Myocardial infarction associated with stent thrombosis is detected by coronary angiography or autopsy in the setting of myocardial ischaemia and with a rise and/or fall of cardiac biomarkers values with at least one value above the 99th percentile URL.

Classification	Description
Type 5<br>Myocardial infarction related to coronary artery bypass grafting (CABG)	Myocardial infarction associated with CABG is arbitrarily defined by elevation of cardiac biomarker values >10 x 99th percentile URL in patients with normal baseline cTn values (£99th percentile URL). In addition, either (i) new pathological Q waves or new LBBB, or (ii) angiographic documented new graft or new native coronary artery occlusion, or (iii) imaging evidence of new loss of viable myocardium or new regional wall motion abnormality.

4th Universal definition of myocardial infarction (136)

1-The term myocardial injury should be used when there is evidence of elevated cardiac troponin values (cTn) with at least one value above the 99th percentile URL.

The myocardial injury is considered acute if there is a rise and/or fall of cTn values.

2- Acute myocardial infarction: type 1, 2 and 3 MI.

Acute myocardial injury with clinical evidence of acute myocardial ischemia and with detection of ris and/or fall of cTn values with at least one valute above the 99th percentile URL and at least one of the following:

- → Symptoms of myocardial ischemia;
- → New ischaemic ECG changes
- → Development of pathological Q waves in the ECG;
- → Imaging evidence of new loss of viable myocardium or new regional wall motion abnormality in a pattern consistent with an ischemic aetiology
- → Identification of an coronary thrombus by angiography or autopsy.

3-Percutaneous coronary intervention (PCI) related MI ≤48 h after the index procedure: type 4 and 5 MI.

Arbitrarily defined by elevation of cTn values (>5 x 99th percentile URL for type 4a MI and >10 x 99th percentile URL for type 5 MI) in patients with normal baseline values. Patients with elevated pre-procedural cTn values, in whom the pre-procedural cTn levels are stable (≤20% variation) or falling, must meet the criteria for a >5 or >10 fold increase and manifest a change from the baseline value of > 20%. In addition

with at least one of the following:

→ New ischaemic ECG changes

→ Development of pathological Q waves in the ECG;

→ Imaging evidence of new loss of viable myocardium or new regional wall motion abnormality in a pattern consistent with an ischemic aetiology

→ Angiographic findings consistent with a procedural flow-limiting complication such as coronary dissection, occlusion of a major epicardial artery or graft: side-branch occlusion-thormbus, disruption of collateral flow or distal embolization.

4-Any of the following criteria meets the diagnosis for prior or silent/unrecognized MI:

→ Abnormal Q-waves with or without symptoms in the absence of non-ischemic causes

→ Imaging evidence of loss of viable myocardium in a pattern consistent with ischaemic aetiology

→ Patho-anatomical findings of a prior MI

4th Universal Classification of Myocardial Infarction (136)

Classification	Description
**Myocardial infarction Type 1**	Detection of a rise and/or fall of cTn values with at least one value above the 99th percentile URL and with at least one of the following:<br>— Symptoms of acute myocardial ischemia<br>— New ischemic ECG changes<br>— Development of pathological Q-waves<br>— Imaging evidence of new loss of viable myocardium or new regional wall motion abnormality in a pattern consistent with an ischemic aetiology<br>— Identification of a coronary thrombus by angiography including intracoronary imaging or by autopsy.*<br>*Post-mortem demonstration of an artherothrombus in the artery supplying the infarcted myocardium, or a macroscopically large circumscribed area of necrosis with or without intramyocardial haemorrhage, meets the type 1 MI criteria regardless of cTn values.

Classification	Description
**Myocardial infarction Type 2**	Detection of a rise and/or fall of cTn values with at least one value above the 99th percentile URL, and evidence of an imbalance between myocardial oxygen supply and demand unrelated to coronary thrombosis, requiring at least one of the following:<br>— Symptoms of acute myocardial ischemia<br>— New ischemic ECG changes<br>— Development of pathological Q-waves<br>— Imaging evidence of new loss of viable myocardium or new regional wall motion abnormality in a pattern consistent with an ischemic aetiology
**Type 3 Myocardial infarction resulting in death when biomarker values are unavailable**	Patients who suffer cardiac death with symptoms suggestive of myocardial ischemia accompanied by presumed new ischaemic ECG changes or ventricular fibrillation, but die before blood samples for biomarkers can be obtained, or before increase in cardiac biomarkers can be identified, or MI is detected by autopsy examination.
**Type 4a Myocardial infarction ≤ 48 h after percutaneous coronary intervention (PCI)**	Coronary intervention-related MI is arbitrarily defined by an elevation of cTn values more than five times the 99th percentile URL in patients with normal baseline values. In patients with elevated pre-procedure cTn in whom the cTn level are stable (≤20% variation) or falling, the post-procedure cTn must rise by >20%. However, the absolute post-procedural value must still be at least five times the 99th percentile URL. In addition, one of the following elements is required:<br>— New ischemic ECG changes<br>— Development of new pathological Q-waves*<br>— Imaging evidence of new loss of viable myocardium or new regional wall motion abnormality in a pattern

Classification	Description
	consistent with an ischemic aetiology<br>— Angiographic findings consistent with a procedural flow-limiting complication such as coronary dissection, occlusion of a major epicardial artery or a side branch occlusion/thrombus, disruption of collateral flow, or distal embolization.**<br>*Isolated development of new pathological Q-waves meets the type 4a I criteria if cTn values are elevated and rising but more than five times the 99th percentile URL.<br>**Post-mortem demonstration of a procedure-related thrombus in the culprit artery, or a macroscopically large circumscribed area necrosis with or without intra-myocardial haemorrhage meets the type 4a MI criteria.
**Type 4b Myocardial infarction related to stent/scaffold thrombosis associated with PCI**	A subcategory of PCI-related MI is stent/scaffold thrombosis, type 4b MI, as documented by angiography or autopsy using the same criteria utilized for type 1 MI. It is important to indicate the time of the occurrence of the stent/scaffold thrombosis in relation to the timing of the PCI procedure. The following temporal categories are suggested:<br>— acute 0-24 h;<br>— subacute > 24 h to 30 days;<br>— late > 30 days;<br>— very late > 1 year after stent/scaffold thrombosis.
**Type 4c Myocardial infarction related to restenosis associated with PCI**	Occasionally MI occurs and -at angiography, in-stent restenosis, or restenosis following balloon angioplasty in the infarct territory- is the only angiographic explanation since no other culprit lesion or thrombus can be identified. This PCI-related MI type is designated as type 4c MI, defined as focal or diffuse restenosis, or a complex lesion associated with a rise and/or fall of cTn values above the 99th percentile URL applying, the same criteria utilized for type 1 MI.

Classification	Description
**Type 5 Myocardial infarction related to coronary artery bypass grafting (CABG); ≤ 48 h after the index procedure**	Myocardial infarction associated with CABG is arbitrarily defined as elevation of cTn values > 10 times the 99th percentile URL in patients with normal baseline cTn values. In patients with elevated pre-procedure cTn in whom cTn levels are stable (≤ 20% variation) or falling, the post-procedure cTn must rise by >20%. However, the absolute post-procedural value still must be >10 times the 99th percentile URL. In addition, one of the following elements is required:<br>— Development of new pathological Q-waves*<br>— Angiographic documented new graft occlusion or new native coronary artery occlusion<br>— Imaging evidence of new loss of viable myocardium or new regional wall motion abnormality in a pattern consistent with an ischemic aetiology<br>* Isolated development of new pathological Q-waves meets the type 5 MI if cTn values are elevated and rising but >10 times the 99th percentile URL.

Extended historical definition of myocardial infarction (137)

According to the guideline myocardial infarction following PCI (Percutaneous Coronary Intervention is defined as follows:

If Biomarkers of Myocardial Damage (CK and CKMB and Trop < 1*URL) and not acute MI in progress.

**Periprocedural <48 hours post PCI**
A. New pathologic q waves in ≥ 2 contiguous ECG leads **and**:<br>▪ any CKMB > 1*URL **or**<br>▪ in the absence of CKMB: Troponin > 1*URL **or**<br>▪ in the absence of CKMB and Troponin: CK > 1*URL **or**<br>▪ in the absence of CKMB and Troponin and CK: CEC decision upon clinical scenario

B. Appropriate cardiac enzyme data (respecting top-down hierarchy, b1 to b3):<br>b1. CK ≥ 2* URL Confirmed by :<br>▪ CKMB > 1*URL ***or***<br>▪ in the absence of CKMB:, Troponin > 1*URL ***or***<br>▪ in the absence of CKMB and Troponin: CEC decision upon clinical scenario
OR<br>b2. In the absence of CK: CKMB > 3*URL
OR<br>b3. In the absence of CK and CKMB: Troponin > 3*URL

If Baseline Biomarkers of Myocardial Damage: CK and/or CKMB > 1*URL or acute MI in progress

**Myocardial infarction, re-infarction (extension) <48 hours post PCI**
A. If CK (or CKMB) from index MI has not yet reached its maximum level:<br>▪ Recurrent thoracic chest pain or ischemia equivalent >20 minutes (or new ECG changes consistent with MI)<br>**and**<br>▪ Appropriate cardiac enzyme data:<br>o A rise in CK within 24 hours of the index event >2*URL (confirmed by either CKMB or Troponin > 1*URL) and ≥ 50% above the previous level **or**<br>o In absence of CK: a (post PCI) rise in CKMB within 24 hours of the index event >3*URL and ≥ 50% above the previous level. **or**<br>o In absence of CK and CKMB: a (post PCI) rise of Troponin within 24 hours of the index event >3*URL and ≥ 50% above the previous level.<br><br>B. If elevated CK (or CKMB) following the index MI has peaked **and** CK level has returned < URL then any new rise in:

▪ CK >2*URL(confirmed by either CKMB > URL or Troponin >URL) **or**<br>▪ in the absence of CK: CKMB > 3*URL **or**<br>▪ in the absence of CK and CKMB, Troponin > 3*URL<br><br>C. If CK (or CKMB) following the index MI has peaked **and** CK level has NOT returned<br>to < URL:<br>▪ A rise in CK ≥50% above the previous level and > 2 URL confirmed by either CKMB > URL or Troponin > URL. **or**<br>▪ In absence of CK, when CKMB has NOT returned < URL, a rise in CKMB ≥50% above the previous level and > 3 URL. ***or***<br>▪ In absence of CK, when CKMB and Troponin has not returned < URL a rise in Troponin ≥ 50% above the previous level and >3*URL

**Spontaneous MI >48 hours post PCI**
A. Recurrent thoracic chest pain or ischemic equivalent **and**<br>▪ New pathologic q waves in ≥ 2 contiguous ECG leads **and** any CKMB > 1*URL.**or**<br>▪ in the absence of CKMB: Troponin > 1*URL **or**<br>▪ in the absence of CKMB and Troponin: CK > 1*URL **or**<br>▪ in the absence of CKMB and Troponin and CK: CEC decision upon clinical scenario
B. Appropriate cardiac enzyme data (respecting top-down hierarchy):<br>b1. CK ≥ 2* URL Confirmed by:<br>▪ CKMB > 1*URL **or**<br>▪ in the absence of CKMB: Troponin > 1*URL **or**<br>▪ in the absence of CKMB and Troponin: CEC decision upon clinical scenario
**Or**<br>b2. In the absence of CK: CKMB > 3*URL
**Or**

b3. In the absence of CK and CKMB: Troponin > 3*URL
**Or**<br>b4. In the absence of CK, CK-MB and Troponin, clinical decision based upon clinical scenario.

URL = upper reference limit, defined as 99th percentile of normal reference range

Definition of Clinically Relevant Myocardial Infarction After Coronary Revascularization (138):

<u>1- In patient with normal baseline CK-MB</u>:

- The peak CK-MB measured within 48 hours of the procedure rises to ≥10x the local laboratory ULN, or to ≥5x ULN with new pathologic Q waves in ≥2 contiguous leads or new persistent LBBB, OR
- In the absence of CK-MB measurement and a normal baseline cTn, a cTn (I or T) level measured within 48 hours of the PCI rises to ≥70x le local laboratory ULN, OR
- ≥35 ULN with a new pathologic Q waves in ≥2 contiguous leads or new persistent LBBB,

<u>2-In patient with elevated baseline CK-MB (or cTn) in whom the biomarker levels are stable or falling:</u>

The CK-MB or (cTn) rises by an absolute increment equal to those levels recommended above form the most recent pre-procedure level.

<u>3 In patient with elevated CK-MB (or cTn) in whom the biomarker levels have not been shown to be stable or falling:</u>

The CK-MB or (cTn) rises by an absolute increment equal to those levels recommended above, plus a new ST-segment elevation or depression, plus signs consistent with a clinically relevant MI, such as new onset or worsening heart failure or sustained hypotension.

NYHA (New York Heart Association Classification) (139)

Class	Definition

I	Subjects with no limitation of activities; they suffer no symptoms from ordinary activities.
II	Subjects with slight, mild limitation of activity; they are comfortable with rest or with mild exertion.
III	Subjects with marked limitation of activity; they are comfortable only at rest.
IV	Subjects who should be at complete rest, confined to bed or chair; any physical activity brings on discomfort and symptoms occur at rest

OSTIAL LESION

Lesion involving the origin of the coronary artery within the first 3 mm.

PER PROTOCOL POPULATION (PPP)

Defined as all subjects from the ITT population who met all relevant inclusion and exclusion criteria and relevant treatment procedures, excluding subjects with CIP violations or without primary endpoint measured.

PROCEDURE SUCCESS

Procedure Success defined as achievement of a final diameter stenosis of <30% by QCA, using any percutaneous method, without the occurrence of cardiac death, Q-wave or non-Q-wave, or repeat revascularization of the target lesion during the hospital stay. The definition is based on the EU guideline on medical devices from 2015 (MeDDev 2.7.1 Appendix 1).

REFERENCE VESSEL DIAMETER (RVD)

The interpolated reference vessel diameter is based on a computed estimation of the original diameter of the artery at the level of the obstruction (minimal luminal diameter)

STENT (SCAFFOLD) THROMBOSIS ACCORDING TO ARC-2 DEFINITION (132)

**Timing of stent/scaffold thrombosis (duration after stent implantation)**	
Acute stent (scaffold) thrombosis	0^+ to 24 hours after stent (scaffold) implantation
Sub-acute thrombosis	> 24 hours to 30 days after stent (scaffold) implantation
Late stent (scaffold) thrombosis	> 30 days to 1 year after stent (scaffold) implantation
Very late stent (scaffold) thrombosis	> 1 year after stent (scaffold) implantation

Early stent/scaffold thrombosis is 0 to 30 days (acute plus subacute stent/scaffold thrombosis)

$^+$ Defined as the moment the patient is undraped and taken off the catherization table.

Reporting of late or very late stent thrombosis may be complex to interpret when events occur secondary to an intervening TLR, but censorship may bias reporting in favor of devices with higher restenosis risk. ARC-2 favors reporting of such events as secondary stent thrombosis. Stent thrombosis events, after BRS reintervention even years after complete scaffold resorption, should also be reported as secondary stent thrombosis.

**Classification**	**Criteria**
Definite stent/scaffold thrombosis	Angiographic confirmation of stent/scaffold thrombosis*<br>• The presence of a thrombus$^+$ that originates in the stent/scaffold or in the segment 5 mm proximal or distal to the stent/scaffold or in a side branch originating from the stented/scaffolded segment and the presence of at least 1 of the following criteria:<br>o Acute onset of ischemic symptoms at rest

	o New electrocardiographic changes suggestive of acute ischemia<br>o Typical rise and fall in cardiac biomarkers (refer to definition of spontaneous MI)<br>Or<br>• Pathological confirmation of stent/scaffold thrombosis<br>o Evidence of recent thrombus within the stent/scaffold determined at autopsy<br>o Examination of tiusse retrieved following thrombectomy (visual/histology)
Probable stent/scaffold thrombosis	Regardless of the time after the index procedure, any myocardial infarction that is related to documented acute ischemia in the territory of the implanted stent/scaffold without angiographic confirmation of stent/scaffold thrombosis and in the absence of any other obvious cause. §
Silent stent/scaffold occlusion	The incidental angiographic documentation of stent occlusion in the absence of clinical signs or symptoms is not considered stent thrombosis.

* Definite stent/scaffold thrombosis is considered to have occurred by either angiographic or pathological confirmation.

+ Occlusive thrombus: Thrombolysis in MI grade 0 or 1 flow within or proximal to a stent/scaffold segment. Non-occlusive thrombus: intracoronary thrombus is defined as a (spherical, ovoid, or irregular) noncalcified filling defect or lucency surrounded by contrast material (on 3 sides or within a coronary stenosis) seen in multiple projections, persistence of contract material within the lumen, or visible embolization of intraluminal material downstream.

§ When the stented/scaffolded segment is in the left circumflex coronary artery or in the presence of pre-existing electrocardiographic abnormalities (e.g. LBBB, paced rhythms) definitive evidence of localization may be absent and CEC adjudication is based on review of all available evidence.

STROKE

Defined as sudden onset of vertigo, numbness, dysphasia, weakness, visual field defects, dysarthria or other focal neurological deficits due to vascular lesions of the brain such as haemorrhage, embolism, thrombosis, or rupturing aneurysm, that persists > 24 hours.

TARGET LESION

Any lesion treated or attempted to be treated during an intervention.

TARGET LESION REVASCULARIZATION (122)

Target lesion revascularization is defined as a repeated revascularization, including bypass surgery, within the in-segment area

CLINICALLY DRIVEN TARGET LESION REVASCULARIZATION (TLR) (123)

Any clinically-driven repeat percutaneous intervention of the target lesion including 5mm proximal and 5mm distal from the edge of the scaffold, or bypass surgery of the target vessel that was performed for a clinical indication and was due to restenosis or closure of the target lesion.

Clinically-driven revascularizations are those in which the subjects has a positive functional clinical investigation, ischemic ECG changes at rest in a distribution consistent with the target vessel, or ischemic symptoms and an in-lesion diameter stenosis ≥ 50% by QCA. Revascularization of a target lesion with an in-lesion diameter stenosis ≥70% (by QCA) in the absence of the above-mentioned ischemic signs or symptoms is also considered clinically-driven. In the absence of QCA data for relevant follow-up angiograms, the clinical need for revascularization is adjudicated using the presence or absence of ischemic signs and symptoms.
Non-clinically driven repeat target lesion revascularizations are those in which the subjects undergoes a non-emergent revascularization for a diameter stenosis < 50% (by QCA). Non-emergent repeat target lesion revascularization for a diameter stenosis < 70% (by QCA) in subjects without either a positive functional clinical investigation or angina are also considered non-clinically driven.

TARGET LESION FAILURE (TLF) according to ARC-1 (131)

Defined as the composite of Cardiac death, Target vessel Q-wave or non-Q wave Myocardial Infarction (MI) (i.e., Q-wave MI that cannot be attributed to a non-target vessel), clinically driven Target Lesion Revascularization (TLR) and Emergent Coronary Artery Bypass Grafting (CABG).

TARGET LESION FAILURE according to ARC-2 (132)

Defined as the composite of cardiovascular death, target vessel MI and clinically driven TLR.

TARGET VESSEL

Any coronary vessel (e.g. left main coronary artery, left anterior descending coronary artery, left circumflex coronary artery, right coronary artery) containing a target lesion.

TARGET VESSEL REVASCULARIZATION (TVR) (123)

Revascularization of any segment of the index coronary artery, which was in physical contact with any component (guiding catheter, guide wire, balloon catheter, etc.) of the angioplasty hardware during the initial procedure.

TARGET VESSEL FAILURE (TVF)

Defined as composite of cardiac death, target vessel Q-wave or non-Q wave myocardial infarction, coronary artery bypass graft and clinically driven target vessel revascularization.

TIMI CLASSIFICATION (103)

A system developed during the TIMI–Thrombolysis in Myocardial Infarction trials for grading the severity of stenosis and extent of blood flow through the coronary arteries

Grade	Definition
0	No perfusion
1	Penetration with minimal perfusion. Contrast fails to opacify the entire bed distal to the stenosis for the duration of the cine run.
2	Partial perfusion. Contrast opacifies the entire coronary bed distal to the stenosis. However, the rate of entry and/or clearance is slower in the coronary bed distal to the obstruction than in comparable areas not perfused by the dilated vessel.
3	Complete perfusion. Filling and clearance of contrast equally rapid in the coronary bed distal to stenosis as in other coronary beds.

APPENDIX 3: The 4P Strategy

Fajadet J et al Eurointervention 2016;12:828-33 (75)
DREAMS 3G Instructions for Use

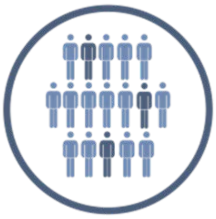

Patients and lesions selection

Appropriate patient selection is crucial to achieve procedural success.
DREAMS 3G is currently indicated for ***de novo lesions***, with a reference vessel diameter and lesion length closely matching the available DREAMS 3G sizes.

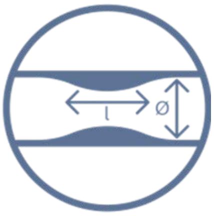

Proper sizing

If uncertain about the vessel diameter, use QCA, IVUS and/or OCT for quantitative lesion evaluation.
Available diameters are **2.5, 3.0, 3.5 and 4.0mm**

- ✓**Do not implant** into vessels diameter **<2.5 or >4.2 mm**

Available lengths are 13, 22 and 30 mm

- ✓**Do not implant** in target lesion length **≥28 mm**

Angiogram generally underestimates the diameter of the vessel by 0.25mm

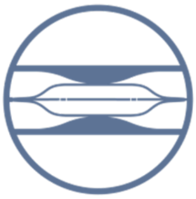

Pre-Dilatation

Pre-dilatation with a **non-compliant balloon with a 1:1 balloon-to-artery ratio** is **mandatory**.
The balloon should expand fully.
Residual stenosis before DREAMS 3G implantation is recommended to be ≤ **20 %.**
If the pre-dilatation goal is not achieved, use other balloon technologies such as scoring balloons.

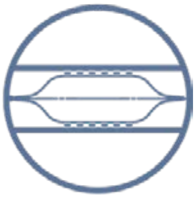

Post-Dilatation

Post-dilatation with a **non-compliant balloon 0.5mm larger** than the implanted scaffold and **expanded at high pressure** (>16 atm) is **mandatory**
DREAMS 3G expansion limit is **0.6 mm** beyond nominal scaffold size.

OCT is helpful to check for vessel and lumen dimensions, lesion length and struts' mal-apposition.

12.

*In the event of incomplete lesion coverage/dissection: a second DREAMS 3G can be placed **END TO END** with the first implanted DREAMS 3G.*

OVERLAPPING SHOULD BE AVOIDED.

12. References

1. Onuma Y, Serruys PW. Rather Thick, Yet Antithrombogenic: Is the Magmaris Scaffold a New Hope for Bioresorbable Coronary Scaffold? *Circ Cardiovasc Interv*.2017;10.
2. Bangalore S, Toklu B, Amoroso N, Fusaro M, Kumar S, Hannan EL, Faxon DP, Feit F. Bare metal stents, durable polymer drug eluting stents, and biodegradable polymer drug eluting stents for coronary artery disease: Mixed treatment comparison meta-analysis. *BMJ*.2013;347:f6625.
3. Bangalore S, Toklu B, Patel N, Feit F, Stone GW. Newer-Generation Ultrathin Strut Drug-Eluting Stents Versus Older Second-Generation Thicker Strut Drug-Eluting Stents for Coronary Artery Disease: A Meta-Analysis of Randomized Trials. *Circulation*.2018;138:2216–26.
4. Park SJ, Kang SJ, Virmani R, Nakano M, Ueda Y. In-stent neoatherosclerosis: A final common pathway of late stent failure. *JACC*.2012;59:2051–7.
5. Iqbal J, Onuma Y, Ormiston J, Abizaid A, Waksman R, Serruys P. Bioresorbable scaffolds: Rationale, current status, challenges, and future. *Eur Heart J*.2014;35:765–76.
6. Kang SH, Kang S-J, Kim W-J. Neoatherosclerosis as the Cause of Late Failure of a Bioresorbable Vascular Scaffold at 8 Months. *JACC Cardiovasc Interv*.2017.
7. Ribichini FL, Pesarini G. The promise of vascular reparative therapy in standby mode. How long before a final decision? Complete vessel wall regeneration and vascular scaffold resorption after left anterior descending reconstructions. *EuroIntervention*.2018;14:e373-e376.
8. Kereiakes DJ, Ellis SG, Metzger C, Caputo RP, Rizik DG, Teirstein PS, Litt MR, Kini A, Kabour A, Marx SO, Popma JJ, McGreevy R, Zhang Z, Simonton C, Stone GW. 3-Year Clinical Outcomes With Everolimus-Eluting Bioresorbable Coronary Scaffolds: The ABSORB III Trial. *JACC*.2017;70:2852–62.
9. Tijssen RYG, Kraak RP, Hofma SH, van der Schaaf RJ, Arkenbout K, Weevers A, Elias J, van Dongen IM, Koch KT, Baan J, Vis M, Winter RJ de, Piek JJ, Tijssen JGP, Henriques JPS, Wykrzykowska JJ. Complete two-year follow-up with formal non-inferiority testing on primary outcomes of the AIDA trial comparing the Absorb bioresorbable scaffold with the XIENCE drug-eluting metallic stent in routine PCI. *EuroIntervention*.2018.
10. Haude M, Erbel R, Erne P, Verheye S, Degen H, Böse D, Vermeersch P, Wijnbergen I, Weissman N, Prati F, Waksman R, Koolen J. Safety and performance of the drug-eluting absorbable metal scaffold (DREAMS) in patients with de-novo coronary lesions: 12 month results of the prospective, multicentre, first-in-man BIOSOLVE-I trial. *Lancet*.2013;381:836–44.

11. Haude M, Ince H, Abizaid A, Toelg R, Lemos PA, Birgelen C von, Christiansen EH, Wijns W, Neumann FJ, Kaiser C, Eeckhout E, Lim ST, Escaned J, Onuma Y, Garcia-Garcia HM, Waksman R. Sustained safety and performance of the second-generation drug-eluting absorbable metal scaffold in patients with de novo coronary lesions: 12-month clinical results and angiographic findings of the BIOSOLVE-II first-in-man trial. *Eur Heart J*.2016.
12. Robaei D, Back LM, Ooi SY, Pitney MR, Jepson N. Everolimus-eluting Bioresorbable Vascular Scaffold Implantation in Real World and Complex Coronary Disease: Procedural and 30-day outcomes at two Australian Centres. *Heart Lung Circ*.2015.
13. Waksman R, Lipinski MJ, Acampado E, Cheng Q, Adams L, Torii S, Gai J, Torguson R, Hellinga DM, Westman PC, Joner M, Zumstein P, Kolodgie FD, Virmani R. Comparison of Acute Thrombogenicity for Metallic and Polymeric Bioabsorbable Scaffolds: Magmaris Versus Absorb in a Porcine Arteriovenous Shunt Model. *Circ Cardiovasc Interv*.2017;10.
14. Waksman R, Zumstein P, Pritsch M, Wittchow E, Haude M, Lapointe-Corriveau C, Leclerc G, Joner M. Second-generation Magnesium Scaffold Magmaris, Device Design, and Preclinical Evaluation in a Porcine Coronary Artery Model. *EuroIntervention*.2017;13:440–9.
15. Schmidt W, Behrens P, Brandt-Wunderlich C, Siewert S, Grabow N, Schmitz KP, Schmidt W, Behrens P, Brandt-Wunderlich C, Siewert S, Grabow N, Schmitz K-P. In vitro performance investigation of bioresorbable scaffolds - Standard tests for vascular stents and beyond. *Cardiovasc Revasc. Med*.2016;17:375–83.
16. Erbel R, Böse D, Haude M, Kordish I, Churzidze S, Malyar N, Konorza T, Sack S. Absorbable coronary stents. New promising technology. *Herz*.2007;32:308–19.
17. Haude M, Erbel R, Erne P, Verheye S, Degen H, Vermeersch P, Weissman N, Prati F, Bruining N, Waksman R, Koolen J. Safety and performance of the DRug-Eluting Absorbable Metal Scaffold (DREAMS) in patients with de novo coronary lesions: 3-year results of the prospective, multicentre, first-in-man BIOSOLVE-I trial. *EuroIntervention*.2016;12:e160-e166.
18. Haude M, Ince H, Abazaid A, Toelg R, Lemos PA, Birgelen C von, Christiansen EH, Wijns W, Neumann F-J, Kaiser C, Eeckhout E, Lim ST, Escaned J, Garcia-Garcia HM, Waksman R. Safety and performance of the second-generation drug-eluting absorbable metal scaffold in patients with de-novo coronary artery lesions (BIOSOLVE-II): 6 month results of a prospective, multicentre, non-andomised, first-in-man trial. *Lancet*.2015;online:---.
19. Haude M, Ince H, Kische S, Abizaid A, Tölg R, Alves Lemos P, van Mieghem NM, Verheye S, Birgelen C von, Christiansen EH, Wijns W, Garcia-Garcia HM, Waksman R. Sustained safety and

clinical performance of a drug-eluting absorbable metal scaffold up to 24 months: Pooled outcomes of BIOSOLVE-II and BIOSOLVE-III. *EuroIntervention*.2017.

20. Haude M, Ince H, Toelg R, Lemos PA, Birgelen C von, Christiansen EH, Wijns W, Neumann F-J, Eeckhout E, Garcia-Garcia HM, Waksman R. Safety and performance of the second-generation drug-eluting absorbable metal scaffold (DREAMS 2G) in patients with de novo coronary lesions: Three-year clinical results and angiographic findings of the BIOSOLVE-II first-in-man trial. *EuroIntervention*.2020;15:e1375-e1382.
21. Haude M, Ince H, Kische S, Abizaid A, Tölg R, Alves Lemos P, van Mieghem NM, Verheye S, Birgelen C von, Christiansen EH, Barbato E, Garcia-Garcia HM, Waksman R. Safety and clinical performance of a drug eluting absorbable metal scaffold in the treatment of subjects with de novo lesions in native coronary arteries: Pooled 12-month outcomes of BIOSOLVE-II and BIOSOLVE-III. *Catheter Cardiovasc Interv*.2018.
22. Verheye S, Wlodarczak A, Montorsi P, Torzewski J, Bennett J, Haude M, Starmer G, Buck T, Wiemer M, Nuruddin AAB, Yan BP-Y, Lee MK-Y. BIOSOLVE-IV-registry: Safety and performance of the Magmaris scaffold: 12-month outcomes of the first cohort of 1,075 patients. *Catheter Cardiovasc Interv*.2020.
23. Wlodarczak A, Garcia LAI, Karjalainen PP, Komócsi A, Pisano F, Richter S, Lanocha M, Rumoroso JR, Leung KF. Magnesium 2000 postmarket evaluation: Guideline adherence and intraprocedural performance of a sirolimus-eluting resorbable magnesium scaffold. *Cardiovascular Revascularization Medicine*.2019.
24. Wlodarczak A, Lanocha M, Jastrzebski A, Pecherzewski M, Szudrowicz M, Jastrzebski W, Nawrot J, Lesiak M. Early outcome of magnesium bioresorbable scaffold implantation in acute coronary syndrome-the initial report from the Magmaris-ACS registry. *Catheter Cardiovasc Interv*.2018.
25. Blachutzik F, Achenbach S, Tröbs M, Marwan M, Weissner M, Nef H, Schlundt C. Effect of non-compliant balloon postdilatation on magnesium-based bioresorbable vascular scaffolds. *Catheter Cardiovasc Interv*.2018.
26. Hemptinne Q de, Picard F, Briki R, Awada A, Silance P-G, Dolatabadi D, Debbas N, Unger P. Drug-Eluting Resorbable Magnesium Scaffold Implantation in ST-Segment Elevation Myocardial Infarction: A Pilot Study. *J Invasive Cardiol*.2018;30:202–6.
27. Sabaté M, Alfonso F, Cequier A, Romaní S, Bordes P, Serra A, Iñiguez A, Salinas P, García del Blanco B, Goicolea J, Hernández-Antolín R, Cuesta J, Gómez-Hospital JA, Ortega-Paz L, Gomez-Lara J, Brugaletta S. Magnesium-Based Resorbable Scaffold Versus Permanent Metallic

Sirolimus-Eluting Stent in Patients With ST-Segment Elevation Myocardial Infarction: The MAGSTEMI Randomized Clinical Trial. *Circulation*.2019;140:1904–16.

28. Onuma Y, Ormiston J, Serruys PW. Bioresorbable scaffold technologies. *Circ J*.2011;75:509–20.
29. Onuma Y, Serruys PW. Bioresorbable Scaffold - The Advent of a New Era in Percutaneous Coronary and Peripheral Revascularization? *New Drugs an Technologies*.2011;-:779–94.
30. Heublein B, Rohde R, Kaese V, Niemeyer M, Hartung W, Haverich A. Biocorrosion of magnesium alloys: A new principle in cardiovascular implant technology? *Heart*.2003;89:651–6.
31. Waksman R, Pakala R, Kuchulakanti PK, Baffour R, Hellinga D, Seabron R, Tio FO, Wittchow E, Hartwig S, Harder C, Rohde R, Heublein B, Andreae A, Waldmann KH, Haverich A. Safety and efficacy of bioabsorbable magnesium alloy stents in porcine coronary arteries. *Catheter Cardiovasc Interv*.2006;68:607–17.
32. Peeters P, Bosiers M, Verbist J, Deloose K, Heublein B. Preliminary results after application of absorbable metal stents in patients with critical limb ischemia. *J Endovasc Ther*.2005;12:1–5.
33. Erbel R, Di MC, Bartunek J, Bonnier J, Bruyne B de, Eberli FR, Erne P, Haude M, Heublein B, Horrigan M, Ilsley C, Böse D, Koolen J, Luscher TF, Weissman N, Waksman R. Temporary scaffolding of coronary arteries with bioabsorbable magnesium stents: A prospective, non-randomised multicentre trial. *Lancet*.2007;369:1869–75.
34. Waksman R, Erbel R, Di MC, Bartunek J, Bruyne B de, Eberli FR, Erne P, Haude M, Horrigan M, Ilsley C, Böse D, Bonnier H, Koolen J, Luscher TF, Weissman NJ. Early- and long-term intravascular ultrasound and angiographic findings after bioabsorbable magnesium stent implantation in human coronary arteries. *JACC Cardiovasc Interv*.2009;2:312–20.
35. Squillace DMK. Bioresorbable drug-eluting magnesium-alloy scaffold: Design and feasibility in a porcine coronary model; 2005.
36. Serruys PW, Onuma Y, Garcia-Garcia HM, Muramatsu T, van Geuns RJ, Bruyne B de, Dudek D, Thuesen L, Smits PC, Chevalier B, McClean D, Koolen J, Windecker S, Whitbourn R, Meredith I, Dorange C, Veldhof S, Hebert KM, Rapoza R, Ormiston JA. Dynamics of vessel wall changes following the implantation of the absorb everolimus-eluting bioresorbable vascular scaffold: A multi-imaging modality study at 6, 12, 24 and 36 months. *EuroIntervention*.2014;9:1271–84.
37. Serruys PW, Onuma Y, Ormiston JA, Bruyne B de, Regar E, Dudek D, Thuesen L, Smits PC, Chevalier B, McClean D, Koolen J, Windecker S, Whitbourn R, Meredith I, Dorange C, Veldhof S, Miquel-Hebert K, Rapoza R, Garcia-Garcia HM. Evaluation of the second generation of a bioresorbable everolimus drug-eluting vascular scaffold for treatment of de novo coronary artery stenosis: Six-month clinical and imaging outcomes. *Circulation*.2010;122:2301–12.

38. Serruys PW, Onuma Y, Dudek D, Smits PC, Koolen J, Chevalier B, Bruyne B de, Thuesen L, McClean D, van Geuns RJ, Windecker S, Whitbourn R, Meredith I, Dorange C, Veldhof S, Hebert KM, Sudhir K, Garcia-Garcia HM, Ormiston JA. Evaluation of the second generation of a bioresorbable everolimus-eluting vascular scaffold for the treatment of de novo coronary artery stenosis: 12-month clinical and imaging outcomes. *JACC*.2011;58:1578–88.
39. Serruys PW, Ruygrok P, Neuzner J, Piek JJ, Seth A, Schofer JJ, Richardt G, Wiemer M, Carrie D, Thuesen L, Boone E, Miquel-Herbert K, Daemen J. A randomised comparison of an everolimus-eluting coronary stent with a paclitaxel-eluting coronary stent:the SPIRIT II trial. *EuroIntervention*.2006;2:286–94.
40. Serruys PW, Silber S, Garg S, van Geuns RJ, Richardt G, Buszman PE, Kelbaek H, van Boven AJ, Hofma SH, Linke A, Klauss V, Wijns W, Macaya C, Garot P, DiMario C, Manoharan G, Kornowski R, Ischinger T, Bartorelli A, Ronden J, Bressers M, Gobbens P, Negoita M, van Leeuwen F, Windecker S. Comparison of zotarolimus-eluting and everolimus-eluting coronary stents. *N Engl J Med*.2010;363:136–46.
41. Windecker S, Haude M, Neumann FJ, Stangl K, Witzenbichler B, Slagboom T, Sabate M, Goicolea J, Barragan P, Cook S, Piot C, Richardt G, Merkely B, Schneider H, Bilger J, Erne P, Waksman R, Zaugg S, Juni P, Lefevre T. Comparison of a novel biodegradable polymer sirolimus-eluting stent with a durable polymer everolimus-eluting stent: Results of the randomized BIOFLOW-II trial. *Circ Cardiovasc Interv*.2015;8:e001441.
42. Waltenberger J, Brachmann J, van der Heyden J, Richardt G, Frobert O, Seige M, Erglis A, Dewilde W, Winkens M, Hegeler-Molkewehrum C, Klein N, Hoffmann S. Real-world experience with a novel biodegradable polymer sirolimus-eluting stent: Twelve-month results of the BIOFLOW-III registry. *EuroIntervention*.2015;10.
43. Pilgrim T, Heg D, Roffi M, Tüller D, Muller O, Vuilliomenet A, Cook S, Weilenmann D, Kaiser C, Jamshidi P, Fahrni T, Moschovitis A, Noble S, Eberli FR, Wenaweser P, Jüni P, Windecker S. Ultrathin strut biodegradable polymer sirolimus-eluting stent versus durable polymer everolimus-eluting stent for percutaneous coronary revascularisation (BIOSCIENCE): A randomised, single-blind, non-inferiority trial. *Lancet*.2014;384:2111–22.
44. Saito S, Tölg R, Witzenbichler B, Haude M, Masotti M, Salmeron R, Witkowski A, Uematsu M, Takahashi A, Waksman R, Slagboom T. A Randomized, Intercontinental, Multicenter Study to Assess the Safety and Effectiveness of the Orsiro Sirolimus Eluting Stent in the Treatment of Subjects with de novo Coronary Artery Lesions BIOFLOW IV – Primary Outcome Target Vessel Failure at 12 Months. *EuroIntervention*.2019;15:e1006-e1013.

45. Kandzari DE, Mauri L, Koolen JJ, Massaro JM, Doros G, Garcia-Garcia HM, Bennett J, Roguin A, Gharib EG, Cutlip DE, Waksman R. Ultrathin, bioresorbable polymer sirolimus-eluting stents versus thin, durable polymer everolimus-eluting stents in patients undergoing coronary revascularisation (BIOFLOW V): A randomised trial. *Lancet*.2017.
46. Ormiston JA, Serruys PW, Regar E, Dudek D, Thuesen L, Webster MW, Onuma Y, Garcia-Garcia HM, McGreevy R, Veldhof S. A bioabsorbable everolimus-eluting coronary stent system for patients with single de-novo coronary artery lesions (ABSORB): A prospective open-label trial. *Lancet*.2008;371:899–907.
47. Karanasos A, Simsek C, Gnanadesigan M, van Ditzhuijzen NS, Freire R, Dijkstra J, Tu S, van Mieghem N, van Soest G, de Jaegere P, Serruys PW, Zijlstra F, van Geuns RJ, Regar E. OCT Assessment of the Long-Term Vascular Healing Response 5 Years After Everolimus-Eluting Bioresorbable Vascular Scaffold. *JACC*.2014;64:2343–56.
48. Serruys PW, Ormiston J, Onuma Y, Regar E, Gonzalo N. A bioabsorbable everolimus-eluting coronary stent system (ABSORB): 2-year outcomes and results from multiple imaging methods. *Lancet*.2009;373:897–910.
49. Onuma Y, Dudek D, Thuesen L, Webster M, Nieman K, Garcia-Garcia HM, Ormiston JA, Serruys PW. Five-Year Clinical and Functional Multislice Computed Tomography Angiographic Results After Coronary Implantation of the Fully Resorbable Polymeric Everolimus-Eluting Scaffold in Patients With De Novo Coronary Artery Disease The ABSORB Cohort A Trial. *Journal of the American College of Cardiology: Cardiovascular Intervention*.2013;6.
50. Fajadet J, Wijns W, Laarman GJ, Kuck KH, Ormiston J, Munzel T, Popma JJ, Fitzgerald PJ, Bonan R, Kuntz RE. Randomized, double-blind, multicenter study of the Endeavor zotarolimus-eluting phosphorylcholine-encapsulated stent for treatment of native coronary artery lesions: Clinical and angiographic results of the ENDEAVOR II trial. *Circulation*.2006;114:798–806.
51. Meredith IT. The Endeavor Drug-Eluting Stent program. In: Serruys PW, Gershlick AH, editors. Handbook of Drug-Eluting Stents: CRC Press; 2005. p. 305–12.
52. Wöhrle J, Nusser T, Hoffmann S, Kochs M. Angiographic results of the cobalt chromium Vision and Mini-Vision stents. *Can J Cardiol*.2009;25(10):581–4.
53. Diletti R, Farooq V, Girasis C, Bourantas C, Onuma Y, Heo JH, Gogas BD, van Geuns RJ, Regar E, Bruyne B de, Dudek D, Thuesen L, Chevalier B, McClean D, Windecker S, Whitbourn RJ, Smits P, Koolen J, Meredith I, Li X, Miquel-Hebert K, Veldhof S, Garcia-Garcia HM, Ormiston JA, Serruys PW. Clinical and intravascular imaging outcomes at 1 and 2 years after implantation of absorb everolimus eluting bioresorbable vascular scaffolds in small vessels. Late lumen

enlargement: Does bioresorption matter with small vessel size? Insight from the ABSORB cohort B trial. *Heart*.2013;99:98–105.

54. Chevalier B, Silber S, Park S-J, Garcia E, Schuler G, Suryapranata H, Koolen J, Hauptmann KE, Wijns W, Morice M-C, Carrie D, van Es G-A, Nagai H, Detiege D, Paunovic D, Serruys PW. Randomized comparison of the Nobori Biolimus A9-eluting coronary stent with the Taxus Liberté paclitaxel-eluting coronary stent in patients with stenosis in native coronary arteries: The NOBORI 1 trial--Phase 2. *Circ Cardiovasc Interv*.2009;2:188–95.
55. Serruys PW, Ormiston J, van Geuns RJ, Bruyne B de, Dudek D, Christiansen E, Chevalier B, Smits P, McClean D, Koolen J, Windecker S, Whitbourn R, Meredith I, Wasungu L, Ediebah D, Veldhof S, Onuma Y. A Polylactide Bioresorbable Scaffold Eluting Everolimus for Treatment of Coronary Stenosis: 5-Year Follow-Up. *JACC*.2016;67:766–76.
56. Serruys PW, Chevalier B, Dudek D, Cequier A, Carrie D, Iniguez A, Dominici M, van der Schaaf RJ, Haude M, Wasungu L, Veldhof S, Peng L, Staehr P, Grundeken MJ, Ishibashi Y, Garcia-Garcia HM, Onuma Y. A bioresorbable everolimus-eluting scaffold versus a metallic everolimus-eluting stent for ischaemic heart disease caused by de-novo native coronary artery lesions (ABSORB II): An interim 1-year analysis of clinical and procedural secondary outcomes from a randomised controlled trial. *Lancet*.2015;385:43–54.
57. Chevalier B, Cequier A, Dudek D, Haude M, Carrie D, Sabaté M, Windecker S, Reith S, Sousa Almeida M de, Campo G, Iñiguez A, Onuma Y, Serruys PW. Four-year follow-up of the randomised comparison between an everolimus-eluting bioresorbable scaffold and an everolimus-eluting metallic stent for the treatment of coronary artery stenosis (ABSORB II trial). *EuroIntervention*.2017.
58. Ellis SG, Kereiakes DJ, Metzger DC, Caputo RP, Rizik DG, Teirstein PS, Litt MR, Kini A, Kabour A, Marx SO, Popma JJ, McGreevy R, Zhang Z, Simonton C, Stone GW. Everolimus-Eluting Bioresorbable Scaffolds for Coronary Artery Disease. *N Engl J Med*.2015;373:1905–15.
59. Stone GW, Ellis SG, Gori T, Metzger DC, Stein B, Erickson M, Torzewski J, Williams J, Lawson W, Broderick TM, Kabour A, Piegari G, Cavendish J, Bertolet B, Choi JW, Marx SO, Généreux P, Kereiakes DJ. Blinded outcomes and angina assessment of coronary bioresorbable scaffolds: 30-day and 1-year results from the ABSORB IV randomised trial. *Lancet*.2018.
60. Costa JR, Abizaid A, Whitbourn R, Serruys PW, Jepson N, Steinwender C, Stuteville M, Ediebah D, Sudhir K, Bartorelli AL. Three-year clinical outcomes of patients treated with everolimus-eluting bioresorbable vascular scaffolds: Final results of the ABSORB EXTEND trial. *Catheter Cardiovasc Interv*.2019;93:E1-E7.

61. Capodanno D, Gori T, Nef H, Latib A, Mehilli J, Lesiak M, Caramanno G, Naber C, Di MC, Colombo A, Capranzano P, Wiebe J, Araszkiewicz A, Geraci S, Pyxaras S, Mattesini A, Naganuma T, Munzel T, Tamburino C. Percutaneous coronary intervention with everolimus-eluting bioresorbable vascular scaffolds in routine clinical practice: Early and midterm outcomes from the European multicentre GHOST-EU registry. *EuroIntervention*.2015;10:1144–53.
62. Tamburino C, Capranzano P, Gori T, Latib A, Lesiak M, Nef H, Caramanno G, Naber C, Mehilli J, Di MC, Sabate M, Munzel T, Colombo A, Araszkiewicz A, Wiebe J, Geraci S, Jensen C, Mattesini A, Brugaletta S, Capodanno D. 1-Year Outcomes of Everolimus-Eluting Bioresorbable Scaffolds Versus Everolimus-Eluting Stents: A Propensity-Matched Comparison of the GHOST-EU and XIENCE V USA Registries. *JACC Cardiovasc Interv*.2016;9:440–9.
63. Baumbach A. Absorb UK Registry: One year results of contemporary bioresorbable vascular scaffold implantation. In: euroPCR; 2017.
64. Testa L, Carlo M de, Petrolini A, Rapetto C, Varbella F, Cortese B, Gabrielli G, Geraci S, Loi B, Boccuzzi G, Tarantini G, Fischetti D, Calabria P, Tomai F, Ribichini F, Tamburino C, Indolfi C, Bartorelli AL, Petronio AS, Bedogni F. One-year clinical results of the Italian diffuse/multivessel disease ABSORB prospective registry (IT-DISAPPEARS). *EuroIntervention*.2017;13:424–31.
65. Hoppmann P, Kufner S, Cassese S, Wiebe J, Schneider S, Pinieck S, Scheler L, Bernlochner I, Joner M, Schunkert H, Laugwitz KL, Kastrati A, Byrne RA. Angiographic and clinical outcomes of patients treated with everolimus-eluting bioresorbable stents in routine clinical practice: Results of the ISAR-ABSORB registry. *Catheter Cardiovasc Interv*.2016;87:822–9.
66. Wiebe J, Dörr O, Ilstad H, Husser O, Liebetrau C, Boeder N, Bauer T, Möllmann H, Kastrati A, Hamm CW, Nef HM. Everolimus- Versus Novolimus-Eluting Bioresorbable Scaffolds for the Treatment of Coronary Artery Disease: A Matched Comparison. *JACC Cardiovasc Interv*.2017;10:477–85.
67. Cayla G, Koning R, Fajadet J, Sainsous J, Carrié D, Elhadad S, Tarragano F, Lefévre T, Ranc S, Ghostine S, Garot P, Marco F, Maillard L, Motreff P, Le Breton H. Percutaneous coronary interventions with the Absorb Bioresorbable vascular scaffold in real life: 1-year results from the FRANCE ABSORB registry. *Archives of Cardiovascular Diseases*.2019;112:113–23.
68. Cassese S, Byrne RA, Ndrepepa G, Kufner S, Wiebe J, Repp J, Schunkert H, Fusaro M, Kimura T, Kastrati A. Everolimus-eluting bioresorbable vascular scaffolds versus everolimus-eluting metallic stents: A meta-analysis of randomised controlled trials. *Lancet*.2015.
69. Serruys PW, Onuma Y. Bioresorbable scaffolds - From basic concept to clinical applications. Boca Raton, FL: CRC Press/Taylor & Francis Group; 2017.

70. Abizaid A, Costa RA, Schofer J, Ormiston J, Maeng M, Witzenbichler B, Botelho RV, Costa JRJ, Chamie D, Abizaid AS, Castro JP, Morrison L, Toyloy S, Bhat V, Yan J, Verheye S. Serial Multimodality Imaging and 2-Year Clinical Outcomes of the Novel DESolve Novolimus-Eluting Bioresorbable Coronary Scaffold System for the Treatment of Single De Novo Coronary Lesions. *JACC Cardiovasc Interv*.2016;9:565–74.
71. Nef H, Wiebe J, Boeder N, Dörr O, Bauer T, Hauptmann K-E, Latib A, Colombo A, Fischer D, Rudolph T, Foin N, Richardt G, Hamm C. A multicenter post-marketing evaluation of the Elixir DESolve®Novolimus-eluting bioresorbable coronary scaffold system: First results from the DESolve PMCF study. *Catheter Cardiovasc Interv*.2018.
72. Abizaid A, Carrié D, Frey N, Lutz M, Weber-Albers J, Dudek D, Chevalier B, Weng S-C, Costa RA, Anderson J, Stone GW. 6-Month Clinical and Angiographic Outcomes of a Novel Radiopaque Sirolimus-Eluting Bioresorbable Vascular Scaffold: The FANTOM II Study. *JACC Cardiovasc Interv*.2017;10:1832–8.
73. Lipinski MJ, Acampado E, Cheng Q, Adams L, Torii S, Gai J, Torguson R, Hellinga DG, Joner M, Harder C, Zumstein P, Finn AV, Kolodgie FD, Virmani R, Waksman R. Comparison of acute thrombogenicity for magnesium versus stainless steel stents in a porcine arteriovenous shunt model. *EuroIntervention*.2019;14:1420–7.
74. Neumann F-J, Sousa-Uva M, Ahlsson A, Alfonso F, Banning AP, Benedetto U, Byrne RA, Collet J-P, Falk V, Head SJ, Jüni P, Kastrati A, Koller A, Kristensen SD, Niebauer J, Richter DJ, Seferović PM, Sibbing D, Stefanini GG, Windecker S, Yadav R, Zembala MO, Wijns W, Glineur D, Aboyans V, Achenbach S, Agewall S, Andreotti F, Barbato E, Baumbach A, Brophy J, Bueno H, Calvert PA, Capodanno D, Davierwala PM, Delgado V, Dudek D, Freemantle N, Funck-Brentano C, Gaemperli O, Gielen S, Gilard M, Gorenek B, Haasenritter J, Haude M, Ibanez B, Iung B, Jeppsson A, Katritsis D, Knuuti J, Kolh P, Leite-Moreira A, Lund LH, Maisano F, Mehilli J, Metzler B, Montalescot G, Pagano D, Petronio AS, Piepoli MF, Popescu BA, Sádaba R, Shlyakhto E, Silber S, Simpson IA, Sparv D, Tavilla G, Thiele H, Tousek P, van Belle E, Vranckx P, Witkowski A, Zamorano JL, Roffi M, Coca A, Coman IM, Dean V, Fitzsimons D, Hindricks G, Katus HA, Lancellotti P, Leclercq C, McDonagh TA, Ponikowski P, Chettibi M, Sisakian H, İbrahimov F, Stelmashok VI, Postadzhiyan A, Skoric B, Eftychiou C, Kala P, Terkelsen CJ, Magdy A, Eha J, Niemelä M, Kedev S, Motreff P, Aladashvili A, Kanakakis I-G, Becker D, Gudnason T, Peace A, Romeo F, Bajraktari G, Kerimkulova A, Rudzītis A, Ghazzal Z, Kibarskis A, Pereira B, Xuereb RG, Hofma SH, Steigen TK, Oliveira EI de, Mot S, Duplyakov D, Zavatta M, Beleslin B, Kovar F, Bunc M, Ojeda S, Witt N, Jeger R, Addad F, Akdemir R, Parkhomenko

A, Henderson R. 2018 ESC/EACTS Guidelines on myocardial revascularization. *Eur Heart J*.2018;34:2949.

75. Fajadet J, Haude M, Joner M, Koolen J, Lee M, Tölg R, Waksman R. Magmaris preliminary recommendation upon commercial launch: A consensus from the expert panel on 14 April 2016. *EuroIntervention*.2016;12:828–33.
76. Byrne RA, Serruys PW, Baumbach A, Escaned J, Fajadet J, James S, Joner M, Oktay S, Juni P, Kastrati A, Sianos G, Stefanini GG, Wijns W, Windecker S. Report of a European Society of Cardiology-European Association of Percutaneous Cardiovascular Interventions task force on the evaluation of coronary stents in Europe: Executive summary. *Eur Heart J*.2015.
77. Verheye S, Wlodarczak A, Montorsi P, Bennett J, Torzewski J, Haude M, Vrolix M, Buck T, Aminian A, van der Schaaf RJ, Nuruddin AA, Lee MKY. Twelve-month outcomes of 400 patients treated with a resorbable metal scaffold: Insights from the BIOSOLVE-IV registry. *EuroIntervention*.2020;15:e1383-e1386.
78. Dash D. Complications of coronary intervention: Abrupt closure, dissection, perforation. *Heart Asia*.2013;5:61–5.
79. Kang SH, Chung WY, Lee JM, Park JJ, Yoon CH, Suh JW, Cho YS, Doh JH, Cho JM, Bae JW, Youn TJ, Chae IH. Angiographic outcomes of Orsiro biodegradable polymer sirolimus-eluting stents and Resolute Integrity durable polymer zotarolimus-eluting stents: Results of the ORIENT trial. *EuroIntervention*.2017;12:1623–31.
80. Markovic S, Lützner M, Dragomir S, Rottbauer W, Wöhrle J. Angiographic and clinical outcomes after recanalization of coronary chronic total occlusions with the Orsiro sirolimus-eluting stent compared with the resolute zotarolimus-eluting stent. *Coron Artery Dis*.2017;28:376–80.
81. Birgelen C von, Zocca P, Buiten RA, Jessurun GAJ, Schotborgh CE, Roguin A, Danse PW, Benit E, Aminian A, van Houwelingen KG, Anthonio RL, Stoel MG, Somi S, Hartmann M, Linssen GCM, Doggen CJM, Kok MM. Thin composite wire strut, durable polymer-coated (Resolute Onyx) versus ultrathin cobalt-chromium strut, bioresorbable polymer-coated (Orsiro) drug-eluting stents in allcomers with coronary artery disease (BIONYX): An international, single-blind, randomised non-inferiority trial. *Lancet*.2018.
82. Lee JM, Hwang D, Park J, Kim KJ, Ahn C, Koo BK. Percutaneous Coronary Intervention at Centers With and Without On-Site Surgical Backup: An Updated Meta-Analysis of 23 Studies. *Circulation*.2015;132:388–401.
83. Riley RF, Sapontis J, Kirtane AJ, Karmpaliotis D, Kalra S, Jones PG, Lombardi WL, Grantham JA, McCabe JM. Prevalence, predictors, and health status implications of periprocedural

complications during coronary chronic total occlusion angioplasty. *EuroIntervention*.2018;14:e1199-e1206.

84. Serruys PW, Chevalier B, Dudek D, Cequier A, Carrie D, Iniguez A, Dominici M, van der Schaaf RJ, Haude M, Wasungu L, Veldhof S, Peng L, Staehr P, Grundeken MJ, Ishibashi Y, Garcia-Garcia HM, Onuma Y, Serruys PW, Chevalier B, Dudek D, Cequier A, Carrié D, Iniguez A, Dominici M, van der Schaaf RJ, Haude M, Wasungu L, Veldhof S, Peng L, Staehr P, Grundeken MJ, Ishibashi Y, Garcia-Garcia HM, Onuma Y. A bioresorbable everolimus-eluting scaffold versus a metallic everolimus-eluting stent for ischaemic heart disease caused by de-novo native coronary artery lesions (ABSORB II): An interim 1-year analysis of clinical and procedural secondary outcomes from a randomised controlled trial. *Lancet*.2015;385:43–54.
85. Holubkov R, Laskey WK, Haviland A, Slater JC, Bourassa MG, Vlachos HA, Cohen HA, Williams DO, Kelsey SF, Detre KM. Angina 1 year after percutaneous coronary intervention: A report from the NHLBI Dynamic Registry. *Am Heart J*.2002;144:826–33.
86. Shimony A, Joseph L, Mottillo S, Eisenberg MJ. Coronary artery perforation during percutaneous coronary intervention: A systematic review and meta-analysis. *Can J Cardiol*.2011;27:843–50.
87. Guttmann OP, Jones DA, Gulati A, Kotecha T, Fayed H, Patel D, Crake T, Ozkor M, Wragg A, Smith EJ, Weerackody R, Knight CJ, Mathur A, O'Mahony C. Prevalence and outcomes of coronary artery perforation during percutaneous coronary intervention. *EuroIntervention*.2017;13:e595-e601.
88. Stathopoulos I, Jimenez M, Panagopoulos G, Kwak EJ, Losquadro M, Cohen H, Iyer S, Ruiz C, Roubin G, Garratt K. The decline in PCI complication rate: 2003-2006 versus 1999-2002. *Hellenic. J. Cardiol*.2009;50:379–87.
89. Aoki J, Kirtane A, Leon MB, Dangas G. Coronary artery aneurysms after drug-eluting stent implantation. *JACC Cardiovasc Interv*.2008;1:14–21.
90. Gorenek B, Lundqvist CB, Terradellas JB, Camm AJ, Hindricks G, Huber K, Kirchhof P, Kuck KH, Kudaiberdieva G, Lin T, Raviele A, Santini M, Tilz RR, Valgimigli M, Vos MA, Vrints C, Zeymer U. Cardiac arrhythmias in acute coronary syndromes: Position paper from the joint EHRA, ACCA, and EAPCI task force. *Eur Heart J Acute. Cardiovasc Care*.2015;4:386.
91. Albanese M, Alpaslan K, Ouarrak T, Merguet P, Schneider S, Schöls W. In-hospital major arrhythmias, arrhythmic death and resuscitation after successful primary percutaneous intervention for acute transmural infarction: A retrospective single-centre cohort study. *BMC Cardiovasc Disord*.2018;18:116.

92. Mehta RH, Harjai KJ, Grines L, Stone GW, Boura J, Cox D, O'Neill W, Grines CL. Sustained ventricular tachycardia or fibrillation in the cardiac catheterization laboratory among patients receiving primary percutaneous coronary intervention: Incidence, predictors, and outcomes. *JACC*.2004;43:1765–72.
93. Gayed M, Yadak N, Qamhia W, Daralammouri Y, Ohlow M-A. Comorbidities and Complications in Nonagenarians Undergoing Coronary Angiography and Intervention. *Int Heart J*.2017;58:180–4.
94. Verheye S, Ormiston JA, Stewart J, Webster M, Sanidas E, Costa R, Costa JRJ, Chamie D, Abizaid AS, Pinto I, Morrison L, Toyloy S, Bhat V, Yan J, Abizaid A. A next-generation bioresorbable coronary scaffold system: From bench to first clinical evaluation: 6- and 12-month clinical and multimodality imaging results. *JACC Cardiovasc Interv*.2014;7:89–99.
95. Haude M, Ince H, Abizaid A, Toelg R, Lemos PA, Birgelen C von, Christiansen EH, Wijns W, Neumann FJ, Kaiser C, Eeckhout E, Lim ST, Escaned J, Garcia-Garcia HM, Waksman R. Safety and performance of the second-generation drug-eluting absorbable metal scaffold in patients with de-novo coronary artery lesions (BIOSOLVE-II): 6 month results of a prospective, multicentre, non-randomised, first-in-man trial. *Lancet*.2016;387:31–9.
96. Ariza Solé A, Salazar-Mendiguchía J, Lorente-Tordera V, Sánchez-Salado JC, González-Costello J, Moliner-Borja P, Gómez-Hospital JA, Manito-Lorite N, Cequier-Fillat A. Invasive mechanical ventilation in acute coronary syndromes in the era of percutaneous coronary intervention. *Eur Heart J Acute Cardiovasc Care*.2013;2:109–17.
97. Lee MS, Canan T, Perlowski A, Bhatia R, Jurewitz D, Tobis JM. Causes of death in patients undergoing percutaneous coronary intervention with drug-eluting stents in a real-world setting. *J Invasive Cardiol*.2009;21:441–5.
98. Cowie MR, Wood DA, Coats AJ, Thompson SG, Poole-Wilson PA, Suresh V, Sutton GC. Incidence and aetiology of heart failure; a population-based study. *Eur Heart J*.1999;20:421–8.
99. Resnic FS, Majithia A, Marinac-Dabic D, Robbins S, Ssemaganda H, Hewitt K, Ponirakis A, Loyo-Berrios N, Moussa I, Drozda J, Normand S-L, Matheny ME. Registry-Based Prospective, Active Surveillance of Medical-Device Safety. *N Engl J Med*.2017;376:526–35.
100. Kelm M, Perings SM, Jax T, Lauer T, Schoebel FC, Heintzen MP, Perings C, Strauer BE. Incidence and clinical outcome of iatrogenic femoral arteriovenous fistulas: Implications for risk stratification and treatment. *JACC*.2002;40:291–7.
101. Rogers JH, Lasala JM. Coronary artery dissection and perforation complicating percutaneous coronary intervention. *J. Invasive. Cardiol*.2004;16:493–9.

102. Chevalier B, Abizaid A, Carrié D, Frey N, Lutz M, Weber-Albers J, Dudek D, Weng S-C, Akodad M, Anderson J, Stone GW. Clinical and Angiographic Outcomes With a Novel Radiopaque Sirolimus-Eluting Bioresorbable Vascular Scaffold. *Circ Cardiovasc Interv*.2019;12:e007283.
103. Hokimoto S, Tabata N, Sueta D, Akasaka T, Tsujita K, Sakamoto K, Kaikita K, Kojima S, Ogawa H. The real-world prevalence of cardiovascular events related to coronary spasm after percutaneous coronary intervention. *Journal of Cardiology*.2016;68:20–8.
104. Guerra E, Ndrepepa G, Schulz S, Byrne R, Hoppmann P, Kufner S, Ibrahim T, Tada T, Schunkert H, Laugwitz K-L, Kastrati A. Impact of inhospital stent thrombosis and cerebrovascular accidents on long-term prognosis after percutaneous coronary intervention. *Am Heart J*.2014;168:862-8.e1.
105. Byrne RA, Joner M, Kastrati A. Stent thrombosis and restenosis: what have we learned and where are we going? The Andreas Grüntzig Lecture ESC 2014. *Eur Heart J*.2015;36:3320–31.
106. Wiebe J, Nef HM, Hamm CW. Current Status of Bioresorbable Scaffolds in the Treatment of Coronary Artery Disease. *JACC*.2014;64:2541–51.
107. Karjalainen P, Paana T, Sia J, Nammas W. Neointimal Healing Evaluated by Optical Coherence Tomography after Drug-Eluting Absorbable Metal Scaffold Implantation in de novo Native Coronary Lesions: Rationale and Design of the Magmaris-OCT Study. *Cardiology*.2017;137:225–30.
108. Ang HY, Huang YY, Lim ST, Wong P, Joner M, Foin N. Mechanical behavior of polymer-based vs. metallic-based bioresorbable stents. *J Thorac Dis*.2017;9:S923-S934.
109. Gonzalo N, Macaya C. Absorbable stent: Focus on clinical applications and benefits. *Vasc. Health Risk Manag*.2012;8:125–32.
110. Zur Mühlen C von, Reiss S, Krafft AJ, Besch L, Menza M, Zehender M, Heidt T, Maier A, Pfannebecker T, Zirlik A, Reinöhl J, Stachon P, Hilgendorf I, Wolf D, Diehl P, Wengenmayer T, Ahrens I, Bode C, Bock M. Coronary magnetic resonance imaging after routine implantation of bioresorbable vascular scaffolds allows non-invasive evaluation of vascular patency. *PLOS ONE*.2018;13:e0191413.
111. Collet C, Chevalier B, Cequier A, Fajadet J, Dominici M, Helqvist S, van Boven AJ, Dudek D, McClean D, Almeida M, Piek JJ, Tenekecioglu E, Bartorelli A, Windecker S, Serruys PW, Onuma Y. Diagnostic Accuracy of Coronary CT Angiography for the Evaluation of Bioresorbable Vascular Scaffolds. *JACC Cardiovasc Imaging*.2017.
112. Opolski MP, Kepka C, Wojakowski W, Witkowski A. Computed tomography angiography for guiding and follow-up of magnesium-bioresorbable scaffold implantation. *Clin Res Cardiol*.2018.

113. Alfonso F, Fernandez-Vina F, Medina M, Hernandez R. Neoatherosclerosis: The missing link between very late stent thrombosis and very late in-stent restenosis. *JACC*.2013;61:e155.
114. Mishra S. A fresh look at bioresorbable scaffold technology: Intuition pumps. *Indian Heart J*.2017;69:107–11.
115. Joner M. Systemic versus site targeted treatment of neoatherosclerosis. 2017; Barcelona.
116. Ozaki Y, Garcia-Garcia HM, Hideo-Kajita A, Kuku KO, Haude M, Ince H, Abizaid A, Tölg R, Lemos PA, Birgelen C von, Christiansen EH, Wijns W, Escaned J, Dijkstra J, Waksman R. Impact of procedural characteristics on coronary vessel wall healing following implantation of second-generation drug-eluting absorbable metal scaffold in patients with de novo coronary artery lesions: An optical coherence tomography analysis. *Eur Heart J Cardiovasc Imaging*.2018.
117. Mauri L, Orav EJ, Kuntz RE. Late loss in lumen diameter and binary restenosis for drug-eluting stent comparison. *Circulation*.2005;111:3435–42.
118. Mauri L, Orav EJ, Candia SC, Cutlip DE, Kuntz RE. Robustness of late lumen loss in discriminating drug-eluting stents across variable observational and randomized trials. *Circulation*.2005;112:2833–9.
119. Hicks KA, Tcheng JE, Bozkurt B, Chaitman BR, Cutlip DE, Farb A, Fonarow GC, Jacobs JP, Jaff MR, Lichtman JH, Limacher MC, Mahaffey KW, Mehran R, Nissen SE, Smith EE, Targum SL. 2014 ACC/AHA Key Data Elements and Definitions for Cardiovascular Endpoint Events in Clinical Trials: A Report of the American College of Cardiology/American Heart Association Task Force on Clinical Data Standards (Writing Committee to Develop Cardiovascular Endpoints Data Standards). *JACC*.2015;66:403–69.
120. Serruys PW, van Hout B, Bonnier H, Legrand V, Garcia E, Macaya C, Sousa E, van der Giessen W, Colombo A, Seabra-Gomes R, Kiemeneij F, Ruygrok P, Ormiston J, Emanuelsson H, Fajadet J, Haude M, Klugmann S, Morel MA. Randomised comparison of implantation of heparin-coated stents with balloon angioplasty in selected patients with coronary artery disease (Benestent II) [published erratum appears in Lancet 1998 Oct 31;352(9138):1478]. *Lancet*.1998;352:673–81.
121. Pinto DS, Stone GW, Ellis SG, Cox DA, Hermiller J, O'Shaughnessy C, Mann JT, Mehran R, Na Y, Turco M, Caputo R, Popma JJ, Cutlip DE, Russell ME, Cohen DJ. Impact of routine angiographic follow-up on the clinical benefits of paclitaxel-eluting stents: Results from the TAXUS-IV trial. *JACC*.2006;48:32–6.
122. Cutlip DE, Chauhan MS, Baim DS, Ho KK, Popma JJ, Carrozza JP, Cohen DJ, Kuntz RE. Clinical restenosis after coronary stenting: Perspectives from multicenter clinical trials. *JACC*.2002;40:2082–9.

123. Cutlip DE, Windecker S, Mehran R, Boam A, Cohen DJ, van Es GA, Steg PG, Morel MA, Mauri L, Vranckx P, McFadden E, Lansky A, Hamon M, Krucoff MW, Serruys PW. Clinical end points in coronary stent trials: A case for standardized definitions. *Circulation*.2007;115:2344–51.
124. Gomez-Lara J, Brugaletta S, Farooq V, Onuma Y, Diletti R, Windecker S, Thuesen L, McClean D, Koolen J, Whitbourn R, Dudek D, Smits PC, Chevalier B, Regar E, Veldhof S, Rapoza R, Ormiston JA, Garcia-Garcia HM, Serruys PW. Head-to-head comparison of the neointimal response between metallic and bioresorbable everolimus-eluting scaffolds using optical coherence tomography. *JACC Cardiovasc Interv*.2011;4:1271–80.
125. Tenekecioglu E, Serruys PW, Onuma Y, Costa R, Chamié D, Sotomi Y, Yu T-B, Abizaid A, Liew H-B, Santoso T. Randomized Comparison of Absorb Bioresorbable Vascular Scaffold and Mirage Microfiber Sirolimus-Eluting Scaffold Using Multimodality Imaging. *JACC Cardiovasc Interv*.2017;10:1115–30.
126. Goldman AI. Issues in designing sequential stopping rules for monitoring side effects in clinical trials. *Control Clin. Trials*.1987;8:327–37.
127. Hamm CW, Braunwald E. A classification of unstable angina revisited. *Circulation*.2000;102:118–22.
128. Reynolds HR, Hochman JS. Cardiogenic shock: Current concepts and improving outcomes. *Circulation*.2008;117:686–97.
129. Gibbons RJ, Abrams J, Chatterjee K, Daley J, Deedwania PC, Douglas JS, Ferguson TBJ, Fihn SD, Fraker TDJ, Gardin JM, O'Rourke RA, Pasternak RC, Williams SV, Alpert JS, Antman EM, Hiratzka LF, Fuster V, Faxon DP, Gregoratos G, Jacobs AK, Smith SCJ. ACC/AHA 2002 guideline update for the management of patients with chronic stable angina--summary article: A report of the American College of Cardiology/American Heart Association Task Force on Practice Guidelines (Committee on the Management of Patients With Chronic Stable Angina). *Circulation*.2003;107:149–58.
130. Fraker TDJ, Fihn SD, Gibbons RJ, Abrams J, Chatterjee K, Daley J, Deedwania PC, Douglas JS, Ferguson TBJ, Gardin JM, O'Rourke RA, Williams SV, Smith SCJ, Jacobs AK, Adams CD, Anderson JL, Buller CE, Creager MA, Ettinger SM, Halperin JL, Hunt SA, Krumholz HM, Kushner FG, Lytle BW, Nishimura R, Page RL, Riegel B, Tarkington LG, Yancy CW. 2007 chronic angina focused update of the ACC/AHA 2002 Guidelines for the management of patients with chronic stable angina: A report of the American College of Cardiology/American Heart Association Task Force on Practice Guidelines Writing Group to develop the focused update of

the 2002 Guidelines for the management of patients with chronic stable angina. *Circulation*.2007;116:2762–72.

131. FDA, CDRH, Center for Drug Evaluation and Research (CDER). Coronary Drug-Eluting Stents - Nonclinical and Clinical Studies. Companion Document. DRAFT GUIDANCE , 6255 companion.doc 4/7/2008 ; 2008.
132. Garcia-Garcia HM, McFadden EP, Farb A, Mehran R, Stone GW, Spertus J, Onuma Y, Morel M-A, van Es G-A, Zuckerman B, Fearon WF, Taggart D, Kappetein A-P, Krucoff MW, Vranckx P, Windecker S, Cutlip D, Serruys PW. Standardized End Point Definitions for Coronary Intervention Trials: The Academic Research Consortium-2 Consensus Document. *Circulation*.2018;137:2635–50.
133. Smith SCJ, Dove JT, Jacobs AK, Kennedy JW, Kereiakes D, Kern MJ, Kuntz RE, Popma JJ, Schaff HV, Williams DO, Gibbons RJ, Alpert JP, Eagle KA, Faxon DP, Fuster V, Gardner TJ, Gregoratos G, Russell RO. ACC/AHA guidelines for percutaneous coronary intervention (revision of the 1993 PTCA guidelines)-executive summary: A report of the American College of Cardiology/American Heart Association task force on practice guidelines (Committee to revise the 1993 guidelines for percutaneous transluminal coronary angioplasty) endorsed by the Society for Cardiac Angiography and Interventions. *Circulation*.2001;103:3019–41.
134. Thygesen K, Alpert JS, Jaffe AS, Simoons ML, Chaitman BR, White HD. Third universal definition of myocardial infarction. *JACC*.2012;60:1581–98.
135. Thygesen K, Alpert JS, White HD. Universal definition of myocardial infarction. *Eur Heart J*.2007;28:2525–38.
136. Thygesen K, Alpert JS, Jaffe AS, Chaitman BR, Bax JJ, Morrow DA, White HD, Mickley H, Crea F, van de Werf F, Bucciarelli-Ducci C, Katus HA, Pinto FJ, Antman EM, Hamm CW, Caterina R de, Januzzi JL, Apple FS, Alonso Garcia MA, Underwood SR, Canty JM, Lyon AR, Devereaux PJ, Zamorano JL, Lindahl B, Weintraub WS, Newby LK, Virmani R, Vranckx P, Cutlip D, Gibbons RJ, Smith SC, Atar D, Luepker RV, Robertson RM, Bonow RO, Steg PG, O'Gara PT, Fox KAA, Hasdai D, Aboyans V, Achenbach S, Agewall S, Alexander T, Avezum A, Barbato E, Bassand J-P, Bates E, Bittl JA, Breithardt G, Bueno H, Bugiardini R, Cohen MG, Dangas G, Lemos JA de, Delgado V, Filippatos G, Fry E, Granger CB, Halvorsen S, Hlatky MA, Ibanez B, James S, Kastrati A, Leclercq C, Mahaffey KW, Mehta L, Müller C, Patrono C, Piepoli MF, Piñeiro D, Roffi M, Rubboli A, Sharma S, Simpson IA, Tendera M, Valgimigli M, van der Wal AC, Windecker S, Chettibi M, Hayrapetyan H, Roithinger FX, Aliyev F, Sujayeva V, Claeys MJ, Smajić E, Kala P, Iversen KK, El Hefny E, Marandi T, Porela P, Antov S, Gilard M,

Blankenberg S, Davlouros P, Gudnason T, Alcalai R, Colivicchi F, Elezi S, Baitova G, Zakke I, Gustiene O, Beissel J, Dingli P, Grosu A, Damman P, Juliebø V, Legutko J, Morais J, Tatu-Chitoiu G, Yakovlev A, Zavatta M, Nedeljkovic M, Radsel P, Sionis A, Jemberg T, Abid L, Abaci A, Parkhomenko A, Corbett S. Fourth universal definition of myocardial infarction (2018). *Eur Heart J*.2018;28:97.

137. Vranckx P, Cutlip DE, Mehran R, Kint PP, Silber S, Windecker S, Serruys PW. Myocardial infarction adjudication in contemporary all-comer stent trials: Balancing sensitivity and specificity. Addendum to the historical MI definitions used in stent studies. *EuroIntervention*.2010;5:871–4.
138. Moussa ID, Klein LW, Shah B, Mehran R, Mack MJ, Brilakis ES, Reilly JP, Zoghbi G, Holper E, Stone GW. Consideration of a new definition of clinically relevant myocardial infarction after coronary revascularization: An expert consensus document from the Society for Cardiovascular Angiography and Interventions (SCAI). *JACC*.2013;62:1563–70.
139. Little B&C. The Criteria Committee of the New York Heart Association, Nomenclature and Criteria for Diagnosis of Diseases of the Heart and Great Vessels. In: ; 1994. p. 253–6.
140. Caicedo MS, Solver E et al. Metal sensitivities among TJApatients iwth pot-operative pain: indications for multi-metal LTT testing. J Long Term Eff med Implants 214; 24: 37-44.
