# Supplementary material for: Safety and performance of the third-generation drug-eluting resorbable coronary magnesium scaffold system in the treatment of subjects with de novo coronary artery lesions: 6-month results of the prospective, multicenter BIOMAG-I first-in-human study
Source: eClinicalMedicine. 2023 Apr 17;59:101940. doi: 10.1016/j.eclinm.2023.101940 (PMC10126775; doi:10.1016/j.eclinm.2023.101940)
Supplement: Supplementary Fig. 1 and Tables S1–S6 [file mmc2.docx]

**Safety and performance of the third-generation drug-eluting resorbable coronary magnesium scaffold system in the treatment of subjects with de-novo coronary artery lesions: 6-month results from the prospective, multicentre BIOMAG-I first-in-human study**

**Supplemental File 1: Clinical Investigation Plan**

**Supplemental File 2: Supplemental Material**

**Supplemental Table 1: List of study centers**

| **Country** | **Site** |
| --- | --- |
| Austria | Medizinische Universität Graz |
| Belgium | A.Z. Middelheim |
| Belgium | Ziekenhuis Oost Limburg Genk (ZOL Genk) |
| Belgium | UZ Leuven |
| Switzerland | Hôpitaux Universitaires Genève (HUG) |
| Spain | Hospital Clinico San Carlos, Madrid |
| Germany | Dt. Herzzentrum München |
| Germany | Segeberger Kliniken, Bad Segeberg |
| Germany | Rheinlandklinikum Lukaskrankenhaus Neuss |
| Germany | Johannes Wesling Klinikum Minden |
| Germany | Klinikum Kempten Oberallgaeu |
| Poland | Miedziowe Centrum Zdrowia SA (Copper Center) |
| Sweden | Skane University Hospital Lund |

**Supplemental Table 2: In- and Exclusion Criteria**

| Inclusion criteria | 1. Subject is > 18 years and < 80 years of age 2. Written subject informed consent available prior to PCI 3. Subject eligible for PCI, according to the 2018 ESC/EACTS Guidelines on myocardial revascularisation. 4. Subjects with a maximum of two single lesions in two separate coronary arteries which have to be de novo lesions and can be covered with 1 device each 5. Reference vessel diameter between 2·5 - 4·2 mm by visual estimation, depending on the scaffold size used 6. Target lesion length ≤ 28 mm by visual estimation, depending on the scaffold size used 7. Target lesion stenosis by visual estimation > 50% - < 100% and TIMI flow ≥1 (assisted by e.g. QCA / IVUS /FFR). 8. Subjects with stable or unstable angina pectoris or documented silent ischemia or hemodynamically stable NSTEMI patients without angiographic evidence of thrombus at target lesion 9. Subject who has no contraindication for DAPT |
| --- | --- |
| Exclusion criteria | 1. Pregnant or breast-feeding females or females who intend to become pregnant during the time of the study 2. Subject has clinical symptoms and ECG changes consistent with acute STEMI within 72 hours prior to the index procedure (NOTE: after 72 hours, any lesion other than the one causing the acute STEMI (culprit lesion) in any other epicardial vessel, may be treated according to the inclusion and exclusion criteria 3. Left main coronary artery disease 4. Three-vessels with coronary artery disease requiring treatment at time of procedure, including: left main, left anterior descending artery (LAD) right coronary artery (RCA) and circumflex coronary artery (Cx) 5. Planned interventional treatment of any non-target vessel within 12-month post-procedure 6. Subjects on dialysis 7. Impaired renal function (serum creatinine > 2·5 mg/dl or 221 μmol/l, determined within 72 hours prior to intervention) 8. Planned future intervention of a second lesion within the target vessel. 9. Ostial target lesion (within 5·0 mm of vessel origin) 10. Target lesion involves a side branch >2·0 mm in diameter 11. Documented left ventricular ejection fraction (LVEF) ≤ 30% within the last 6 months 12. Heavily calcified lesion which cannot be adequately pre-dilated by a non-compliant and/ or scoring balloon as described in exclusion criteria 15. 13. Target lesion is located in or supplied by an arterial or venous bypass graft 14. Target lesion requiring treatment with a device other than the non-compliant pre-dilatation balloon or scoring balloon prior to scaffold placement (including but not limited to rotational atherectomy, etc.) 15. Unsuccessful pre-dilatation, defined as a residual stenosis rate more than 20%, estimated by any method and/or angiographic complications (e.g. distal embolization, side branch closure, extensive dissections) 16. Known allergies or intolerances to: Acetylsalicylic Acid (ASA), P2Y12 inhibitors, Heparin, Contrast medium, Sirolimus, or similar drugs; or the scaffold material (Magnesium, Aluminium) 17. Subject is receiving an oral or intravenous immunosuppressive therapy (e.g., inhaled steroids are not excluded) or has a known life-limiting immunosuppressive or autoimmune disease (e.g., human immunodeficiency virus, systemic lupus erythematosus) diabetes mellitus is not excluded) 18. Life expectancy less than 1 year 19. Subjects under oral anticoagulation therapy (OAC) prior to implantation of DREAMS 3G unless DAPT can be maintained for a minimum of 6-month (recommendation: If a subject requires OAC after DREAMS 3G implantation, DAPT should be maintained until 6 months follow up. Afterwards DAPT can be downsized to either ASA or Clopidogrel alone together with OAC for the remaining time period up to 12 months. After this, OAC monotherapy can be prescribed if still required. 20. Planned surgery or dental surgical procedure within 6 months after index procedure unless DAPT will be maintained 21. In the investigators opinion, subject will not be able to comply with the follow-up requirements 22. Subject is currently participating in another study with an investigational device or an investigational drug and has not reached the primary endpoint yet |

DAPT-dual antiplatelet therapy, ECG-electrocardiogram, FFR-fractional flow reserve, IVUS-intravascular ultrasound, NSTEMI-non-ST elevation myocardial infarction, QCA-quantitative coronary angiography, PCI-percutaneous coronary intervention, STEMI-ST-elevation myocardial infarction, TIMI-thrombolysis in myocardial infarction

**Supplemental Table 3: Summary of core laboratory instructions**

| Imaging acquisition flow-chart |  |
| --- | --- |
| Angiography | Intracoronary nitroglycerine (100-200µg), two orthogonal views that should match at the various time intervals. |
| IVUS | Automatic pullback without interruption (uniform pullback speed of 0.5 mm/sec).  The pullback should begin approximately as far distal to the target segment as possible – up to 10 mm (the transducer of the IVUS catheter needs to be advanced beyond the target segment or just distal to the target, -as far as the IVUS auto pullback capability allows-, before starting the pullback). The pullback should extend till when the transducer is in the guiding catheter.  Pullback speed was 0·5 mm/s.  In the pre-percutaneous coronary intervention IVUS, dimensions of the lumen and external elastic membrane were measured every 1 mm with validated analysis software (QIVUS; Medis, Leiden, The Netherlands). After percutaneous coronary intervention and at follow-up, the contours of scaffold, lumen, and external elastic membrane were drawn.  Neointima was calculated as scaffold minus lumen measures. Incomplete scaffold apposition was defined as one or more scaffold struts clearly separated from the vessel wall with evidence of blood speckles behind the strut without overlapping side branches. |
| OCT | Pullback speed of 18 mm/s or 36 mm/s for the total length of the vessel segment.  During the pullback, automated injector such as ACIST was mandatory to be used for quality reason, with an injection rate of >4·0-5·0ml/s for the left coronary artery and >3·0-4 ml/s for the right coronary artery, depending on the vessel size.  The pullback must contain the in-segment (scaffolded segment and 5mm proximal and distal edges); and should extend at least 10 mm distal to scaffolded edge (i.e. 5 mm distal to in-segment).  The acquisition should extend until the distal radiopaque marker of the OCT catheter reaches the tip of the guiding catheter.  Analysis of contiguous cross-sections at 1 mm longitudinal intervals within the scaffolded segment was done offline with dedicated software (QIVUS; MEDIS, Leiden, The Netherlands). The contours of scaffold and lumen were drawn post-percutaneous coronary intervention. The number of scaffold struts was determined in each cross section. Struts were classified as apposed (if the strut was in contact with the vessel wall) or malapposed (if protruding into the lumen at a distance greater than the strut thickness). At follow-up, the scaffold was no more visible and therefore no scaffold contour was drawn. |

IVUS-intravascular ultrasound, OCT-optical coherence tomography

**Supplemental Table 4: Endpoint Definitions**

| Clinically-driven | Clinically-driven revascularisations are those in which the subjects have a positive functional clinical investigation, ischemic ECG changes at rest in a distribution consistent with the target vessel, or ischemic symptoms and an in-lesion diameter stenosis ≥ 50% by QCA. Revascularisation of a target lesion with an in-lesion diameter stenosis ≥70% (by QCA) in the absence of the above-mentioned ischemic signs or symptoms is also considered clinically-driven. In the absence of QCA data for relevant follow-up angiograms, the clinical need for revascularisation is adjudicated using the presence or absence of ischemic signs and symptoms. |
| --- | --- |
| Device expansion index | Minimum device area/ reference lumen area (1). Overexpansion was defined as a device expansion index >1, optimal expansion as 0·8-1·0, and underexpansion as <0·8. |
| Diameter stenosis | %diameter stenosis = [1 – (minimum lumen diameter/interpolated mean reference vessel diameter)] × 100 (2) |
| Eccentricity index | Minimum device diameter / maximum device diameter in a frame (the average of all eccentricity indices of each frame within a scaffolded segment is calculated) (2) |
| In-scaffold | Proximal to distal edge of the implanted scaffold. |
| In-segment | In-scaffold plus 5 mm proximal and 5 mm distal. |
| Late lumen loss | Post-procedural minimum lumen diameter (MLD) minus follow-up MLD determined by quantitative angiograph (1)y. MLD is derived from two orthogonal views, if available. |
| Target lesion revascularisation | Repeat revascularisation, including bypass surgery, within the in-segment area. |
| Target vessel revascularisation | Revascularisation of any segment of the index coronary artery, which was in physical  contact with any component (guiding catheter, guide wire, balloon catheter, etc.) of the  angioplasty hardware during the initial procedure. |

MLD-minimal lumen diameter, QCA-quantitative coronary angiography

**Supplemental Table 5: Sample Size calculation**

| **Primary endpoint** | |
| --- | --- |
| In-scaffold LLL at 6 months | ABSORB (v1.0) 0·44 ± 0·35 mm (3)  ABSORB (v1.1) 0·19 ± 0·18 mm (4)  DESolve 0·19 ± 0·19 mm (5)  Magmaris 0·44 ± 0·36 mm (6)  DESolve 0·20 ± 0·32 mm (7)  Fantom II 0·25 ± 0.40 mm (8) |
| Weighted mean in-scaffold LLL | 0·29 mm |
| Power | 0·95% |
| Alpha | 0·025 |
| Non-Inferiority Margin | 0·145 |
| Standard Deviation | 0·34 |
| Drop-out rate | 15% |
| Calculated number of patients | 88 (74 subjects + 15% drop out) |
| Null and alternative hypotheses for non-inferiority testing | 𝐻_0_: 𝜇_1_ ≥ 𝜇_01_ + Δ  𝐻_𝑎_: 𝜇_1_ < 𝜇_01_ + Δ  𝜇_1_ is the mean in-scaffold LLL of DREAMS 3G at 6 months and 𝜇_01_ the historical control (weighted mean) |
| **Post-hoc superiority analysis** |  |
| In-scaffold LLL at 6 months | Magmaris 0·44 ± 0·36 mm (6) |
| Power | 93·6% |
| Superiority margin | 0·11 mm (25% of mean LLL) |
| **Post-hoc non-inferiority analysis** | ABSORB (v1.1) 0·19 ± 0·18 mm (4)  DESolve 0·19 ± 0·19 mm (5)  DESolve 0·20 ± 0·32 mm (7)  Fantom II 0·25 ± 0.40 mm (8) |
| Weighted mean in-scaffold LLL | 0·22 mm |
| Power | 93·6% |
| Non-inferiority margin | 0·11 (50%) |
| **Secondary endpoint** | |
| In-scaffold LLL at 12 months | ABSORB 0·27 ± 0·32 mm (9)  Magmaris 0·39 ± 0·34 mm (10)  Fantom II 0·29 ± 0·36 mm (11)  Mirage vs ABSORB 0·37 mm and 0·23 mm (12) |
| Weighted mean in-scaffold LLL | 0·33 mm |
| Power | 0·95 |
| Alpha | 0·025 |
| Non-Inferiority Margin | 0·145 |
| Standard Deviation | 0·35 |
| Drop-out rate | 25% |
| Calculated number of patients | 104 (78 subjects + 25% drop out) |
| Null and alternative hypotheses for non-inferiority testing | 𝐻_0_: 𝜇_2_ ≥ 𝜇_02_ + Δ  𝐻_𝑎_: 𝜇_2_< 𝜇_02_ + Δ  𝜇2 is the mean in-scaffold LLL of DREAMS 3G at 12 months and 𝜇_02_the historical control (weighted mean) |

LLL-late lumen loss

**Supplemental Table 6: Baseline parameters of historical controls versus BioMag-I**

| **Parameter** | **BioMag-I** | **Absorb V1.0** | **Absorb V1.1** | **Desolve I** | **Magmaris** | **Desolve II** | **Fantom** | **Pooled Dataset*** | **P-value** |
| --- | --- | --- | --- | --- | --- | --- | --- | --- | --- |
| **N subjects** | 116 | 30 | 45 | 16 | 123 | 126 | 240 | 580 |  |
| **Corelab** | Corelab | Not reported | Core lab | Core lab | Core lab | Core lab | Core lab | Simulated | - |
| **Age, years** | 61 (9.0) | 62 (9) | 65 (9) | 69 (8.4) | 65 (10.3) | 62 (10) | 63 (10.1) | V1: 64 (9.6)  V2: 64 (10.3) | V1: 0.007**  V2: 0.009** |
| **Hypertension** | 74.1% | 60% | 60% | 62.5% | 82.1% | 71% | 73.8% | 72.8% | 0.760 |
| **Hyperlipidemia** | 62.1% | 63% | 93% | 68.8% | 60.2% | 71% | 70.8% | 69.8% | 0.101 |
| **Diabetes** | 27.6% | 3% | 13% | 6.3% | 29.3% | 21% | 23.8% | 21.9% | 0.183 |
| **History of smoking** | 64.7% | - | - | 68.8% | 54.5% | - | 59.6% | 58.3% | 0.223 |
| **History of myocardial infarction** | 33.6% | 3% | 36% | 25.0% | 23.6% | 44% | 26.3% | 29.0% | 0.317 |
| **NSTEMI** | 20.7% | 0% | 0% | 0% | 0% | 0% | 0% | 0% | <0.001 |
| **N lesions** | 117 | 31 | 45 | 14 | 123 | 122 | 238 | 573 |  |
| **Type B2/C lesions** | 76.9% | 0% | 52% | 37.5% | 43.4% | 34% | 31.9% | 34.9% | <0.001 |
| **MLD** | 1.06 (0.39) | 1.10 (0.26) | 1.06 (0.32) | 0.81 (0.29) | 1.22 (0.31) | 0.91 (0.38) | 0.82 (0.31) | V1: 0.94 (0.32)  V2: 0.96 (0.38) | V1: <0.001**  V2: 0.012** |
| **Lesion length, mm** | 12.3 (5.07) | 8.66 (3.99) | 10.2 (3.9) | 8.9 (2.64) | 12.6 (4.53) | 11.2 (3.75) | 11.6 (3.89) | V1: 11.37 (3.77)  V2: 11.55 (4.01) | V1: 0.064  V2: 0.136 |
| **Reference vessel diameter, mm** | 2.72 (0.46) | 2.72 (0.47) | 2.65 (0.46) | 2.65 (0.32) | 2.68 (0.40) | 3.00 (0.3) | 2.71 (0.37) | V1: 2.75 (0.37)  V2: 2.76 (0.39) | V1: 0.409  V2: 0.374 |

*Absorb, Desolve, Magmaris, Fantom – Limitation: no patient-level data available from studies other than for BioMag-I and Magmaris, therefore for continuous data assumption of normal distribution was applied to stimulate patient data for comparison..** not clinically relevant. V1: Weighted mean, weighted SD, > simulation of data assuming normal distribution with weighted mean and weighted SD. V2:simulation of data of each study assuming normal distribution with reported mean and SD

**Supplemental Table 7: Subgroup analysis for in-scaffold late lumen loss at six months**

|  | **BIOMAG-I**  **In-scaffold LLL [mm]** | **P-value** |
| --- | --- | --- |
| NSTEMI, N=22  No NSTEMI, N=89 | 0·17 (0·25)  0·10 (0·02; 0·33)  0·23 (0·32)  0·13 (0·5; 0·31) | 0·522 |
| Type B2/ C lesions, N=86  Type A/ B1 lesions, N=25 | 0·21 (0·33)  0·13 (0·04; 0·28)  0·21 (0·19)  0·19 (0·06; 0·38) | 0·478 |
| Age > 64 years, N=39  Age ≤ 64 years, N=72 | 0·25 (0·36)  0·12 (0·05; 0·33)  0·19 (0·28)  0·14 (0·04; 0·31) | 0·774 |
| MLD  ≤ 0.95 mm, N=42  >0.95 mm, N=69 | 0·26 (0·35)  0·18 (0·05; 0·36)  0·19 (0·27)  0·10 (0·05; 0·28) | 0·236 |

Data are displayed as mean (SD) and median (Q1;Q3). The threshold for age and MLD was selected to reflect the weighted mean of the pooled dataset displayed in Supplemental Table 5. Determined by quantitative coronary angiography (core laboratory analysis). LLL-late lumen loss, MLD-minimum lumen diameter, NSTEMI-non-ST-elevation myocardial infarction

**Supplemental Figure 1: Post-procedural device expansion index**


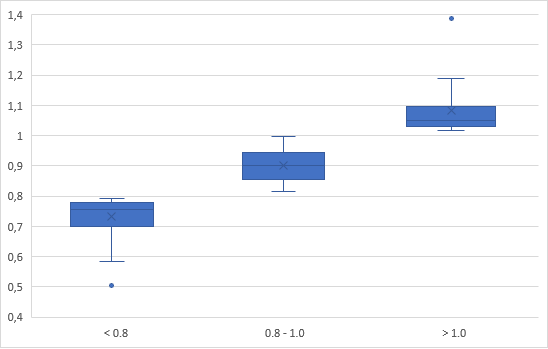


Defined as Minimum device area/ reference lumen area (1). The box represents the interquartile range, the “x”, the mean value, and the whiskers the minimum and maximum values except the outliers.

**Supplemental Table 8: Pharmacotherapy at discharge and follow-up**

|  | **Discharge**  **N=116** | **6 months**  **N=115*** |
| --- | --- | --- |
| ASA only | 0 (0·0%) | 0 (0·0%) |
| Clopidogrel only | 1 (0·9%) | 1 (0·9%) |
| DAPT   - *Thereof DAPT with additional anticoagulants* | 114 (98·3%)  6 (5·2%) | 112 (97·4%)  4 (3·5%) |
| Anticoagulants plus Clopidogrel | 1 (0·9%) | 2 (1·7%) |

Data are presented as n/data available (%). *One patient refused to return to the 6-month follow-up. ASA-acetylsalicylic acid, DAPT-dual antiplatelet therapy

**Supplemental Table 9: Target-vessel myocardial infarction at 6 months according to different definitions**

| **Definition**  **Peri-procedural MI** | **Definition**  **Spontaneous MI** | **6-month target-vessel MI** |
| --- | --- | --- |
| SCAI (13) | 3^rd^ Universal (14) | 0 (0·0%) |
| SCAI-(13) | 4^th^ Universal (15) | 0 (0·0%) |
| SCAI (13) | Extended historical (16) | 0 (0·0%) |
| ARC-2(17) | 3^rd^ Universal (14) | 1 (0·9%) |
| ARC-2 (17) | 4^th^ Universal (15) | 1 (0·9%) |
| ARC-2(17) | Extended historical (16) | 1 (0·9%) |

Data are based on Kaplan-Meier estimates. ARC-Academic Research Consortium, MI-myocardial infarction

References

1. Suwannasom P, Sotomi Y, Ishibashi Y, et al. The Impact of Post-Procedural Asymmetry, Expansion, and Eccentricity of Bioresorbable Everolimus-Eluting Scaffold and Metallic Everolimus-Eluting Stent on Clinical Outcomes in the ABSORB II Trial. *JACC Cardiovasc Interv*. 2016;9:1231-1242.

2 Garcia-Garcia HM, Serruys PW, Campos CM, et al. Assessing bioresorbable coronary devices: methods and parameters. *JACC Cardiovasc Imaging*. 2014;**7**:1130-1148.

3. Ormiston JA, Serruys PW, Regar E, et al. A bioabsorbable everolimus-eluting coronary stent system for patients with single de-novo coronary artery lesions (ABSORB): a prospective open-label trial. *Lancet.* 2008;**371**:899-907.

4. Serruys PW, Onuma Y, Ormiston JA, et al. Evaluation of the second generation of a bioresorbable everolimus drug-eluting vascular scaffold for treatment of de novo coronary artery stenosis: six-month clinical and imaging outcomes*. Circulation*. 2010;**122**:2301-2312.

5. Verheye S, Ormiston JA, Stewart J, et al. A next-generation bioresorbable coronary scaffold system: from bench to first clinical evaluation: 6- and 12-month clinical and multimodality imaging results. *JACC Cardiovasc Interv*. 2014;**7**:89-99.

6. Haude M, Ince H, Abizaid A, et al. Sustained safety and performance of the second-generation drug-eluting absorbable metal scaffold in patients with de novo coronary lesions: 12-month clinical results and angiographic findings of the BIOSOLVE-II first-in-man trial. *Eur Heart J*. 2016;**37**:2701-2709.

7. Abizaid A, Costa RA, Schofer J, et al. Serial Multimodality Imaging and 2-Year Clinical Outcomes of the Novel DESolve Novolimus-Eluting Bioresorbable Coronary Scaffold System for the Treatment of Single De Novo Coronary Lesions. *JACC Cardiovasc Interv*. 2016;**9**:565-574.

8. Chevalier B, Abizaid A, Carrié D, et al. Clinical and Angiographic Outcomes With a Novel Radiopaque Sirolimus-Eluting Bioresorbable Vascular Scaffold. *Circ Cardiovasc Interv.* 2019;**12**:e007283.

9. Serruys PW, Onuma Y, Dudek D, et al. Evaluation of the second generation of a bioresorbable everolimus-eluting vascular scaffold for the treatment of de novo coronary artery stenosis: 12-month clinical and imaging outcomes. *J Am Coll Cardiol*. 2011;**58**:1578-1588.

10. Haude M, Ince H, Kische S, et al. Safety and clinical performance of a drug eluting absorbable metal scaffold in the treatment of subjects with de novo lesions in native coronary arteries: Pooled 12-month outcomes of BIOSOLVE-II and BIOSOLVE-III. *Catheter Cardiovasc Interv.* 2018;**92**:E502-e511.

11. Gomez-Lara J, Brugaletta S, Farooq V, et al. Head-to-head comparison of the neointimal response between metallic and bioresorbable everolimus-eluting scaffolds using optical coherence tomography. *JACC Cardiovasc Interv*. 2011;**4**:1271-1280.

12. Tenekecioglu E, Serruys PW, Onuma Yet al. Randomized Comparison of Absorb Bioresorbable Vascular Scaffold and Mirage Microfiber Sirolimus-Eluting Scaffold Using Multimodality Imaging. *JACC Cardiovasc Interv.* 2017;**10**:1115-1130.

13. Moussa ID, Klein LW, Shah B, et al. Consideration of a new definition of clinically relevant myocardial infarction after coronary revascularization: an expert consensus document from the Society for Cardiovascular Angiography and Interventions (SCAI). *J Am Coll Cardiol*. 2013;**62**:1563-1570.

4 Thygesen K, Alpert JS, Jaffe AS, et al. Third universal definition of myocardial infarction. *J Am Coll Cardiol*. 2012;**60**:1581-1598.

15. Thygesen K, Alpert JS, Jaffe AS, et al. Fourth Universal Definition of Myocardial Infarction (2018). *J Am Coll Cardiol.* 2018;**72**:2231-2264.

6 Vranckx P, Cutlip DE, Mehran R, et al. Myocardial infarction adjudication in contemporary all-comer stent trials: balancing sensitivity and specificity. Addendum to the historical MI definitions used in stent studies. *EuroIntervention*. 2010;**5**:871-874.

17. Garcia-Garcia HM, McFadden EP, Farb A, et al. Standardized End Point Definitions for Coronary Intervention Trials: The Academic Research Consortium-2 Consensus Document. *Eur Heart J.* 2018;39:2192-207.
